# Bioassay-Guided Isolation and Identification of Antibacterial Compounds from Invasive Tree of Heaven Stem and Trunk Bark

**DOI:** 10.3390/molecules29245846

**Published:** 2024-12-11

**Authors:** Anna Cselőtey, Márton Baglyas, Nóra Király, Péter G. Ott, Vesna Glavnik, Irena Vovk, Ágnes M. Móricz

**Affiliations:** 1 Plant Protection Institute, HUN-REN Centre for Agricultural Research, Fehérvári út 132–144, 1116 Budapest, Hungary; cselotey.anna@atk.hun-ren.hu (A.C.); baglyas.marton@atk.hun-ren.hu (M.B.); norikiraly@yahoo.com (N.K.); ott.peter@atk.hun-ren.hu (P.G.O.); 2 Doctoral School, Semmelweis University, Üllői út 26, 1085 Budapest, Hungary; 3 Laboratory for Food Chemistry, National Institute of Chemistry, Hajdrihova 19, 1000 Ljubljana, Slovenia; vesna.glavnik@ki.si (V.G.); irena.vovk@ki.si (I.V.)

**Keywords:** tree of heaven (*Ailanthus altissima*), fatty acid derivatives, canthin-6-one alkaloid, TLC–effect-directed analysis, bioassay-guided isolation, antibacterial effect

## Abstract

Flash column chromatographic fractionation of tree of heaven (*Ailanthus altissima*) stem and trunk bark extracts, guided by thin-layer chromatography (TLC)–*Bacillus subtilis* assay and TLC–heated electrospray high-resolution tandem mass spectrometry (HESI-HRMS/MS), lead to the isolation of six known compounds: (9*Z*,11*E*)-13-hydroxy-9,11-octadecadienoic acid (13-HODE, **A1**), (10*E*,12*Z*)-9-hydroxy-10,12-octadecadienoic acid (9-HODE, **A2**), hexadecanedioic acid (thapsic acid, **A3**), 16-hydroxyhexadecanoic acid (juniperic acid, **A4**), 16-feruloyloxypalmitic acid (alpinagalanate, **A5**), and canthin-6-one (**A6**). Their structures were elucidated by HESI-HRMS/MS and one- and two-dimensional nuclear magnetic resonance (NMR) spectroscopy. This is the first study identifying **A1**–**A5** in *A. altissima* tree. Except for **A5**, all isolated compounds exhibited antibacterial activity against *B. subtilis* in microdilution assays. **A6** showed the strongest effect with a minimum inhibitory concentration (MIC) value of 8.3 µg/mL. The antibacterial activity of **A3** and **A4** is newly described.

## 1. Introduction

The discovery of antibiotics was one of the greatest scientific breakthroughs of the 20th century, and healthcare today would be unsustainable without these life-saving drugs [1]. However, in many cases their usage is not properly controlled, and careless overuse and misuse contribute to the rise in antibiotic resistance, which has become a major global health concern [2,3,4,5]. Unfortunately, the discovery of new antibiotics has slowed down in recent years, failing to keep pace with the growing emergence of multiresistant pathogens; therefore, research on these substances is urgently needed [1]. Natural products have been used as therapeutic agents by humanity since ancient times and have played a crucial role in the development of numerous pharmaceuticals, such as artemisinin and resveratrol [6]. Our knowledge of plant natural products is still very limited, despite their proven potential as a source of many promising drug leads, including antibiotics. Their strength lies in their easy accessibility, complex and unique chemodiversity, and diverse antibacterial modes of action, which makes plant natural products promising candidates for future research [7,8].

Tree of heaven (*Ailanthus altissima* (Mill.) Swingle) is a deciduous tree belonging to the family *Simaroubaceae* and the genus *Ailanthus* that consists of 15 different species. It is a medium-sized tree, reaching up to 27–30 m in height in the temperate zone, featuring smooth gray bark and pinnately compound leaves [9]. The species is native to Southeast Asia and is currently present on most continents except Antarctica [10]. It was first introduced to Europe in the 18th century as an ornamental tree and to prevent soil erosion, but due to its rapid growth and high reproduction ability, nowadays, it is considered one of the most invasive species across Europe as in other regions [11].

The areas most affected by *A. altissima* are suburban and rural sites, particularly along roadsides and railways, but it also colonizes forests and riverbanks where it poses a major threat to the local biodiversity [9,12]. The tree endangers indigenous plant communities through its aggressive spread driven by high seed production, vegetative reproduction via root sprouts, and the release of allelochemicals [13,14]. Where the species is present, local vegetation also becomes less diverse with a prevalence of more widely distributed taxa, the original plant community cannot fully recover two years after its removal [15]. The tree can also pose a potential risk to human health as its pollen may trigger allergic reactions, and prolonged exposure to its bark can lead to contact dermatitis [16,17,18]. To control its excessive spread, various management strategies have been employed, including mechanical, chemical, and biological methods, with varying levels of success [10].

The dried bark of *A. altissima* has been used in traditional Asian medicine for thousands of years to treat a variety of illnesses, including asthma, epilepsy, bleeding, fever, infections, and both ophthalmic and gastrointestinal diseases [19]. A wide range of biologically active compounds such as alkaloids, quassinoids, phenylpropanoids, triterpenoids, and essential oils have already been identified and characterized in the bark of the tree [19]. Among these compounds, the most studied are ailanthone, a quassinoid, and the alkaloid canthin-6-one, that both exhibit antitumor and anti-inflammatory effects [20,21,22,23]. Additionally, the extract of the bark also showed antiviral, herbicidal, and insecticidal activities [24,25,26]. Thin-layer chromatography (TLC) combined with effect-directed analysis (EDA) is a cost- and time-effective hyphenated method, enabling non-targeted, high-throughput screening for antibiotics, antioxidants, and enzyme inhibitors in complex matrices, including plant extracts [27,28,29]. The use of TLC–EDA can be followed by highly targeted characterization of the designated bioactive compounds with spectrometric and spectroscopic methods, such as high-resolution mass spectrometry (HRMS) and nuclear magnetic resonance (NMR) spectroscopy [30,31]. This approach enables the discovery of new bioactive compounds [32], new natural sources [33] or previously undescribed activities [34] of a known compound.

This study aimed to screen, isolate, and identify the antibacterial compounds present in the crude extracts of *A. altissima* trunk and stem bark. The bioactive compounds were detected by TLC–*Bacillus subtilis* bioassay and were characterized by chemical reagents, TLC–UV/Vis/FLD–heated electrospray ionization (HESI)-tandem high-resolution mass spectrometry (HRMS/MS), and flow injection analysis (FIA)–HESI-HRMS/MS. The bioassay-guided flash chromatographic fractionation and purification of bioactive compounds was followed by structure elucidation of the isolates using NMR spectroscopy and HRMS/MS. The anti-*Bacillus* activity of the six isolates was confirmed by a microdilution assay determining their minimum inhibitory concentration (MIC).

## 2. Results and Discussion

### 2.1. Detection and Isolation of Antibacterial Compounds

**Figure 1:**
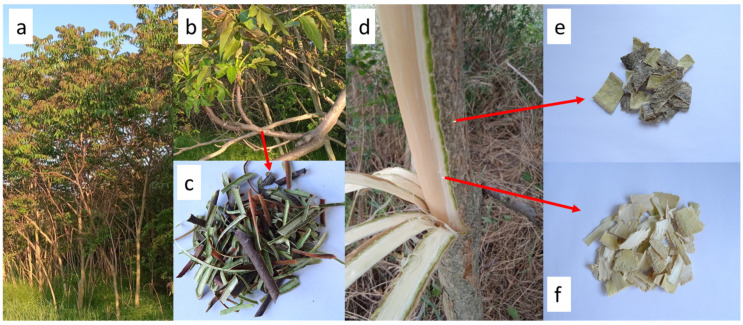
Sample collection from *Ailanthus altissima* tree (**a**), young branches (**b**), stem bark from young branches (**c**), bark from the tree trunk (**d**), outer trunk bark, (**e**) and inner trunk bark (**f**).

**Figure 2:**
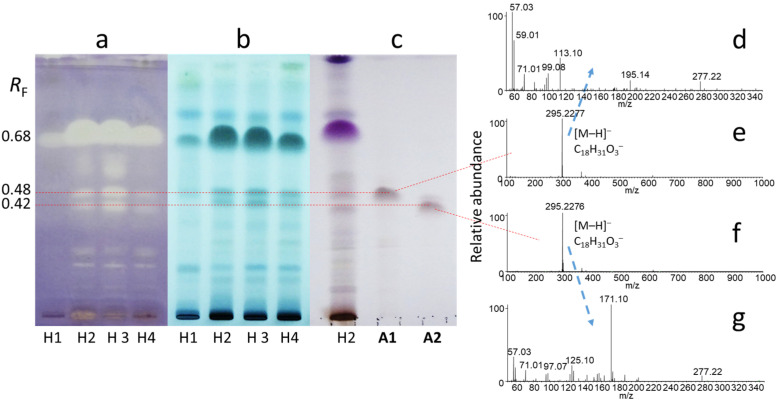
TLC–*B. subtilis* bioautogram (**a**) and TLC chromatograms after derivatization with molybdatophosphoric acid reagent (**b**) and *p*-anisaldehyde reagent (**c**) of stem bark extracts (H1–H4) developed with toluene–isopropyl acetate–methanol 5:4:1 *V*/*V*, as well as TLC–HESI^−^-HRMS (**e**,**f**) and TLC–HESI^−^-HRMS/MS (parent ions: *m*/*z* 295.2277 and *m*/*z* 295.2276, respectively; normalized HCD collision energy: 30%) (**d**,**g**) spectra of the isolated compounds (**A1**, **A2**). Stem bark samples H1–H4 were collected in Harta on 2 May 2022, 16 May 2022, 30 May 2022, and 3 July 2022, respectively.

**Figure 3:**
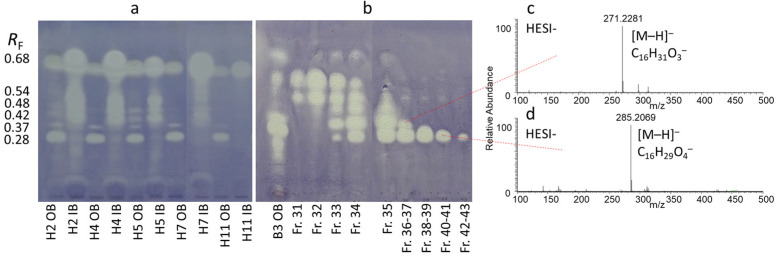
TLC–*B. subtilis* bioautograms of inner trunk bark (IB) and outer trunk bark (OB) samples (H2, H4, H5, H7, H11) (**a**), and the flash chromatography fractions (Fr. 31–43) of the outer bark extract B3 OB (**b**) developed with toluene–isopropyl acetate–methanol 5:4:1 *V/V*, as well as TLC–HESI^−^-HRMS spectra recorded from the zones of compounds **A3** (**d**) and **A4** (**c**). Trunk bark samples H2, H4, H5, H7, and H11 were collected in Harta on 16 May 2022, 3 July 2022, 13 August 2022, 24 October 2022, and 10 April 2023, respectively.

The methanolic extracts of the stem bark of young branches as well as inner and outer trunk bark (Figure 1) contained a range of chromatographic zones with antibacterial activity, which were revealed through the TLC–*B. subtilis* bioassays (Figure 2a–c and Figure 3a,b).

For the stem bark extracts (samples H1–H4), three major inhibitory zones were detected at *R*_F_ 0.48 (corresponding to compound **A1**), 0.42 (compound **A2**), and 0.68 that were visualized after derivatization both with molybdatophosphoric acid and *p*-anisaldehyde reagents (Figure 2a–c). *p*-Anisaldehyde is a universal reagent (many groups of compounds can be visualized with it), but molybdatophosphoric acid reagent is suitable for the detection of lipids, fatty acids, and steroids [35]. In the zones at *R*_F_ 0.68, a mixture of linoleic acid, linolenic acid, and palmitic acid were present, which were identified by comparison with standards and TLC–HRMS, and their antibacterial effects have previously been reported [36,37]. Interestingly, **A1** and **A2** were more abundant in the stem bark during the second half of May (Figure 2), coinciding with the flowering period of the tree.

**Figure 4:**
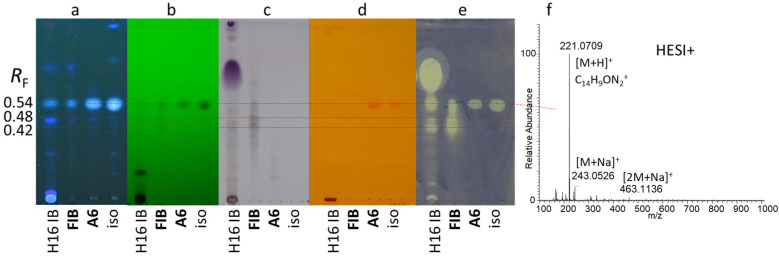
TLC chromatograms (**a**–**d**) of the inner bark sample used for fractionation (H16 IB) along with its flash fraction (**FIB**, Fr. 31–36) and isolated compound (**A6**), as well as a root isolate (iso, identical to **A6**), detected at 366 nm (**a**), 254 nm (**b**), after derivatization with *p*-anisaldehyde reagent (**c**) and Dragendorff’s reagent (**d**), and TLC–*B. subtilis* bioautogram (**e**) developed with toluene–isopropyl acetate–methanol 5:4:1 *V*/*V*, and TLC–HESI^+^-HRMS spectrum of compound **A6** (**f**).

Five characteristic bioactive zones were detected at *R*_F_ 0.28, 0.37, 0.42, 0.48, and 0.54 in extracts of the trunk bark (Figure 3a). The intensity of the chromatographic zones with antibacterial activity varied between outer and inner bark samples, showing a maximum intensity in samples obtained during late spring and summer (Figure 3a). The fractionation and isolation process of outer trunk bark extract revealed that inhibition zones at *R*_F_ 0.28 and 0.37 corresponded to compounds **A3** and **A4**, respectively (Figure 3b). Furthermore, during the fractionation, another inhibition zone was observed at *R*_F_ 0.47 related to compound **A5** (Figure S1). Fraction **FIB** and compound **A6** (at *R*_F_ 0.54) were obtained from the fractionation of inner bark extract (Figure 4a–e). Based on chromatographic and mass spectrometric results, fraction **FIB** was found to be rich in compounds **A1** (at *R*_F_ 0.48) and **A2** (at *R*_F_ 0.42), while **A6** was identical to a component previously isolated in our laboratory from the root of tree of heaven (not yet published) with a UV activity at 254 nm, a blue fluorescence at 366 nm, and a positive Dragendorff’s test (orange-brown spot), suggesting the presence of an alkaloid.

The fractionation of methanolic crude extracts and isolation of antibacterial compounds were performed by preparative flash column chromatography, guided by TLC–*B. subtilis* bioassay. Compounds **A1** (5.6 mg) and **A2** (4.3 mg) were isolated from the stem bark of young branches, **A3** (6.5 mg), **A4** (4.1 mg), and **A5** (0.8 mg) from the outer trunk bark, and **A6** (9.1 mg) from the inner trunk bark. Compounds **A1**–**A5** were used for subsequent structure elucidation. Instead of **A6**, the previously isolated, identical root compound was analyzed by NMR spectroscopy.

### 2.2. NMR Results

The NMR spectra (Figures S5–S10, S14–S19, S22–S27, S30–S35, S37–S42 and S44–S49) were recorded and the results were listed as follows (see Figure 5 for atomic numbering).

(9*Z*,11*E*)-13-hydroxy-9,11-octadecadienoic acid (**A1**): ^1^H NMR (600 MHz, CDCl_3_) *δ* 6.49 (ddd, *J* = 15.2, 11.1, 1.2 Hz, 1H, H-11), 5.97 (t, *J* = 10.9 Hz, 1H, H-10), 5.66 (dd, *J* = 15.2, 6.7 Hz, 1H, H-12), 5.44 (dt, *J* = 10.9, 7.7 Hz, 1H, H-9), 4.18 (q, *J* = 6.6 Hz, 1H, H-13), 2.34 (t, *J* = 7.4 Hz, 2H, H-2), 2.17 (m, 2H, H-8), 1.63 (p, *J* = 7.3 Hz, 2H, H-3), 1.54 (m, 2H, H-14), 1.38 (p, *J* = 6.6 Hz, 2H, H-7), 1.31 (m, 12H, H-4–H-6, H-15–H-17), 0.89 (t, *J* = 6.7 Hz, 3H, H-18); ^13^C NMR (151 MHz, CDCl_3_) *δ* 178.0 (C, C-1), 135.9 (CH, C-12), 133.0 (CH, C-9), 128.0 (CH, C-10), 125.9 (CH, C-11), 73.1 (CH, C-13), 37.4 (CH_2_, C-14), 33.8 (CH_2_, C-2), 31.9 (CH_2_, C-16), 29.4 (CH_2_, C-7), 28.9 (CH_2_, C-4–C-6), 27.7 (CH_2_, C-8), 25.3 (CH_2_, C-15), 24.8 (CH_2_, C-3), 22.7 (CH_2_, C-17), 14.2 (CH_3_, C-18).

(10*E*,12*Z*)-9-hydroxy-10,12-octadecadienoic acid (**A2**): ^1^H NMR (600 MHz, CDCl_3_) *δ* 6.48 (ddt, *J* = 15.2, 11.0, 1.1 Hz, 1H, H-11), 5.97 (tt, *J* = 11.1, 1.8 Hz, 1H, H-12), 5.66 (dd, *J* = 15.1, 6.9 Hz, 1H, H-10), 5.45 (dt, *J* = 10.9, 7.6 Hz, 1H, H-13), 4.15 (qd, *J* = 6.3, 2.4 Hz, 1H, H-9), 2.34 (t, *J* = 7.5 Hz, 2H, H-2), 2.18 (m, 2H, H-14), 1.63 (p, *J* = 7.4 Hz, 2H, H-3), 1.54 (m, 2H, H-8), 1.38 (p, *J* = 7.3 Hz, 2H, H-15), 1.30 (m, 12H, H-4–H-7, H-16, H-17), 0.89 (t, *J* = 6.7 Hz, 3H, H-18); ^13^C NMR (151 MHz, CDCl_3_) *δ* 178.2 (C, C-1), 135.8 (CH, C-10), 133.3 (CH, C-13), 127.8 (CH, C-12), 126.1 (CH, C-11), 73.1 (CH, C-9), 37.4 (CH_2_, C-8), 33.9 (CH_2_, C-2), 31.6 (CH_2_, C-16), 29.5, 29.4, 29.3 (CH_2_, C-5, C-6, C-15), 29.1 (CH_2_, C-4), 27.9 (CH_2_, C-14), 25.5 (CH_2_, C-7, C-15), 24.8 (CH_2_, C-3), 22.7 (CH_2_, C-17), 14.2 (CH_3_, C-18).

Hexadecanedioic acid (**A3**): ^1^H NMR (600 MHz, CD_3_OD) *δ* 2.27 (t, *J* = 7.4 Hz, 4H, H-2, H-15), 1.60 (p, *J* = 7.1 Hz, 4H, H-3, H-14), 1.30 (br s, 20H, H-4–H-13); ^13^C NMR (151 MHz, CD_3_OD) *δ* 177.8 (C, C-1, C-16), 35.0 (CH_2_, C-2, C-15), 30.7–30.3 (CH_2_, C-4–C-13), 26.1 (CH_2_, C-3, C-14).

16-Hydroxyhexadecanoic acid (**A4**): ^1^H NMR (600 MHz, CD_3_OD) *δ* 3.54 (t, *J* = 6.7 Hz, 2H, H-16), 2.27 (t, *J* = 7.5 Hz, 2H, H-2), 1.60 (p, *J* = 7.3 Hz, 2H, H-3), 1.53 (p, *J* = 6.6 Hz, 2H, H-15), 1.35 (m, 2H, H-14), 1.30 (br s, 20H, H-4–H-13); ^13^C NMR (151 MHz, CD_3_OD) *δ* 178.0 (C, C-1), 63.0 (CH_2_, C-16), 35.2 (CH_2_, C-2), 33.7 (CH_2_, C-15), 30.8–30.3 (CH_2_, C-4–C-13), 27.0 (CH_2_, C-14), 26.2 (CH_2_, C-3).

Alpinagalanate (**A5**): ^1^H NMR (600 MHz, CDCl_3_) *δ* 7.61 (d, *J* = 15.9 Hz, 1H, H-7′), 7.07 (dd, *J* = 8.8, 2.6 Hz, 1H, H-6′), 7.03 (d, *J* = 1.9 Hz, 1H, H-2′), 6.92 (d, *J* = 8.2 Hz, 1H, H-5′), 6.29 (d, *J* = 15.9 Hz, 1H, H-8′), 4.19 (t, *J* = 6.7 Hz, 2H, H-1), 3.93 (s, 3H, 3′–OCH_3_), 2.34 (t, *J* = 7.5 Hz, 2H, H-15), 1.69 (p, *J* = 6.9 Hz, 2H, H-2), 1.63 (p, *J* = 7.3 Hz, 2H, H-14), 1.39 (m, 1H, H-3), 1.25 (br s, 20H); ^13^C NMR (151 MHz, CDCl_3_) *δ* 176.1 (C, C-16), 167.6 (C, C-9′), 148.0 (C, C-4′), 146.9 (C, C-3′), 144.8 (CH, C-7′), 127.2 (C, C-1′), 123.2 (CH, C-6′), 115.8 (CH, C-8′), 114.8 (CH, C-5′), 109.4 (CH, C-2′), 64.8 (CH_2_, C-1), 56.1 (3′–OCH_3_), 33.5 (CH_2_, C-15), 29.9–29.2 (CH_2_, C-4–C-13), 28.9 (CH_2_, C-2), 26.1 (CH_2_, C-3), 24.9 (CH_2_, C-14).

Canthin-6-one (**A6**): ^1^H NMR (600 MHz, CDCl_3_) *δ* 8.83 (d, *J* = 5.0 Hz, 1H, H-2), 8.68 (dt, *J* = 8.2, 0.9 Hz, 1H, H-8), 8.12 (ddd, *J* = 7.8, 1.2, 0.7 Hz, 1H, H-11), 8.03 (d, *J* = 9.8 Hz, 1H, H-4), 7.97 (d, *J* = 5.0 Hz, 1H, H-1), 7.71 (ddd, *J* = 8.3, 7.4, 1.2 Hz, 1H, H-9), 7.54 (td, *J* = 7.6, 1.0 Hz, 1H, H-10), 6.99 (d, *J* = 9.8 Hz, 1H, H-5); ^13^C NMR (151 MHz, CDCl_3_) *δ* 159.7 (C, C-6), 146.0 (CH, C-2), 139.8 (CH, C-4), 139.7 (C, C-7), 136.4 (C, C-3), 132.3 (C, C-14), 131.1 (CH, C-9), 130.5 (C, C-13), 129.1 (CH, C-5), 125.8 (CH, C-10), 124.6 (C, C-12), 122.9 (CH, C-11), 117.5 (CH, C-8), 116.6 (CH, C-1).

### 2.3. Structure Elucidation of the Isolates

The recorded HRMS(/MS) spectra (Figures S2–S4, S11–S13, S20, S21, S28, S29, S36 and S43) and the NMR results were compiled for the structure elucidation of isolated compounds.

(9*Z*,11*E*)-13-hydroxy-9,11-octadecadienoic acid (**A1**) was obtained as a white amorphous solid. Its molecular formula was established as C_18_H_32_O_3_ deduced from the sodium adduct peak at *m*/*z* 319.2243 [M+Na]^+^ and the deprotonated molecule peak at *m*/*z* 295.2277 [M–H]^−^ in the HESI-HRMS spectra (Figure 2e and Figures S2 and S3), requiring three double-bond equivalents (DBEs). The ^1^H NMR spectrum of compound **A1** displayed signals for four olefinic protons at *δ*_H_ 6.49 (ddd, *J* = 15.2, 11.1, 1.2 Hz, 1H, H-11), 5.97 (t, *J* = 10.9 Hz, 1H, H-10), 5.66 (dd, *J* = 15.2, 6.7 Hz, 1H, H-12), 5.44 (dt, *J* = 10.9, 7.7 Hz, 1H, H-9), one oxygenated methine proton at *δ*_H_ 4.18 (q, *J* = 6.6 Hz, 1H, H-13), eleven methylenes, and one methyl group at *δ*_H_ 0.89 (t, *J* = 6.7 Hz, 3H, H-18). The ^13^C DEPTQ spectrum aided by the HSQC data of **A1**, resolved only 17 carbon resonances corresponding to one carboxylic carbon at *δ*_C_ 178.0 (C-1), four olefinic carbons at *δ*_C_ 135.9 (C-12), 133.0 (C-9), 128.0 (C-10), 125.9 (C-11), one oxygenated methine at *δ*_C_ 73.1 (C-13), eleven methylenes, and one methyl group at *δ*_C_ 14.2 (C-18). One C=O and two C=C double bonds account for altogether three DBEs, suggesting an acyclic compound. Based on the 1D NMR data, compound **A1** was hypothesized as a hydroxylated, unsaturated fatty acid. ^1^H–^1^H COSY correlations between H-9/H-10, H-10/H-11, H-11/H-12, and H-12/H-13 indicated a –CH(OH)–CH=CH–CH=CH– structural unit. However, the hydroxyl group in the aliphatic chain could not be located by 1D and 2D NMR data due to the highly overlapped methylene proton signals. MS/MS spectrum of **A1** (Figure 2d and Figure S4) revealed main fragment peak at *m*/*z* 195.1392 ([M–H–C_6_H_12_O]^−^, C_12_H_19_O_2_^−^) corresponding to the alpha cleavage showing the position of hydroxyl group at C-13, which was supported by the literature data [38]. The configuration of the C=C double bonds was determined as 9*Z* and 11*E* from the *J* coupling constants (*J*_H-9–H-10_ = 10.9 Hz, *J*_H-11–H-12_ = 15.2 Hz). The structure of **A1** was elucidated as the oxylipin (9*Z*,11*E*)-13-hydroxy-9,11-octadecadienoic acid (13-HODE), which were corroborated by comparison of NMR spectroscopic data with those reported in the literature [39]. 13-HODE has been isolated from different plant and lichen species, such as *Machilus salicina* [40], *Salix glandulosa* [41], *Urtica dioica* [42], and *Parmotrema hypoleucinum* [43], but this is the first report of its isolation from *A. altissima*.

(10*E*,12*Z*)-9-hydroxy-10,12-octadecadienoic acid (**A2**) was isolated as a white amorphous solid. The molecular formula of **A2** was identical to that of **A1** (C_18_H_32_O_3_) based on the sodium adduct peak at *m*/*z* 319.2243 [M+Na]^+^ and the deprotonated molecule peak at *m*/*z* 295.2277 [M–H]^−^ in the HESI-HRMS spectra (Figure 2f and Figures S11 and S12). The ^1^H and ^13^C NMR spectra of **A2** highly resembled those of **A1,** with only negligible chemical shift differences (max. Δ*δ*_H_ = 0.03 ppm, max. Δ*δ*_C_ = 0.3 ppm). On the other hand, MS/MS spectrum of **A2** (Figure 2g and Figure S13) exhibited a main fragment ion at *m*/*z* 171.1028 ([M–H–C_9_H_16_]^−^, C_9_H_15_O_3_^−^) corresponding to the alpha cleavage of the aliphatic chain with a hydroxyl group located at C-9, which was confirmed by the reported data [38]. The configuration of the C=C double bonds was determined as 10*E* and 12*Z* from the *J* coupling constants (*J*_H-10–H-11_ = 15.1 Hz, *J*_H-12–H-13_ = 10.9 Hz). Therefore, compound **A2** was assigned as the oxylipin (10*E*,12*Z*)-9-hydroxy-10,12-octadecadienoic acid (9-HODE), which was confirmed by comparison of its NMR spectroscopic data with those published [39]. 9-HODE has been found in various plants, including *Carthamus oxyacantha* [44], *Artemisia armeniaca* [45], *Discopodium penninervium* [46], also in fungi (e.g., *Penicillium ubiquetum* [47]), and in the green alga *Klebsormidium flaccidum* var. *zivo* [48]. However, this is the first report of its presence in tree of heaven.

Hexadecanedioic acid (**A3**) was obtained as a white amorphous solid with a molecular formula of C_16_H_30_O_4_ inferred from the sodium adduct peak at *m*/*z* 309.2036 [M+Na]^+^ and the deprotonated molecule peak at *m*/*z* 285.2070 [M–H]^−^ detected in the HESI-HRMS spectra (Figure 3d and Figure S20 and S21), indicating two DBEs. Its ^1^H NMR spectrum displayed only methylene signals at *δ*_H_ 2.27 (t, *J* = 7.4 Hz, 4H, H-2, H-15), 1.60 (p, *J* = 7.1 Hz, 4H, H-3, H-14), and 1.30 (br s, 20H, H-4–H-13). The ^13^C DEPTQ spectrum exhibited only 8 carbon resonances, suggesting a symmetric molecule, including one carboxylic carbon signal at *δ*_C_ 177.8 (C-1, C-16) and seven methylene signals at *δ*_C_ 35.0 (C-2, C-15), 30.7–30.3 (C-4–C-13), 26.1 (CH_2_, C-3, C-14). These NMR data demonstrated that compound **A3** was a long-chain dicarboxylic acid and it was identified as hexadecanedioic acid, also known as thapsic acid. Hexadecanedioic acid has been isolated from the stem cutin of *Pinus radiata* [49] and the juice of *Citrus pectinifera* [50], and it is also a metabolite of the yeast *Metschnikowia pulcherrima* [51]. However, this is the first description of **A3** in *A. altissima*.

16-Hydroxyhexadecanoic acid (**A4**) was isolated as a white amorphous solid. The molecular formula of compound **A4** was found to be C_16_H_32_O_3_ based on the sodium adduct peak at *m*/*z* 295.2243 [M+Na]^+^ and the deprotonated molecule peak at *m*/*z* 271.2278 [M–H]^−^ in the HESI-HRMS spectra (Figure 3c and Figure S28 and S29), requiring one DBE. The ^1^H NMR spectrum of **A4** revealed the presence of one oxygenated methylene at *δ*_H_ 3.54 (t, *J* = 6.7 Hz, 2H, H-16) and fifteen methylene groups. The ^13^C DEPTQ spectrum resolved only 13 carbon resonances including one carboxylic carbon at *δ*_C_ 178.0 (C-1), one oxygenated methylene carbon at *δ*_C_ 63.0 (C-16), and methylenes at *δ*_C_ 35.2 (C-2), 33.7 (C-15), 30.8–30.3 (C-4–C-13), 27.0 (C-14), 26.2 (C-3). These NMR spectroscopic data were consistent with a saturated fatty acid hydroxylated at the C-16. Consequently, compound **A4** was identified as 16-hydroxyhexadecanoic acid, also known as juniperic acid, which was verified by a good agreement between the experimental and the reported NMR data [52]. 16-Hydroxyhexadecanoic acid has mostly been found in the cutin of different plant species, such as *Citrus aurantifolia* [53], *Rosmarinus officinalis* [54], and *Hordeum vulgare* [55], but this is the first study identifying it in tree of heaven.

Alpinagalanate (**A5**) was obtained as a white amorphous solid and displayed the molecular formula C_26_H_40_O_6_ according to the deprotonated molecule peak observed at *m*/*z* 447.2752 [M–H]^−^ in the negative-mode HESI-HRMS spectrum (Figure S36), requiring 7 DBEs. The ^1^H NMR spectrum of **A5** demonstrated the presence of three aromatic protons at *δ*_H_ 7.07 (dd, *J* = 8.8, 2.6 Hz, 1H, H-6′), 7.03 (d, *J* = 1.9 Hz, 1H, H-2′), 6.92 (d, *J* = 8.2 Hz, 1H, H-5′) characteristic for a 1,2,4-trisubstituted aromatic ring, two vicinal, *trans*-oriented olefinic protons at *δ*_H_ 7.61 (d, *J* = 15.9 Hz, 1H, H-7′), 6.29 (d, *J* = 15.9 Hz, 1H, H-8′), an oxygenated methine proton at *δ*_H_ 4.19 (t, *J* = 6.7 Hz, 2H, H-1), a methoxy group at *δ*_H_ 3.93 (s, 3H, 3′–OCH_3_), and fourteen methylenes. The analysis of the ^13^C DEPTQ spectrum with the HSQC data revealed one carboxylic carbon at *δ*_C_ 176.1 (C-16); one ester carbonyl carbon at *δ*_C_ 167.6 (C-9′); six aromatic carbons at *δ*_C_ 148.0 (C-4′), 146.9 (C-3′), 127.2 (C-1′), 123.2 (C-6′), 114.8 (C-5′), and 109.4 (C-2′); two olefinic carbons at *δ*_C_ 144.8 (C-7′) and 115.8 (C-8′); one oxygenated methylene at *δ*_C_ 64.8 (C-1); one methoxy at *δ*_C_ 56.1 (5′–OCH_3_); and methylenes at *δ*_C_ 33.5 (C-15), 29.9–29.2 (C-4–C-13), 28.9 (C-2), 26.1 (C-3), and 24.9 (C-14). The connectivity between the aromatic ring and the C=C double bond was confirmed by long-range HMBC correlations H-8′/C-1′, H-7′/C-1′, H-7′/C-2′, and H-7′/C-6′. The presence of an *α*,*β*-unsaturated ester was evidenced by HMBC cross-peaks H-7′/C-9′ and H-8′/C-9′. The attachment of the methoxy group to the aromatic ring at C-3′ position was confirmed by the HMBC correlation 3′-OCH_3_/C-3′. The connection of the hydroxyl group at position C-4′ was deduced from the downfield shift at *δ*_C_ 148.0 of the aromatic carbon C-4′. These NMR data indicated that compound **A5** contained a ferulate moiety. The connection of the aliphatic side chain to the ester bond was supported by the HMBC correlation H-1/C-9′ and the presence of a terminal carboxylic moiety was established. Accordingly, compound **A5** was elucidated as the ferulate ester of 16-hydroxyhexadecanoic acid (**A4**), named alpinagalanate [56]. The measured NMR spectra showed good agreement with the literature data [56]. Alpinagalanate (16-feruloyloxypalmitic acid) was first isolated from the pollen of *Biota orientalis* [57] and later from the rhizome of *Alpinia galanga* from where its common name is derived [56]. This is the first study describing it in *A. altissima*.

Canthin-6-one (**A6**) was isolated as a yellow amorphous powder. The molecular formula of compound **A6** (C_14_H_8_N_2_O) was determined from the observed sodium adduct peak at *m*/*z* 243.0529 [M+Na]^+^ and the protonated molecule at *m*/*z* 221.0708 [M+H]^+^ in the positive-mode HESI-HRMS spectrum (Figure 4f and Figure S43). The ^1^H NMR spectrum of **A6** displayed two pairs of vicinal, aromatic protons at *δ*_H_ 8.83 (d, *J* = 5.0 Hz, 1H, H-2) and 7.97 (d, *J* = 5.0 Hz, 1H, H-1), and at *δ*_H_ 8.03 (d, *J* = 9.8 Hz, 1H, H-4) and 6.99 (d, *J* = 9.8 Hz, 1H, H-5), as well as four proton resonances characteristic for a 1,2-disubstituted aromatic ring at *δ*_H_ 8.68 (dt, *J* = 8.2, 0.9 Hz, 1H, H-8), 8.12 (ddd, *J* = 7.8, 1.2, 0.7 Hz, 1H, H-11), 7.71 (ddd, *J* = 8.3, 7.4, 1.2 Hz, 1H, H-9), and 7.54 (td, *J* = 7.6, 1.0 Hz, 1H, H-10). The ^13^C DEPTQ spectrum displayed 14 carbon signals, and in conjunction with the HSQC and HMBC spectra, allowed for the identification of a *β*-carboline alkaloid, canthin-6-one, which was corroborated by NMR spectroscopic literature data [58]. Canthin-6-one is a widely known alkaloid in many plant species, including the genus *Ailanthus*. It has been described as a component of *A. malabarica* wood [59], and *A. altissima* stem bark [22], root bark [60], and callus culture [61].

### 2.4. Confirmation of the Antibacterial Activity of Isolated Compounds

The antibacterial efficacy of the isolated compounds was assessed by an in vitro microdilution assay against the Gram-positive *B. subtilis* (Table 1). Based on the literature data, the linoleic acid-derived 13-HODE (**A1**) and 9-HODE (**A2**) showed antibacterial effect against Gram-positive *Bacillus subtilis*, *Micrococcus flavus*, and *Staphylococcus aureus* in agar plate diffusion tests, in which 13-HODE produced larger and 9-HODE smaller inhibition zones than that of linoleic acid [62]. The MIC value against *S. aureus* was determined as 75 µg/mL for 13-HODE and 100 µg/mL for both 9-HODE and linoleic acid [62] that are similar to those obtained in this study for hydroxylated fatty acids against *B. subtilis* (66.7 µg/mL). Both 13-HODE and 9-HODE displayed also cytotoxic effects against human tumor cell lines (K562, MCF-7, and HepG2 cells) [63] and 13-HODE was found to inhibit in vitro the activity of aromatase enzyme [42]. Among the isolated compounds, the strongest activity against *B. subtilis* was attributed to canthin-6-one (**A6**) with a MIC of 8.3 µg/mL, approximately 10 times higher than that of the positive control, gentamicin (0.8 µg/mL), and similar to the previously reported data in the literature (MIC = 100 µM corresponding to 22.0 µg/mL) [64]. Canthin-6-one exhibited comparable antibacterial activity against other Gram-positive strains, like *Bacillus cereus* (MIC = 7.81 µg/mL) [65], *Staphylococcus aureus* (MIC = 16 µg/mL) [66], and various *Mycobacterium* species, such as *M. fortuitum*, *M. smegmatis*, and *M. phlei* (MIC = 8–16 µg/mL) [67]. Gram-negative bacteria, *Klebsiella aerogenes* and *Escherichia coli*, fungal strain *Aspergillus niger*, and yeast *Candida albicans* were also inhibited by canthin-6-one [68]. No antibacterial activity has been yet assigned to hexadecanedioic acid (**A3**) that showed 54.8% inhibition against *B. subtilis* at the highest applied concentration (133.3 µg/mL) in this study. However, the antifungal activity of hexadecanedioic acid against the plant pathogen *Botrytis cinerea* [51] and its agonist effect on succinate receptor 1 [69] have been reported. For 16-hydroxyhexadecanoic acid (**A4**), only *α*-glucosidase inhibitory activity has been observed [70]. Thus, in this study, the antibacterial effect of 16-hydroxyhexadecanoic acid was first described with the identical MIC value as that of 13-HODE and 9-HODE (66.7 µg/mL). There is no literature data available on the bioactivity of alpinagalanate (16-feruloyloxypalmitic acid, **A5**), and as expected, it proved to be inactive against *B. subtilis* in this study.

## 3. Materials and Methods

### 3.1. Materials

Preparative silica gel (no. 60752, high-purity grade, 60 Å pore size, 230–400 mesh particle size) was obtained from Sigma-Aldrich (St. Louis, MO, USA). Methanol (LC-MS grade) was purchased from Merck (Darmstadt, Germany). Isopropyl acetate, molybdatophosphoric acid, gentamicin, *p*-anisaldehyde, chloroform-*d* (≥99.8 atom% D, contains 0.5 wt.% silver foil as stabilizer, and 0.03% (*V*/*V*) TMS), and methanol-*d*_4_ (≥99.8 atom% D) were purchased from Sigma-Aldrich (Budapest, Hungary). The acetic acid was from Lach-Ner (Neratovice, Czech Republic) and all other solvents of analytical grade were supplied by Molar Chemicals (Halásztelek, Hungary) or Reanal (Budapest, Hungary). Basic bismuth nitrate and potassium iodide were from Reanal. 3-(4,5-Dimethylthiazol-2-yl)-2,5-diphenyltetrazolium bromide (MTT) was acquired from Carl Roth (Karlsruhe, Germany). The Gram-positive soil bacterium, *Bacillus subtilis* (strain F1276) was a gift from József Farkas (Central Food Research Institute, Budapest, Hungary). Ultrapure water was prepared by a Millipore Direct-Q 3 UV Water Purification System (Merck).

### 3.2. Sample Origin and Preparation

The whole-trunk bark, separated inner and outer trunk bark, and stem bark of young branches (Figure 1) were collected at three Hungarian sites, namely Leányfalu (47°42′11″ N, 19°04′25″ E; 254 m a.s.l.), Harta (46°41′44″ N, 19°03′13″ E; 90 m a.s.l.), and Balatongyörök (46°45′35″ N, 17°20′16″ E; 114 m a.s.l.), between May 2022 and July 2023. The voucher specimens (Table 2) are available at the herbarium of the Plant Protection Institute, HUN-REN Centre for Agricultural Research, Budapest, Hungary. The samples were thoroughly cleaned, dried at room temperature for a week and then ground by a coffee grinder (Sencor SCG 2050RD, Říčany, Czech Republic). All extracts were prepared by using ultrasound-assisted extraction (15 min, an ultrasonic bath (Sonorex Super RK 106, Bandelin, Berlin, Germany). Each powdered sample (1.5 g) was extracted with methanol (7.5 mL) and analyzed by TLC after filtration (PVDF syringe filter, Lab-Ex, Budapest, Hungary). For preparative scale, 100 g of outer bark (B3) was extracted three times with 1.2 L of methanol, 100 g from each of the two inner bark samples (H16, H19) was extracted three times with 1.5 L of methanol per sample, while 225 g of stem bark (70 g of L1, 70 g of H3, and 85 g of H16) was extracted three times with 1.2 L of methanol. The extracts of each sample type (inner and outer trunk bark, and stem bark) were separately filtered (Whatman No. 2 filter paper, Sigma-Aldrich, Budapest, Hungary) and concentrated in vacuo by a rotary evaporator (Rotavapor R-134, Büchi, Flawil, Switzerland).

### 3.3. Thin-Layer Chromatography

Glass- and aluminum-backed TLC silica gel 60 F_254_ plates were purchased from Merck (Darmstadt, Germany). Plates were cut into smaller pieces with a blade or a smartCUT Plate Cutter (CAMAG). Extracts and fractions (10 µL) were applied onto the TLC layer by the Automatic TLC Sampler 3 (ATS3, CAMAG, Muttenz, Switzerland) or manually, using a 10 µL microsyringe (Hamilton, Bonaduz, Switzerland), as 5–7 mm bands with 8 mm distance from the lower plate edge. Separations were performed in a saturated (for 10 min) twin trough chamber (10 cm × 20 cm, CAMAG) with toluene–isopropyl acetate–methanol (5:4:1, *V*/*V*). Plates were developed up to 90 mm from the lower edge of the layer, which took approximately 20 min. After development and drying in a stream of cold air for 5 min, images of the plates were documented with a digital camera (Cybershot DSC-HX60, Sony, Neu-Isenberg, Germany) under a UV lamp (CAMAG) at 254 nm and 366 nm.

Post-chromatographic derivatization was performed by the following three reagents: *p*-anisaldehyde sulfuric acid reagent, molybdatophosphoric acid reagent and Dragendorff’s reagent. Derivatization was performed by immersing the plates in *p*-anisaldehyde sulfuric acid reagent (500 μL of *p*-anisaldehyde, 10 mL of acetic acid, 100 mL of methanol, and 5 mL of concentrated sulfuric acid (96%)), or molybdatophosphoric acid reagent (5 g of reagent dissolved in 200 mL of ethanol), followed by heating for 5 min at 110 °C or 150 °C, respectively (Advanced Hot Plate, VWR, Debrecen, Hungary). For the detection of alkaloids, the plates were immersed in Dragendorff’s reagent consisting of 5 mL of Solution I (1.7 g of basic bismuth nitrate in 20 mL of acetic acid and 80 mL of water), 5 mL of Solution II (40 g of potassium iodide in 100 mL of water), 5 mL of acetic acid, and 25 mL of water. Derivatization was followed by documentation at white light illumination (Vis, transmittance mode, 96891 Salobrena 2 LED lamp, Eglo Lux, Dunakeszi, Hungary).

### 3.4. TLC–DB

After TLC separation, described in Section 3.3., the detection of the antibacterial compounds in the extracts, fractions, and isolated compounds was performed following the general workflow for the *B. subtilis* bioassays and the preparation of the bacterial cell suspensions previously described in detail [71]. Briefly, the developed and dried chromatograms were immersed in the cell suspension for 8 s and incubated at 37 °C and 100% humidity in a moistened polypropylene box for 2 h. This was followed by visualizing the bioautograms by dipping them into an aqueous MTT solution (1 mg/mL), followed by an additional 0.5-h incubation. The living, metabolically active cells can reduce the yellow MTT to the purple MTT-formazan, therefore, the bright zones against a purple background indicate the presence of antibacterial compounds.

### 3.5. TLC–HRMS/MS and FIA–HRMS/MS

For TLC–HESI-HRMS/MS, the binary pump (Vanquish Flex VF-P10, Dionex Softron, Germering, Germany) guided the methanol at a flow rate of 0.2 mL/min through the oval elution head (4 mm × 2 mm) of the TLC-MS Interface (CAMAG) to the HESI-II probe installed at the hybrid quadrupole-orbitrap mass spectrometer (Orbitrap Exploris 120, Thermo Fisher Scientific, Bremen, Germany). Spray voltage was set to 3.4 kV in positive mode and −2.0 kV in negative mode, capillary temperature was maintained at 320 °C, and nitrogen was used as both sheath and auxiliary gas (10 and 5 arbitrary units, respectively), produced by a Genius XE 35 gas generator (Peak Scientific, Glasgow, UK). Full scan MS spectra were recorded in both negative and positive ionization modes in the range of *m*/*z* 100–1000 with a resolution of 120,000, a standard automatic gain control target, and a maximum injection time of 100 ms.

Isolated compounds (2 µg/mL in methanol) were directly injected (5 µL) by flow injection analysis (FIA) into the mentioned HRMS system using the same acquisition parameters. Tandem mass spectra were acquired in HCD fragmentation mode with a normalized collision energy of 25% or 30% and a quadrupole isolation window of *m*/*z* 0.7 for precursor ion selection. Instrument control, operation, and data processing were performed with Xcalibur 4.7.69 software (Thermo Fisher Scientific).

### 3.6. Fractionation and Isolation of Bioactive Compounds

Fractionation and isolation of bioactive compounds from the concentrated crude extracts of the stem bark of young branches, outer trunk bark, and inner trunk bark was performed by flash column chromatography (CombiFlash NextGen 300, Teledyne Isco, Lincoln, NE, USA). The following columns were used: silica gel columns (RediSep Bronze, 40–60 μm, 40 g; RediSep Silver, 40–60 μm, 12 g; RediSep Bronze, 40–60 μm, 4 g, Teledyne Isco) and FlashPure EcoFlex C18 column (50 μm,12 g; Büchi, Uster, Switzerland).

#### 3.6.1. Fractionation and Isolation of Bioactive Compounds from the Stem Bark of Young Branches

Concentrated crude extract was dried with preparative silica gel (30 g) and placed into a dry load column and used for normal-phase flash column chromatography. The fractionation was carried out on a silica gel column (RediSep Bronze, 40–60 μm, 40 g) using a gradient system of *n*-hexane and acetone (0–1 min, 0%; 1–16 min, 0–20%; 16–26 min, 20%; 26–36 min, 20–100%; 36–39 min, 100% acetone) with a 30 mL/min eluent flow rate providing fractions 35–40 (*t*_R_ = 18.8–22.0 min, 229 mg). The fractions were merged, dried, re-suspended in chloroform, and further fractionated on a silica gel column (RediSep Bronze, 40–60 μm, 24 g) using a gradient system of toluene and ethyl acetate (0–0.5 min, 0%; 0.5–5.5 min, 0–10%; 5.5–10.5 min, 10%; 10.5–20.5 min, 10–15%; 20.5–25.5 min, 15–30% ethyl acetate) with 20 mL/min eluent flow rate resulting in fractions 29–35 (*t*_R_ = 16.1–20.6 min, 45 mg) and 36–42 (*t*_R_ = 20.6–24.7 min, 47 mg). The combined fractions were re-suspended in chloroform and separately further purified on a silica gel column (RediSep Silver, 40–60 μm, 12 g) using a gradient system of *n*-hexane and acetone (0–10 min, 0–10% or 0–8%; 10–50 min, 10% or 8% acetone) with a 15 mL/min eluent flow rate to obtain compound **A1** (fractions 27–35, *t*_R_ = 16.8–20.1 min, 5.6 mg) and compound **A2** (fractions 52–59, *t*_R_ = 39.3–46.2 min, 4.3 mg), respectively.

#### 3.6.2. Fractionation and Isolation of Bioactive Compounds from Outer Trunk Bark

The outer trunk bark concentrated crude extract dried with preparative silica gel (15 g) was placed into a dry load column and subjected to flash chromatography, using a silica gel column (RediSep Bronze, 40–60 μm, 40 g). The first separation was performed using a gradient system of *n*-hexane and acetone (0–1 min, 0%; 1–16 min, 0–20%; 16–26 min, 20%; 26–36 min, 20–100%; 36–39 min, 100% acetone) with 30 mL/min eluent flow rate yielding fractions 34–37 (*t*_R_ = 18.1–20.6 min, 46.3 mg) and 38–43 (*t*_R_ = 20.6–24.4 min, 26.2 mg). Fractions 34–37 were pooled, dried, re-suspended in chloroform, and further fractionated on a silica gel column (RediSep Bronze, 40–60 μm, 4 g) using a gradient system of *n*-hexane containing 3% of acetic acid and ethyl acetate (0–0.5 min, 0%; 0.5–5.5 min, 0–10%; 5.5–13.5 min, 10%; 13.5–21.5 min, 10–100%; 21.5–23.5 min, 100% ethyl acetate) with 10 mL/min eluent flow rate giving fractions 34–37/8–10 (*t*_R_ = 4.5–6 min, 7.7 mg) and 34–37/15–19 (*t*_R_ = 9.0–12.0 min, 10.5 mg). Fractions 34–37/8–10 and 38–43 from the first fractionation were re-suspended in chloroform and further purified on a C_18_ column (FlashPure EcoFlex C18, 50 μm,12g) using a gradient system of water containing 0.1% formic acid and isopropyl alcohol (0–2 min, 0–40%; 2–11.4 min, 40–85%; 11.4–11.6 min, 85–90%; 11.6–15 min, 100% isopropyl alcohol) with 10 mL/min eluent flow rate to obtain compound **A3** (fractions 11–16, *t*_R_ =7.8–11.5 min, 6.5 mg). Fractions 34–37/15–19 from the second fractionation were combined, dried, re-suspended in chloroform and further purified on a silica gel column (RediSep Bronze, 40–60 μm, 4 g) using a gradient system of *n*-hexane containing 3% of acetic acid and acetone (0–1 min, 0%; 1–11 min, 0–10%; 11–21 min, 10%; 21–26 min, 10–100% isopropyl alcohol) with 10 mL/min eluent flow rate to provide compound **A4** (fractions 17–18, *t*_R_ = 9.8–11.4 min, 4.1 mg) and compound **A5** (fraction 20, *t*_R_ = 12.2–13.0 min, 0.8 mg).

#### 3.6.3. Inner Trunk Bark

The inner trunk bark concentrated crude extracts dried with preparative silica gel (30 g) were placed into a dry load column and subjected to flash chromatography using a silica gel column (RediSep Bronze, 40–60 μm, 40 g). The first fractionation was achieved by applying a gradient system of *n*-hexane and acetone (0–1 min, 0%; 1–16 min, 0–20%; 16–26 min, 20%; 26–33.7 min, 20–80%; 33.7–36 min, 80–100%; 36–42 min, 100% acetone) with 30 mL/min eluent flow rate furnishing fractions 31–36 (*t*_R_ = 17.5–21.2 min, 638.6 mg). The merged, dried fractions were re-suspended in chloroform and further fractionated on a silica gel column (RediSep Bronze, 40–60 μm, 40 g) using a gradient system of *n*-hexane and isopropyl alcohol (0–1 min, 0%; 1–6 min, 0–5%; 6–14 min, 5%; 14–17 min, 5–100%; 17–23 min, 100% isopropyl alcohol) with an eluent flow rate of 15 mL/min to obtain fraction **FIB** (fractions 14–15, *t*_R_ = 9.2–10.8 min, 65.5 mg) and compound **A6** (fraction 27, *t*_R_ = 18.3–19.2 min, 9.1 mg).

### 3.7. NMR Spectroscopy

Samples were dissolved in chloroform-*d* (CDCl_3_) or methanol-*d*_4_ (CD_3_OD), and were transferred to standard 5 mm NMR tubes for analyses. All NMR spectra were acquired on a Bruker AVANCE NEO 600 (^1^H: 600.18 MHz, ^13^C: 150.93 MHz; 14.1 T) spectrometer equipped with a 5 mm quadruple resonance cryoprobe (QCI 600S3 H&F-P/C/N-D-05 Z XT) (Bruker Corporation, Billerica, MA, USA) at 298 K. The instrument was operated and controlled by Bruker TopSpin 4.0.8 (Bruker Corporation) software. ^1^H and ^13^C chemical shifts are reported on the delta scale as parts per million (ppm) relative to the NMR solvent used (CHCl_3_ residual peak at *δ*_H_ = 7.26 ppm and CDCl_3_ at *δ*_C_ = 77.16 ppm; CHD_2_OD residual peak at *δ*_H_ = 3.31 ppm and CD_3_OD at *δ*_C_ = 49.00 ppm). Spin-spin coupling constants (*J*) are given in Hz. The signal multiplicities are denoted as s—singlet, br s—broad singlet, d—doublet, t—triplet, q—quartet, p—pentet, m—multiplet, dd—doublet of doublets, dt—doublet of triplets, td—triplet of doublets, tt—triplet of triplets, qd—quartet of doublets, ddd—doublet of doublet of doublets, ddt—doublet of doublet of triplets. The complete ^1^H and ^13^C resonance assignments were carried out using conventional one-dimensional ^1^H (*zg30*) and ^13^C DEPTQ (*deptqgpsp*) as well as two-dimensional homonuclear ^1^H–^1^H COSY (*cosygpmfppqf*) and ^1^H–^1^H TOCSY (*dipsi2gpphzs*, mixing time: 80 ms), and heteronuclear ^1^H–^13^C edHSQC (*hsqcedetgpsp.3*, ^1^*J*_C–H_ = 145 Hz) and ^1^H–^13^C HMBC (*hmbcetgpl3nd*, *^n^J*_C–H_ = 8 Hz) experiments. They were recorded with the specified pulse sequences included in the standard spectrometer software package. NMR data of each compound were compared with the literature data.

### 3.8. Bacillus subtilis Microplate Assay of the Isolated Compounds

The determination of the minimal inhibitory concentration (MIC) of the isolates against the *B. subtilis* microbial growth was performed using non-treated, flat-bottom 96-well microplates (VWR, Debrecen, Hungary). *B. subtilis* was grown in Lysogeny broth (10 g/L tryptone (Reanal, Budapest, Hungary), 5 g/L yeast extract (Scharlau), and 10 g/L sodium chloride (Reanal)) at 37 °C by shaking at 120 rpm. A two-fold ethanolic dilution series of 10 µL of the isolates (**A1**–**A6**, dissolved in ethanol, 2 mg/mL) and 5 µL of gentamicin (positive control, 0.1 mg/mL in water) was prepared in the microplates in triplicate. Ethanol was used as a negative control. Ethanol was evaporated from the wells in a sterile box, and 150 µL of bacterial suspension (10^5^ CFU/mL in Lysogeny broth) was added to each well. The final concentrations of **A1**–**A6** and gentamicin in the wells were in the range of 2.1–133.3 µg/mL and 0.1–3.3 µg/mL, respectively. The absorbance at 600 nm was measured by a spectrophotometer (Clariostar^®^ Plus microplate reader, BMG LABTECH, Ortenberg, Germany) immediately and after 24 h incubation at 37 °C by shaking at 900 rpm (PHMP Twin microplate shaker-incubator, Grant Inc., Beaver Falls, PA, USA). The experiment was repeated on two separate occasions.

## 4. Conclusions

In this study, the effect-directed screening and isolation of bioactive compounds of *A. altissima* bark extracts were performed utilizing TLC–*B. subtilis* bioassay. The stem bark of young branches, inner, and outer trunk bark contained different antibacterial compounds, with their amounts varying throughout the seasons. Six known antibacterial compounds were identified by HESI-HRMS/MS, and one- and two-dimensional NMR spectroscopy. 13-HODE (**A1**) and 9-HODE (**A2**), isolated from the stem bark, were also present in higher abundance in the inner bark. Hexadecanedioic acid (**A3**), 16-hydroxyhexadecanoic acid (**A4**), and alpinagalanate (**A5**) were found in the outer bark, while canthin-6-one (**A6**) was detected in the inner bark. The antibacterial activity of all isolates, except alpinagalanate (**A5**), was confirmed using a microdilution assay. To the best of our knowledge, this is the first study identifying 13-HODE, 9-HODE, hexadecanedioic acid, 16-hydroxyhexadecanoic acid, and alpinagalanate in tree of heaven, and also the first study reporting the antibacterial effect of hexadecanedioic acid (16-hydroxyhexadecanoic acid).

## Figures and Tables

**Figure 5:**
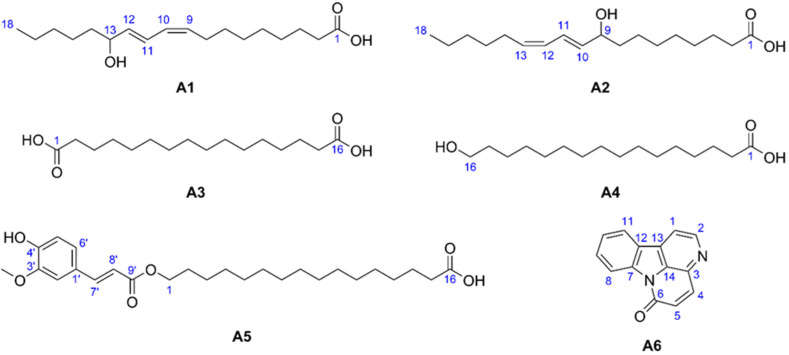
The chemical structures of isolated compounds (9*Z*,11*E*)-13-hydroxy-9,11-octadecadienoic acid (13-HODE, **A1**), (10*E*,12*Z*)-9-hydroxy-10,12-octadecadienoic acid (9-HODE, **A2**), hexadecanedioic acid (thapsic acid, **A3**), 16-hydroxyhexadecanoic acid (juniperic acid, **A4**), 16-feruloyloxypalmitic acid (alpinagalanate, **A5**), and canthin-6-one (**A6**) with the atomic numbering (blue).

**Table 1:** The minimal inhibitory concentration (MIC) values of isolated compounds (**A1**–**A6**) and positive control gentamicin in µg/mL against the *Bacillus subtilis* bacterial strain.

Isolate	Name	MIC (µg/mL)
A1	(9Z,11E)-13-hydroxy-9,11-octadecadienoic acid (13-HODE)	66.7
A2	(10*E*,12*Z*)-9-hydroxy-10,12-octadecadienoic acid (9-HODE)	66.7
A3	hexadecanedioic acid (thapsic acid)	>133.3 *
A4	16-hydroxyhexadecanoic acid (juniperic acid)	66.7
A5	alpinagalanate	>133.3
A6	canthin-6-one	8.3
	gentamicin	0.8

* 54.8% inhibition was observed at a concentration of 133.3 µg/mL.

**Table 2:** Collection time and area of the *Ailanthus altissima* samples.

Sample	Collection Time	Collection Area	Collected Tissue(s)	Voucher Code
H1	2 May 2022	Harta	stem bark	Aa.H1.8
H2	16 May 2022	Harta	stem bark outer trunk bark inner trunk bark	Aa.H2.8 Aa.H2.9OB Aa.H2.9IB
H3	30 May 2022	Harta	stem bark	Aa.H3.8
H4	3 July 2022	Harta	stem bark outer trunk bark inner trunk bark	Aa.H4.8 Aa.H4.9OB Aa.H4.9IB
H5	13 August 2022	Harta	outer trunk bark inner trunk bark	Aa.H5.9OB Aa.H5.9IB
H7	24 October 2022	Harta	outer trunk bark inner trunk bark	Aa.H7.9OB Aa.H7.9IB
H11	10 April 2023	Harta	outer trunk bark inner trunk bark	Aa.H11.9OB Aa.H11.9IB
H16	20 May 2023	Harta	stem bark inner trunk bark	Aa.H16.8 Aa.H16.9IB
H19	31 July 2023	Harta	inner trunk bark	Aa.H19.9IB
B3	26 July 2022	Balatongyörök	outer trunk bark	Aa.B3.9OB
L1	21 May 2022	Leányfalu	stem bark	Aa.L1.8

## Data Availability

Data are available upon reasonable request.

## Supplementary Materials

The following supporting information can be downloaded at: https://www.mdpi.com/article/10.3390/molecules29245846/s1, Figure S1: TLC–*B. subtilis* bioautogram of the flash chromatography fractions; Figure S2–S4, S11–S13, S20, S21, S28, S29, S36, and S43: HESI-HRMS(/MS) spectra of **A1**–**A6**; Figures S5–S10, S14–S19, S22–S27, S30–S35, S37–S42, and S44–S49: 1D and 2D NMR spectra of compounds **A1**–**A6**.
