# Supplementary material for: Bioassay-Guided Isolation and Identification of Antibacterial Compounds from Invasive Tree of Heaven Stem and Trunk Bark
Source: Molecules. 2024 Dec 11;29(24):5846. doi: 10.3390/molecules29245846 (PMC11680021; doi:10.3390/molecules29245846)
Supplement: Supplementary file 1 [file molecules-29-05846-s001.zip › molecules-3360194-supplementary.pdf]

Supplementary Data

Bioassay-Guided Isolation and Identification of Antibacterial Compounds from Invasive Tree of Heaven Stem and Trunk Bark

Anna Cselőtey [1], Márton Baglyas [1,2], Nóra Király [1], Péter G. Ott [1], Vesna Glavnik [3], Irena Vovk [3], Ágnes M. Móricz [1,*]

[1] Plant Protection Institute, HUN-REN Centre for Agricultural Research, Fehérvári út 132–144, 1116 Budapest, Hungary

[2] Doctoral School, Semmelweis University, Üllői út 26, 1085 Budapest, Hungary

[3] Laboratory for Food Chemistry, National Institute of Chemistry, Hajdrihova 19, 1000 Ljubljana, Slovenia

* Correspondence: moricz.agnes@atk.hun-ren.hu

Table of contents

No.	Legend	Page
**Figure S1.**	TLC–*B. subtilis* bioautogram of the flash chromatography fractions (Fr. 8-19) of the OB/34-37 fractions developed with toluene – isopropyl acetate – methanol 5:4:1 *V/V*.	S-5
**Figure S2.**	HESI$^+$-HRMS spectrum of (9*Z*,11*E*)-13-hydroxy-9,11-octadecadienoic acid (**A1**), *m/z* 319.2243 [M+Na]$^+$ (calculated for $C_{18}H_{32}O_3Na^+$, *m/z* 319.2244 [M+Na]$^+$, error: –0.2 ppm).	S-6
**Figure S3.**	HESI$^-$-HRMS spectrum of (9*Z*,11*E*)-13-hydroxy-9,11-octadecadienoic acid (**A1**), *m/z* 295.2277 [M–H]$^-$ (calculated for $C_{18}H_{31}O_3^-$, *m/z* 295.2279 [M–H]$^-$, error: –0.6 ppm).	S-7
**Figure S4.**	HESI$^-$-HRMS/MS spectrum of (9*Z*,11*E*)-13-hydroxy-9,11-octadecadienoic acid (**A1**) with a normalized HCD collision energy of 25%. Precursor ion: *m/z* 295.2280 [M–H]$^-$.	S-8
**Figure S5.**	^{1}H NMR spectrum of (9*Z*,11*E*)-13-hydroxy-9,11-octadecadienoic acid (**A1**) ($CDCl_3$, 600 MHz).	S-9
**Figure S6.**	^{13}C DEPTQ NMR spectrum of (9*Z*,11*E*)-13-hydroxy-9,11-octadecadienoic acid (**A1**) ($CDCl_3$, 151 MHz).	S-10
**Figure S7.**	^{1}H–^{1}H COSY NMR spectrum of (9*Z*,11*E*)-13-hydroxy-9,11-octadecadienoic acid (**A1**) ($CDCl_3$, 600 MHz).	S-11
**Figure S8.**	^{1}H–^{13}C edHSQC NMR spectrum of (9*Z*,11*E*)-13-hydroxy-9,11-octadecadienoic acid (**A1**) ($CDCl_3$, 600 and 151 MHz).	S-12
**Figure S9.**	^{1}H–^{13}C HMBC NMR spectrum of (9*Z*,11*E*)-13-hydroxy-9,11-octadecadienoic acid (**A1**) ($CDCl_3$, 600 and 151 MHz).	S-13
**Figure S10.**	^{1}H–^{1}H TOCSY NMR spectrum of (9*Z*,11*E*)-13-hydroxy-9,11-octadecadienoic acid (**A1**) ($CDCl_3$, 600 MHz).	S-14
**Figure S11.**	HESI$^+$-HRMS spectrum of (10*E*,12*Z*)-9-hydroxy-10,12-octadecadienoic acid (**A2**), *m/z* 319.2243 [M+Na]$^+$ (calculated for $C_{18}H_{32}O_3Na^+$, *m/z* 319.2244 [M+Na]$^+$, error: –0.2 ppm).	S-15
**Figure S12.**	HESI$^-$-HRMS spectrum of (10*E*,12*Z*)-9-hydroxy-10,12-octadecadienoic acid (**A2**), *m/z* 295.2277 [M–H]$^-$ (calculated for $C_{18}H_{31}O_3^-$, *m/z* 295.2279 [M–H]$^-$, error: –0.6 ppm).	S-16
**Figure S13.**	HESI$^-$-HRMS/MS spectrum of (10*E*,12*Z*)-9-hydroxy-10,12-octadecadienoic acid (**A2**) with a normalized HCD collision energy of 25%. Precursor ion: *m/z* 295.2281 [M–H]$^-$.	S-17
**Figure S14.**	^{1}H NMR spectrum of (10*E*,12*Z*)-9-hydroxy-10,12-octadecadienoic acid (**A2**) ($CDCl_3$, 600 MHz).	S-18
**Figure S15.**	^{13}C DEPTQ NMR spectrum of (10*E*,12*Z*)-9-hydroxy-10,12-octadecadienoic acid (**A2**) ($CDCl_3$, 151 MHz).	S-19
**Figure S16.**	^{1}H–^{1}H COSY NMR spectrum of (10*E*,12*Z*)-9-hydroxy-10,12-octadecadienoic acid (**A2**) ($CDCl_3$, 600 MHz).	S-20
**Figure S17.**	^{1}H–^{13}C edHSQC NMR spectrum of (10*E*,12*Z*)-9-hydroxy-10,12-octadecadienoic acid (**A2**) ($CDCl_3$, 600 and 151 MHz).	S-21
**Figure S18.**	^{1}H–^{13}C HMBC NMR spectrum of (10*E*,12*Z*)-9-hydroxy-10,12-octadecadienoic acid (**A2**) ($CDCl_3$, 600 and 151 MHz).	S-22
**Figure S19.**	^{1}H–^{1}H TOCSY NMR spectrum of (10*E*,12*Z*)-9-hydroxy-10,12-octadecadienoic acid (**A2**) ($CDCl_3$, 600 MHz).	S-23
**Figure S20.**	HESI$^+$-HRMS spectrum of hexadecanedioic acid (**A3**), *m/z* 309.2036 [M+Na]$^+$ (calculated for $C_{16}H_{30}O_4Na^+$, *m/z* 309.2036 [M+Na]$^+$, error: –0.1 ppm).	S-24
**Figure S21.**	HESI$^-$-HRMS spectrum of hexadecanedioic acid (**A3**), *m/z* 285.2070 [M–H]$^-$ (calculated for $C_{16}H_{29}O_4^-$, *m/z* 285.2071 [M–H]$^-$, error: –0.5 ppm).	S-25
**Figure S22.**	^{1}H NMR spectrum of hexadecanedioic acid (**A3**) (CD_3OD, 600 MHz).	S-26
**Figure S23.**	^{13}C DEPTQ NMR spectrum of hexadecanedioic acid (**A3**) (CD_3OD, 151 MHz).	S-27
**Figure S24.**	^{1}H–^{1}H COSY NMR spectrum of hexadecanedioic acid (**A3**) (CD_3OD, 600 MHz).	S-28

**Figure S25.**	^1H–^{13}C edHSQC NMR spectrum of hexadecanedioic acid (**A3**) (CD_3OD, 600 and 151 MHz).	S-29
**Figure S26.**	^1H–^{13}C HMBC NMR spectrum of hexadecanedioic acid (**A3**) (CD_3OD, 600 and 151 MHz).	S-30
**Figure S27.**	^1H–^1H TOCSY NMR spectrum of hexadecanedioic acid (**A3**) (CD_3OD, 600 MHz).	S-31
**Figure S28.**	HESI$^+$-HRMS spectrum of 16-hydroxyhexadecanoic acid (**A4**), *m/z* 295.2243 $[M+Na]^+$ (calculated for $C_{16}H_{32}O_3Na^+$, *m/z* 295.2244 $[M+Na]^+$, error: –0.2 ppm).	S-32
**Figure S29.**	HESI$^-$-HRMS spectrum of 16-hydroxyhexadecanoic acid (**A4**), *m/z* 271.2278 $[M–H]^–$ (calculated for $C_{16}H_{31}O_3^–$, *m/z* 271.2279 $[M–H]^–$, error: –0.3 ppm).	S-33
**Figure S30.**	^1H NMR spectrum of 16-hydroxyhexadecanoic acid (**A4**) (CD_3OD, 600 MHz).	S-34
**Figure S31.**	^{13}C DEPTQ NMR spectrum of 16-hydroxyhexadecanoic acid (**A4**) (CD_3OD, 151 MHz).	S-35
**Figure S32.**	^1H–^1H COSY NMR spectrum of 16-hydroxyhexadecanoic acid (**A4**) (CD_3OD, 600 MHz).	S-36
**Figure S33.**	^1H–^{13}C edHSQC NMR spectrum of 16-hydroxyhexadecanoic acid (**A4**) (CD_3OD, 600 and 151 MHz).	S-37
**Figure S34.**	^1H–^{13}C HMBC NMR spectrum of 16-hydroxyhexadecanoic acid (**A4**) (CD_3OD, 600 and 151 MHz).	S-38
**Figure S35.**	^1H–^1H TOCSY NMR spectrum of 16-hydroxyhexadecanoic acid (**A4**) (CD_3OD, 600 MHz).	S-39
**Figure S36.**	HESI$^-$-HRMS spectrum of alpinagalanate (**A5**), *m/z* 447.2749 $[M–H]^–$ (calculated for $C_{26}H_{39}O_6^–$, *m/z* 447.2752 $[M–H]^–$, error: –0.7 ppm).	S-40
**Figure S37.**	^1H NMR spectrum of alpinagalanate (**A5**) ($CDCl_3$, 600 MHz).	S-41
**Figure S38.**	^{13}C DEPTQ NMR spectrum of alpinagalanate (**A5**) ($CDCl_3$, 151 MHz).	S-42
**Figure S39.**	^1H–^1H COSY NMR spectrum of alpinagalanate (**A5**) ($CDCl_3$, 600 MHz).	S-43
**Figure S40.**	^1H–^{13}C edHSQC NMR spectrum of alpinagalanate (**A5**) ($CDCl_3$, 600 and 151 MHz).	S-44
**Figure S41.**	^1H–^{13}C HMBC NMR spectrum of alpinagalanate (**A5**) ($CDCl_3$, 600 and 151 MHz).	S-45
**Figure S42.**	^1H–^1H TOCSY NMR spectrum of alpinagalanate (**A5**) ($CDCl_3$, 600 MHz).	S-46
**Figure S43.**	HESI$^+$-HRMS spectrum of canthin-6-one (**A6**), *m/z* 243.0528 $[M+Na]^+$ (calculated for $C_{14}H_8N_2ONa^+$, *m/z* 243.0529 $[M+Na]^+$, error: –0.3 ppm); *m/z* 221.0708 $[M+H]^+$ (calculated for $C_{14}H_9N_2O^+$, *m/z* 221.0709 $[M+H]^+$, error: –0.6 ppm).	S-47
**Figure S44.**	^1H NMR spectrum of canthin-6-one (**A6**) ($CDCl_3$, 600 MHz).	S-48
**Figure S45.**	^{13}C DEPTQ NMR spectrum of canthin-6-one (**A6**) ($CDCl_3$, 151 MHz).	S-49
**Figure S46.**	^1H–^1H COSY NMR spectrum of canthin-6-one (**A6**) ($CDCl_3$, 600 MHz).	S-50
**Figure S47.**	^1H–^{13}C edHSQC NMR spectrum of canthin-6-one (**A6**) ($CDCl_3$, 600 and 151 MHz).	S-51
**Figure S48.**	^1H–^{13}C HMBC NMR spectrum of canthin-6-one (**A6**) ($CDCl_3$, 600 and 151 MHz).	S-52
**Figure S49.**	^1H–^1H TOCSY NMR spectrum of canthin-6-one (**A6**) ($CDCl_3$, 600 MHz).	S-53

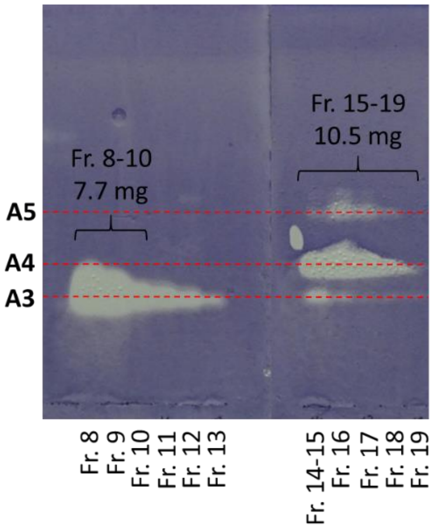

Figure S1. TLC–*B. subtilis* bioautogram of the flash chromatography fractions (Fr. 8-19) of the OB/34-37 fractions developed with toluene – isopropyl acetate – methanol 5:4:1 *V/V*.

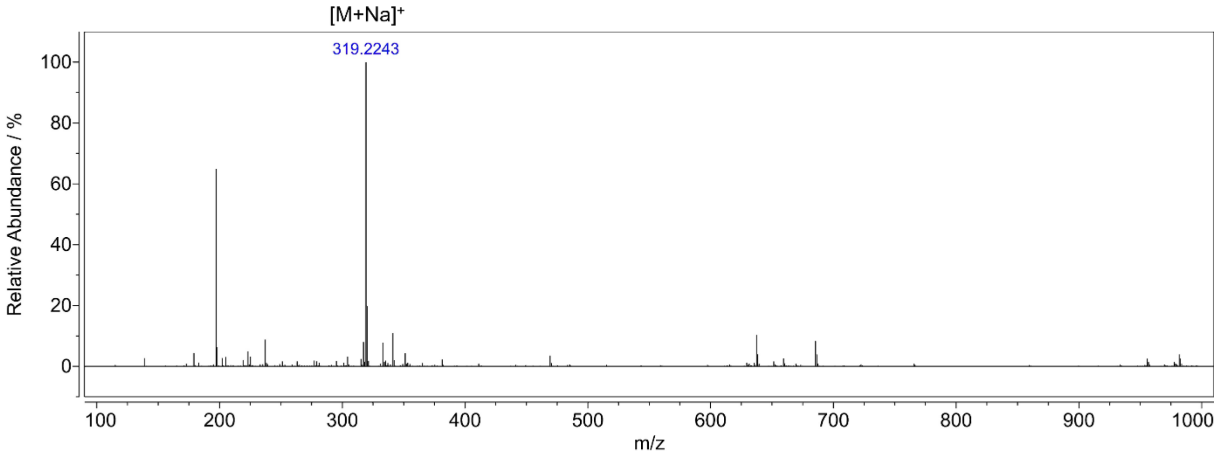

Figure S2. HR-ESI$^+$-MS spectrum of (9*Z*,11*E*)-13-hydroxy-9,11-octadecadienoic acid (**A1**), *m*/*z* 319.2243 $[M+Na]^+$ (calculated for $C_{18}H_{32}O_3Na^+$, *m*/*z* 319.2244 $[M+Na]^+$, error: –0.2 ppm).

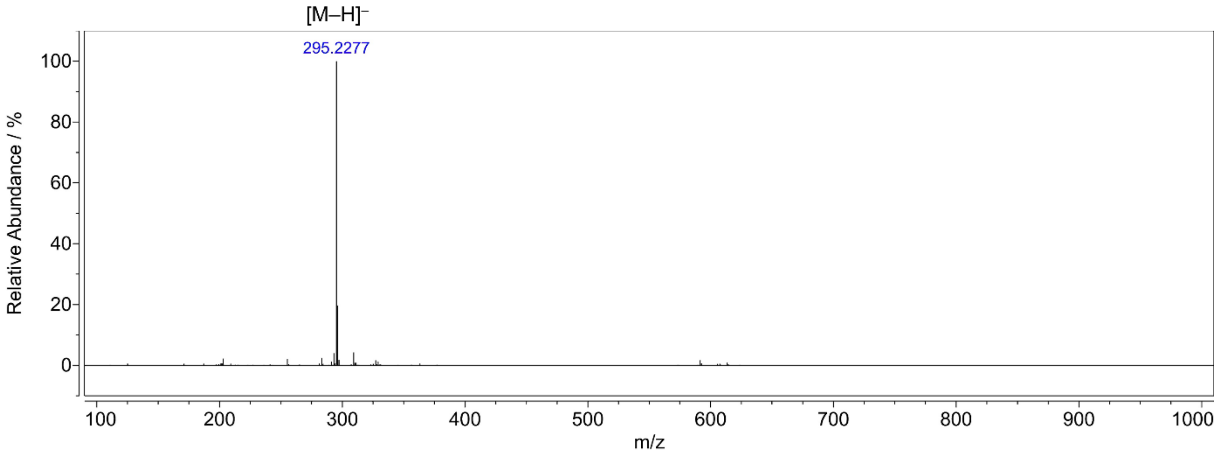

Figure S3. HR-ESI⁻-MS spectrum of (9*Z*,11*E*)-13-hydroxy-9,11-octadecadienoic acid (**A1**), *m*/*z* 295.2277 [M–H]⁻ (calculated for $C_{18}H_{31}O_3^-$, *m*/*z* 295.2279 [M–H]⁻, error: –0.6 ppm).

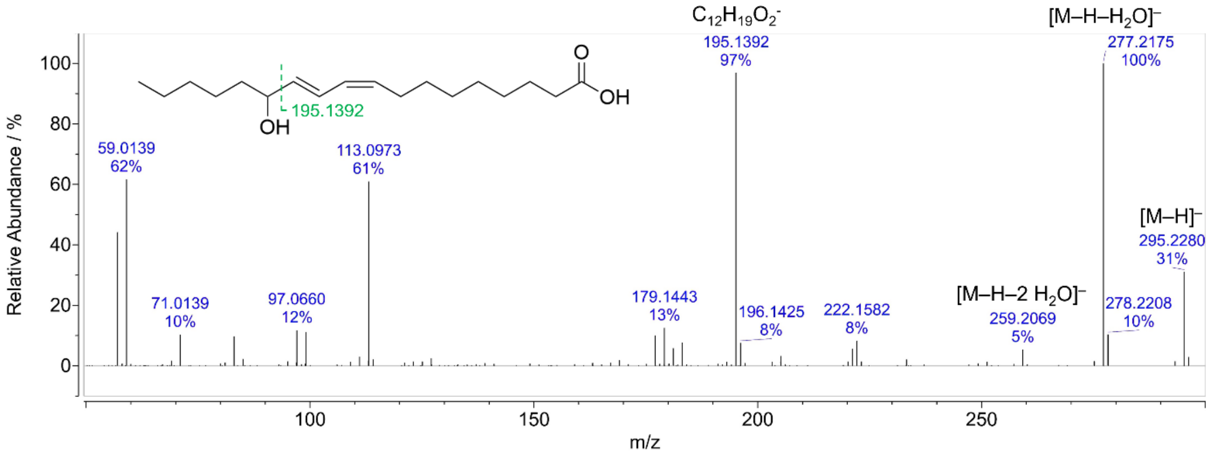

Figure S4. HR-ESI⁻-MS/MS spectrum of (9*Z*,11*E*)-13-hydroxy-9,11-octadecadienoic acid (**A1**) with a normalized HCD collision energy of 25%. Precursor ion: *m*/*z* 295.2280 [M–H]⁻.

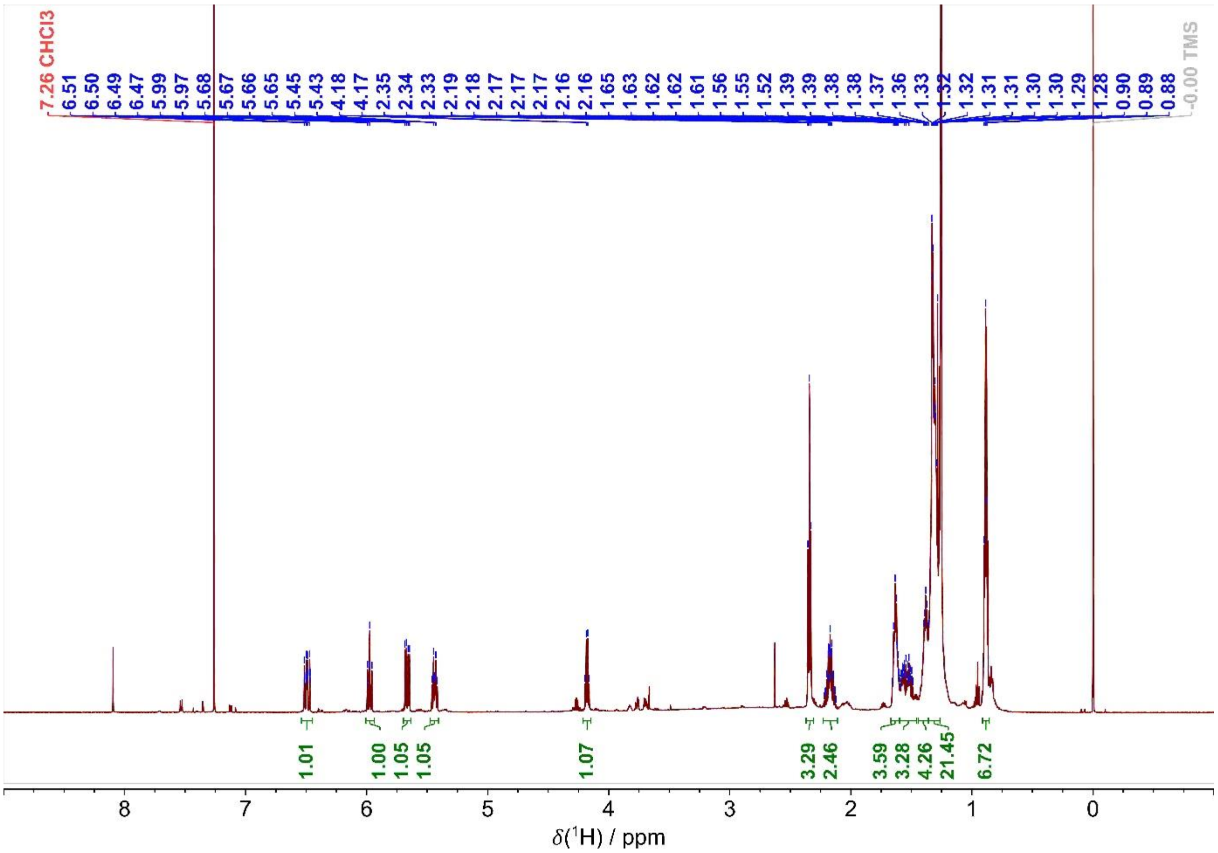

Figure S5. ^{1}H NMR spectrum of (9*Z*,11*E*)-13-hydroxy-9,11-octadecadienoic acid (**A1**) ($CDCl_3$, 600 MHz).

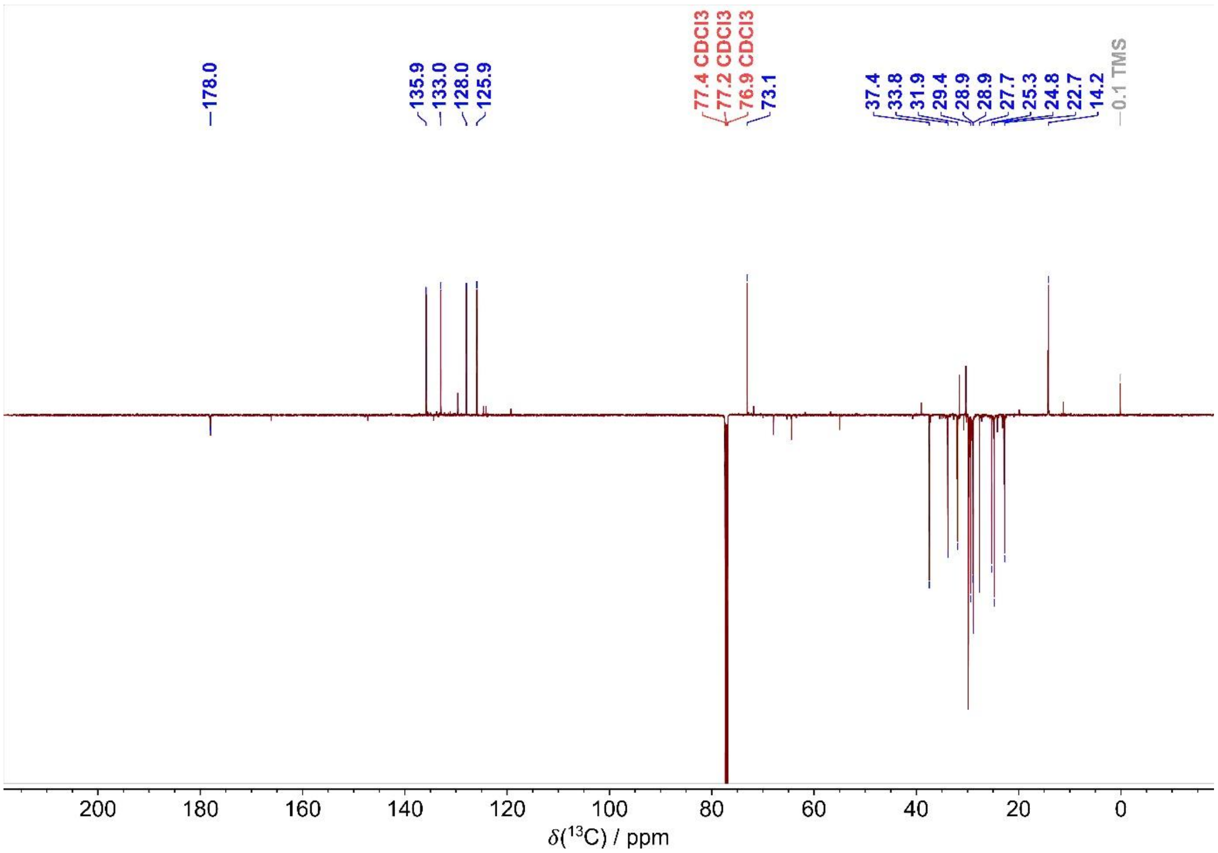

Figure S6. ^{13}C DEPTQ NMR spectrum of (9*Z*,11*E*)-13-hydroxy-9,11-octadecadienoic acid (**A1**) ($CDCl_3$, 151 MHz).

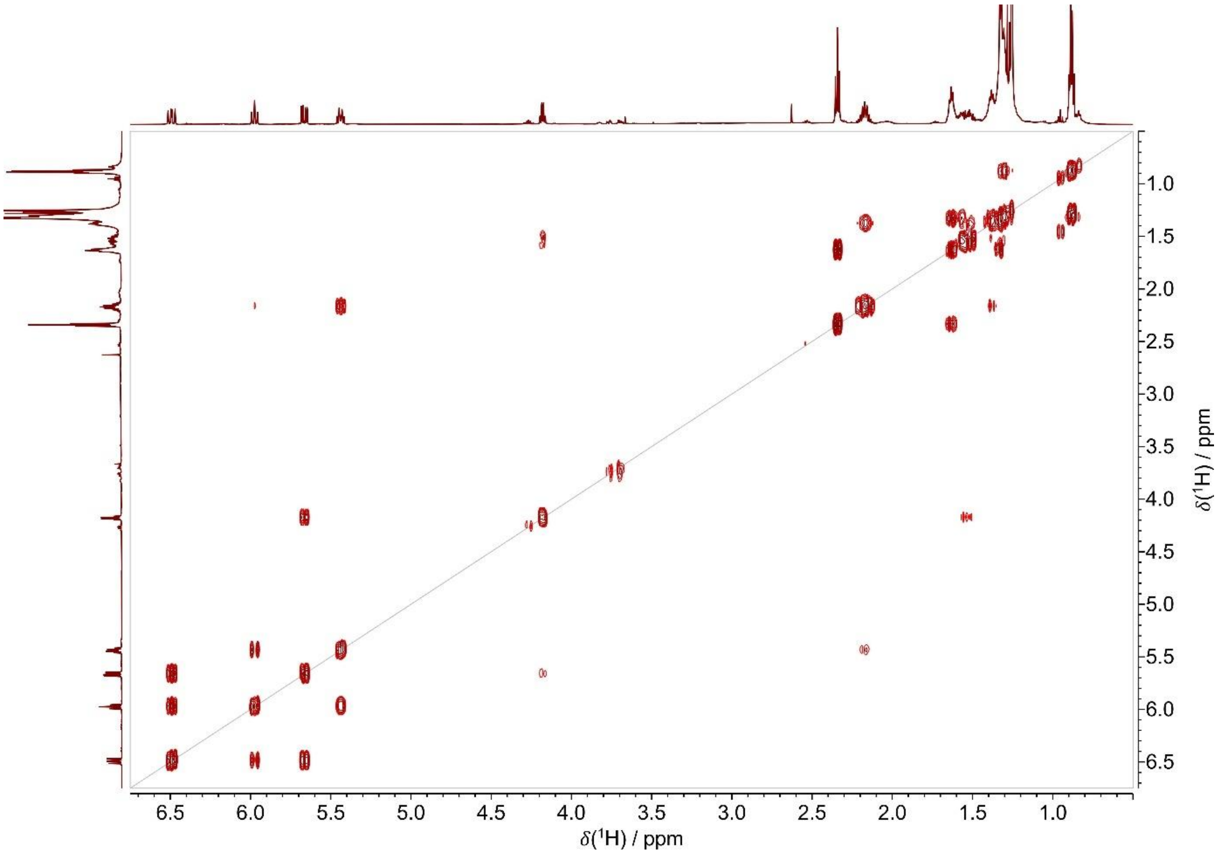

Figure S7. ^{1}H–^{1}H COSY NMR spectrum of (9*Z*,11*E*)-13-hydroxy-9,11-octadecadienoic acid (**A1**) ($CDCl_3$, 600 MHz).

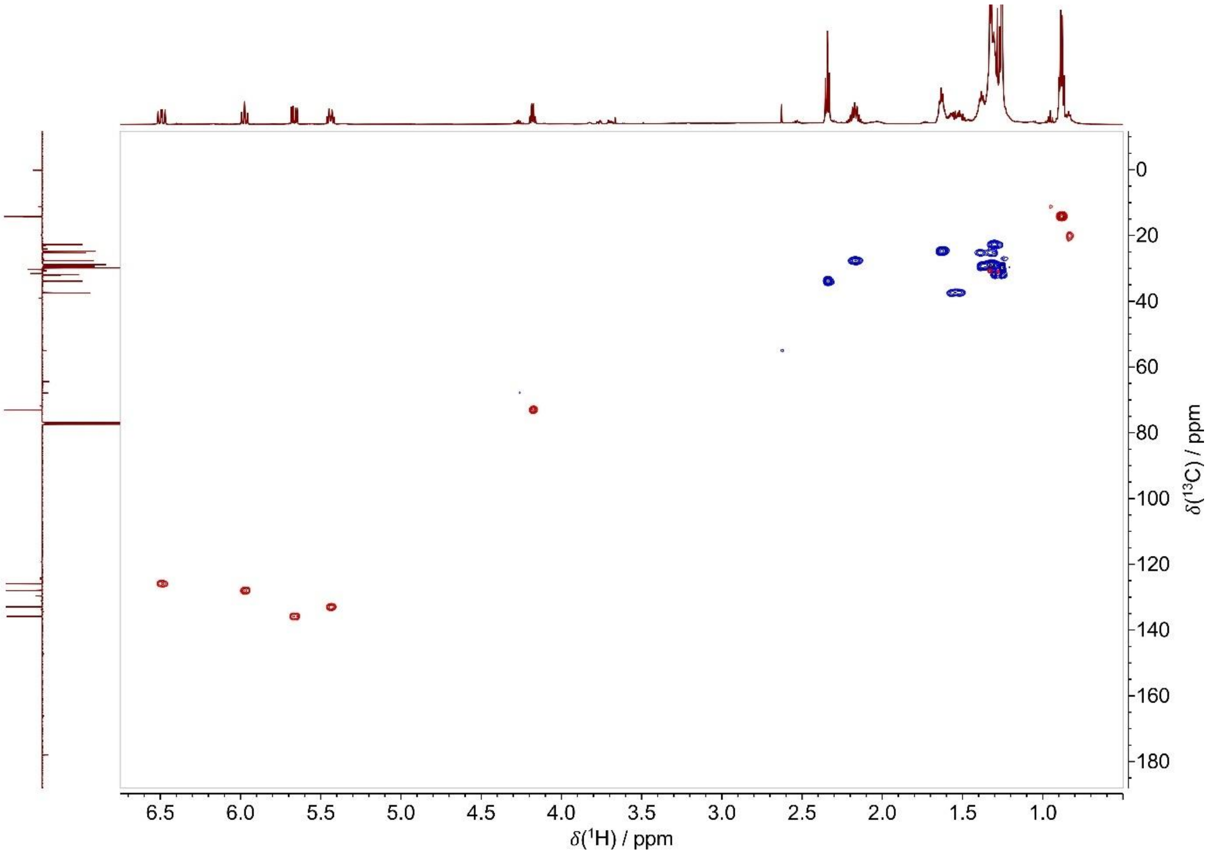

Figure S8. ^{1}H–^{13}C edHSQC NMR spectrum of (9*Z*,11*E*)-13-hydroxy-9,11-octadecadienoic acid (**A1**) ($CDCl_3$, 600 and 151 MHz).

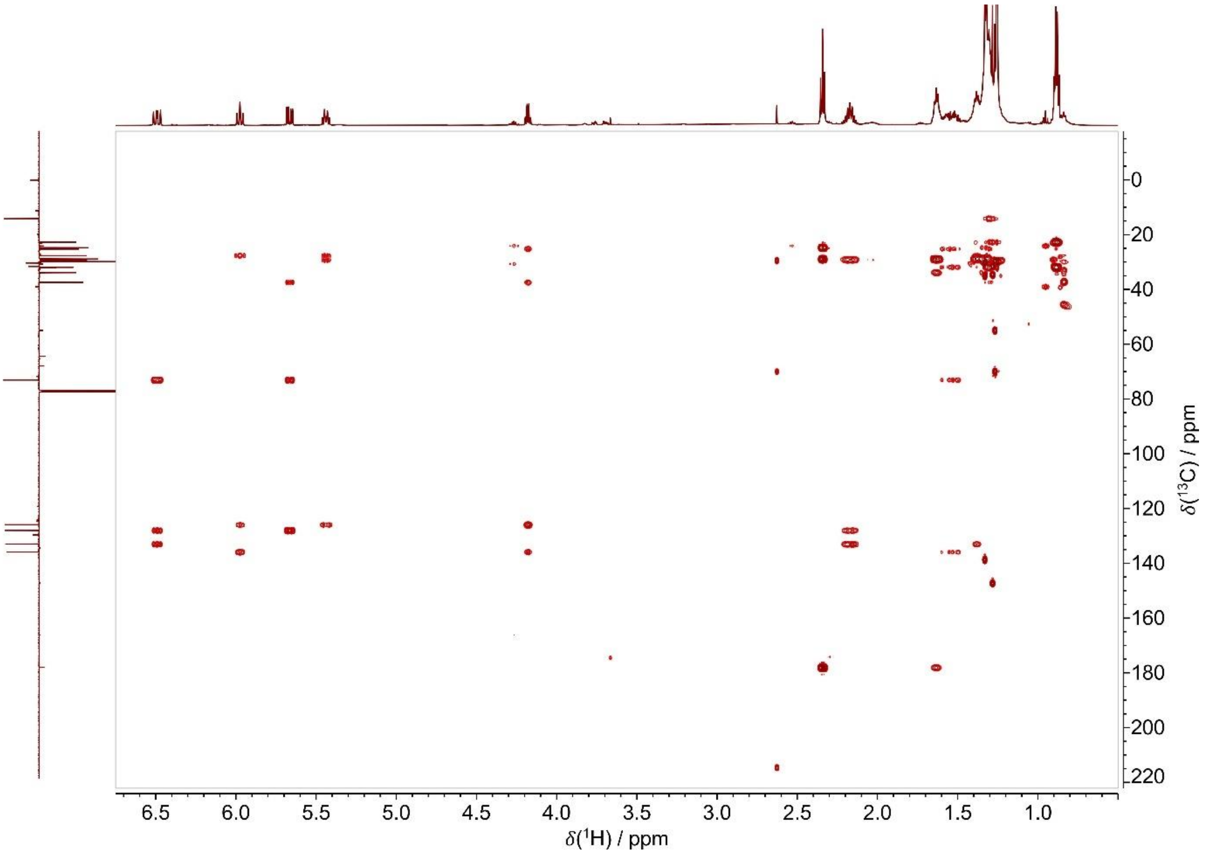

Figure S9. ^{1}H–^{13}C HMBC NMR spectrum of (9*Z*,11*E*)-13-hydroxy-9,11-octadecadienoic acid (**A1**) ($CDCl_3$, 600 and 151 MHz).

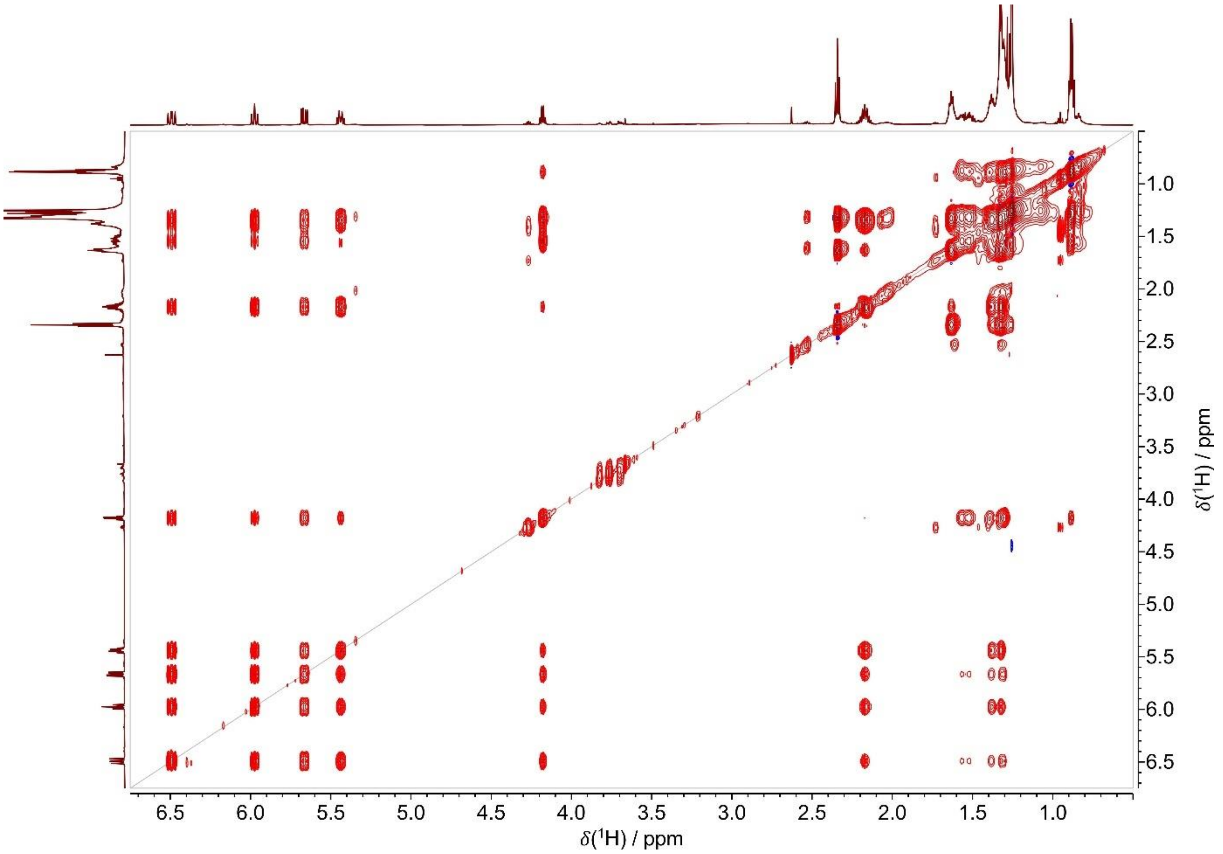

Figure S10. ^{1}H–^{1}H TOCSY NMR spectrum of (9*Z*,11*E*)-13-hydroxy-9,11-octadecadienoic acid (**A1**) ($CDCl_3$, 600 MHz).

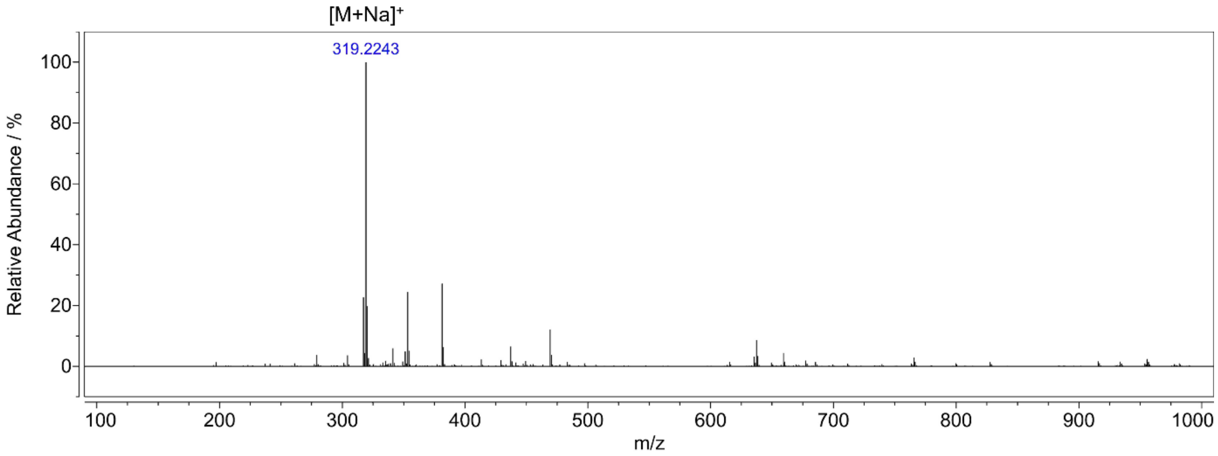

Figure S11. HR-ESI$^+$-MS spectrum of (10*E*,12*Z*)-9-hydroxy-10,12-octadecadienoic acid (**A2**), *m*/*z* 319.2243 [M+Na]$^+$ (calculated for $C_{18}H_{32}O_3Na^+$, *m*/*z* 319.2244 [M+Na]$^+$, error: –0.2 ppm).

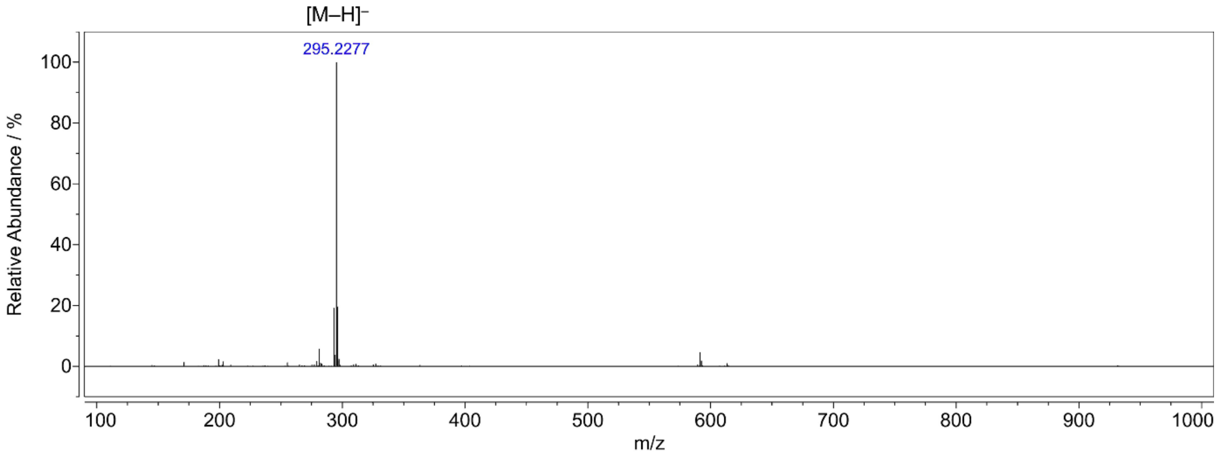

Figure S12. HR-ESI$^-$-MS spectrum of (10*E*,12*Z*)-9-hydroxy-10,12-octadecadienoic acid (**A2**), *m*/*z* 295.2277 [M–H]$^-$ (calculated for $C_{18}H_{31}O_3^-$, *m*/*z* 295.2279 [M–H]$^-$, error: –0.6 ppm).

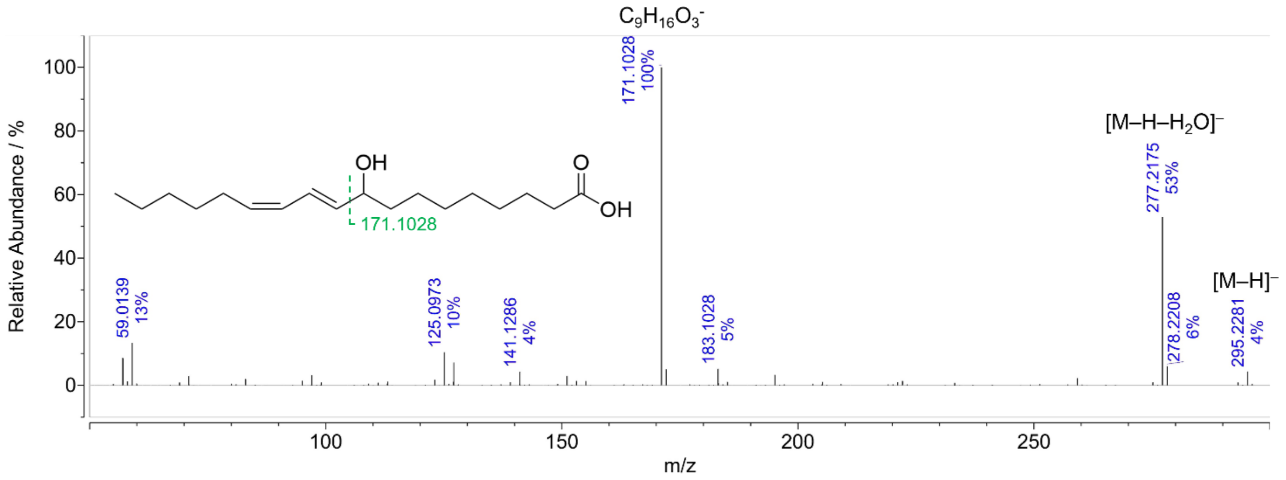

Figure S13. HR-ESI$^-$-MS/MS spectrum of (10*E*,12*Z*)-9-hydroxy-10,12-octadecadienoic acid (**A2**) with a normalized HCD collision energy of 25%. Precursor ion: *m*/*z* 295.2281 [M–H]$^-$.

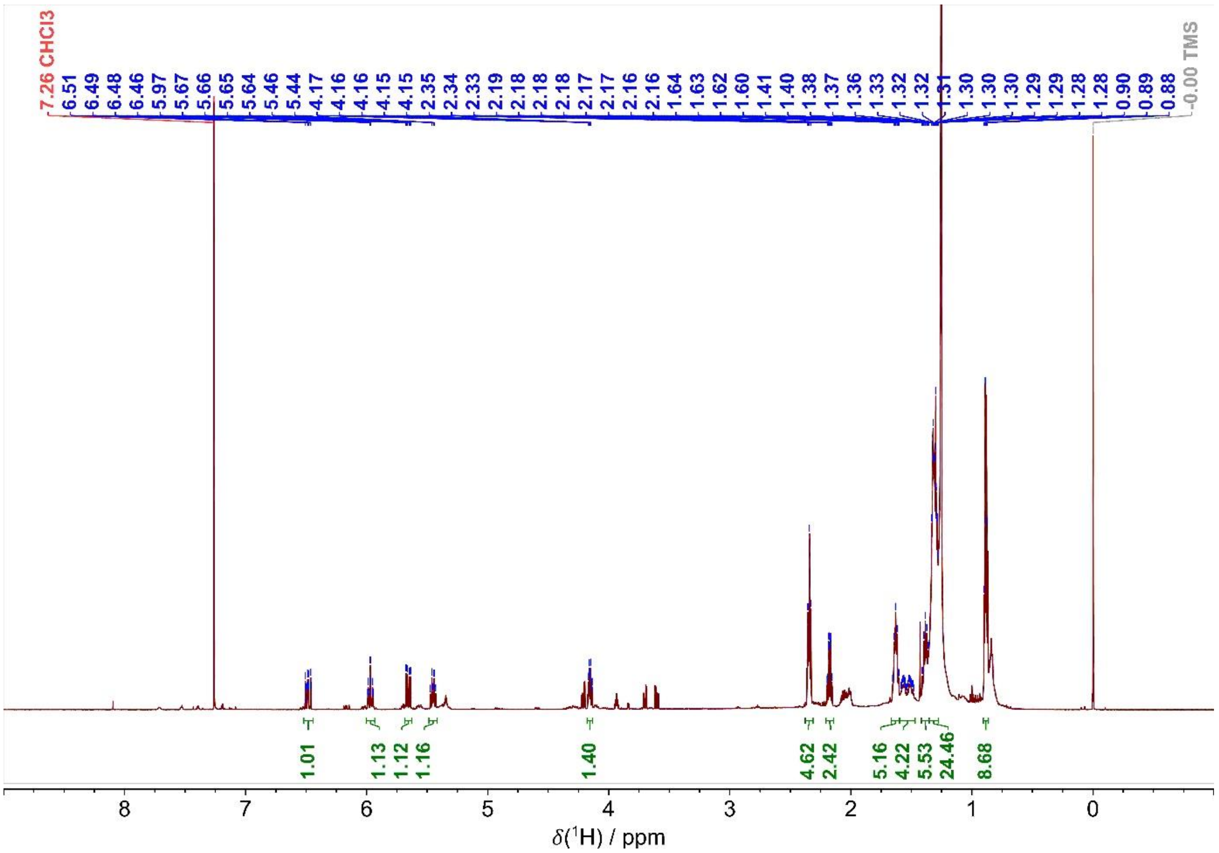

Figure S14. ^{1}H NMR spectrum of (10*E*,12*Z*)-9-hydroxy-10,12-octadecadienoic acid (**A2**) ($CDCl_3$, 600 MHz).

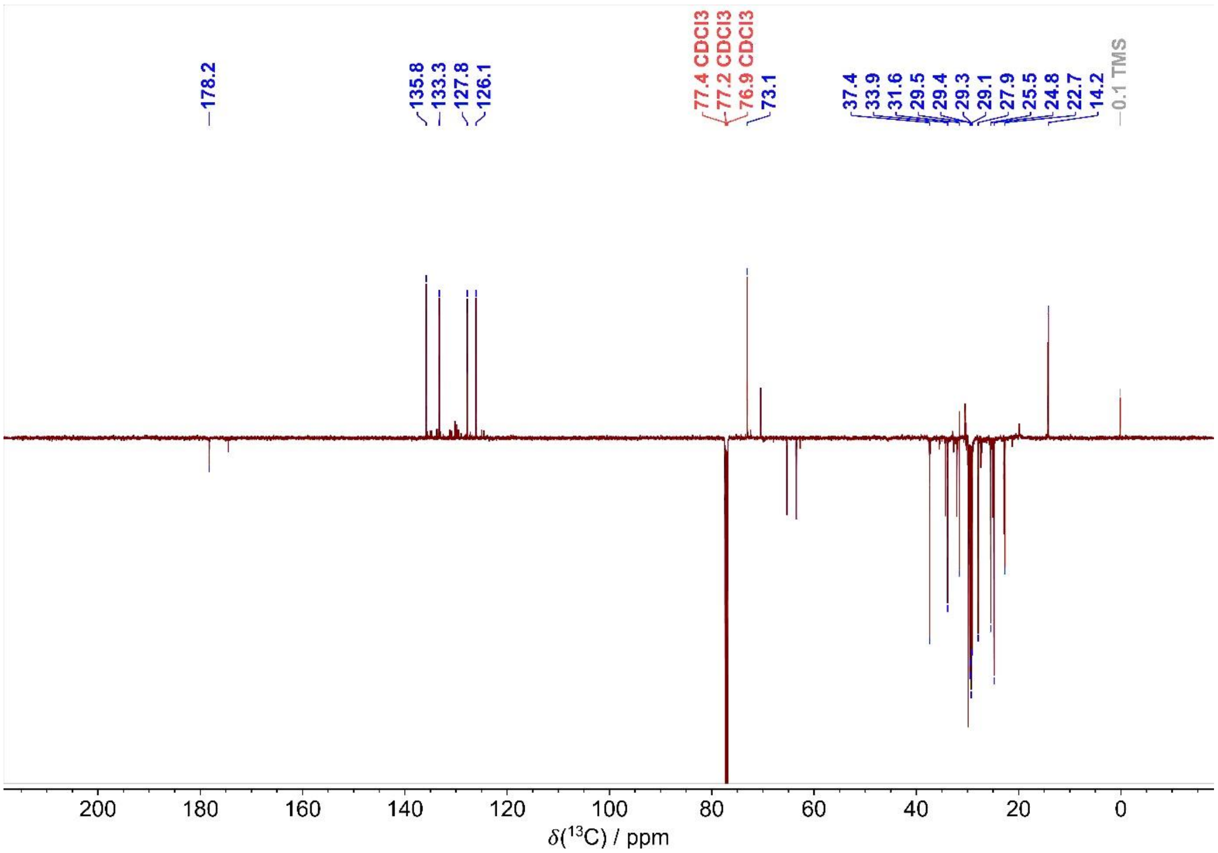

Figure S15. ^{13}C DEPTQ NMR spectrum of (10*E*,12*Z*)-9-hydroxy-10,12-octadecadienoic acid (**A2**) ($CDCl_3$, 151 MHz).

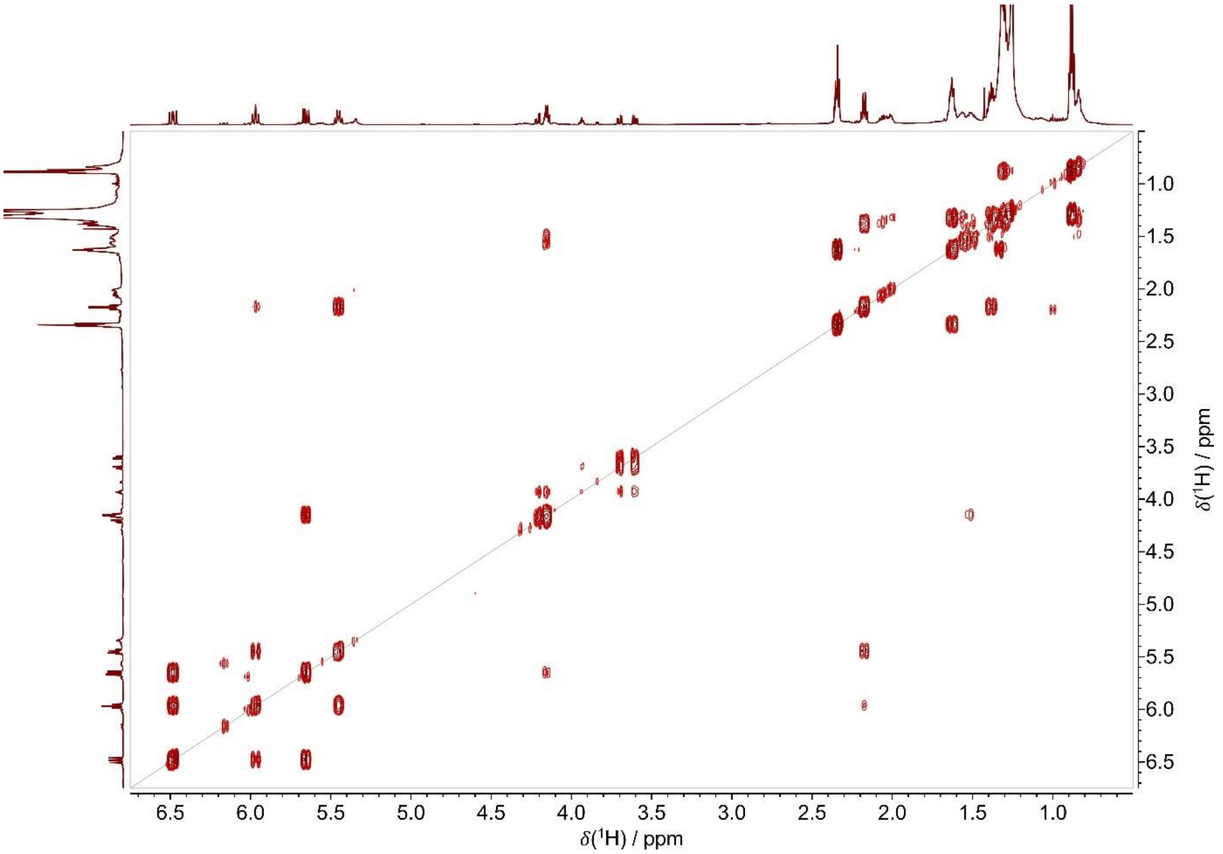

Figure S16. ^{1}H–^{1}H COSY NMR spectrum of (10*E*,12*Z*)-9-hydroxy-10,12-octadecadienoic acid (**A2**) ($CDCl_3$, 600 MHz).

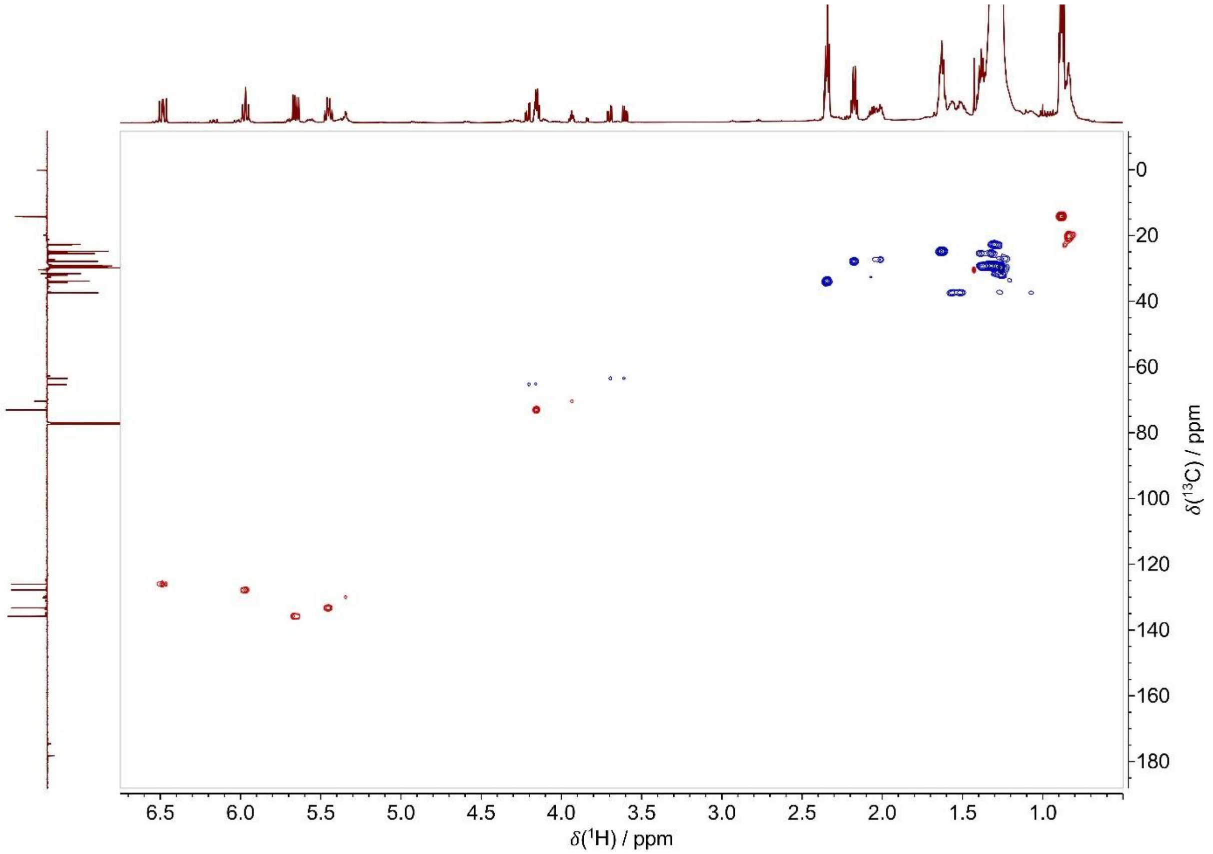

Figure S17. ^{1}H–^{13}C edHSQC NMR spectrum of (10*E*,12*Z*)-9-hydroxy-10,12-octadecadienoic acid (**A2**) ($CDCl_3$, 600 and 151 MHz).

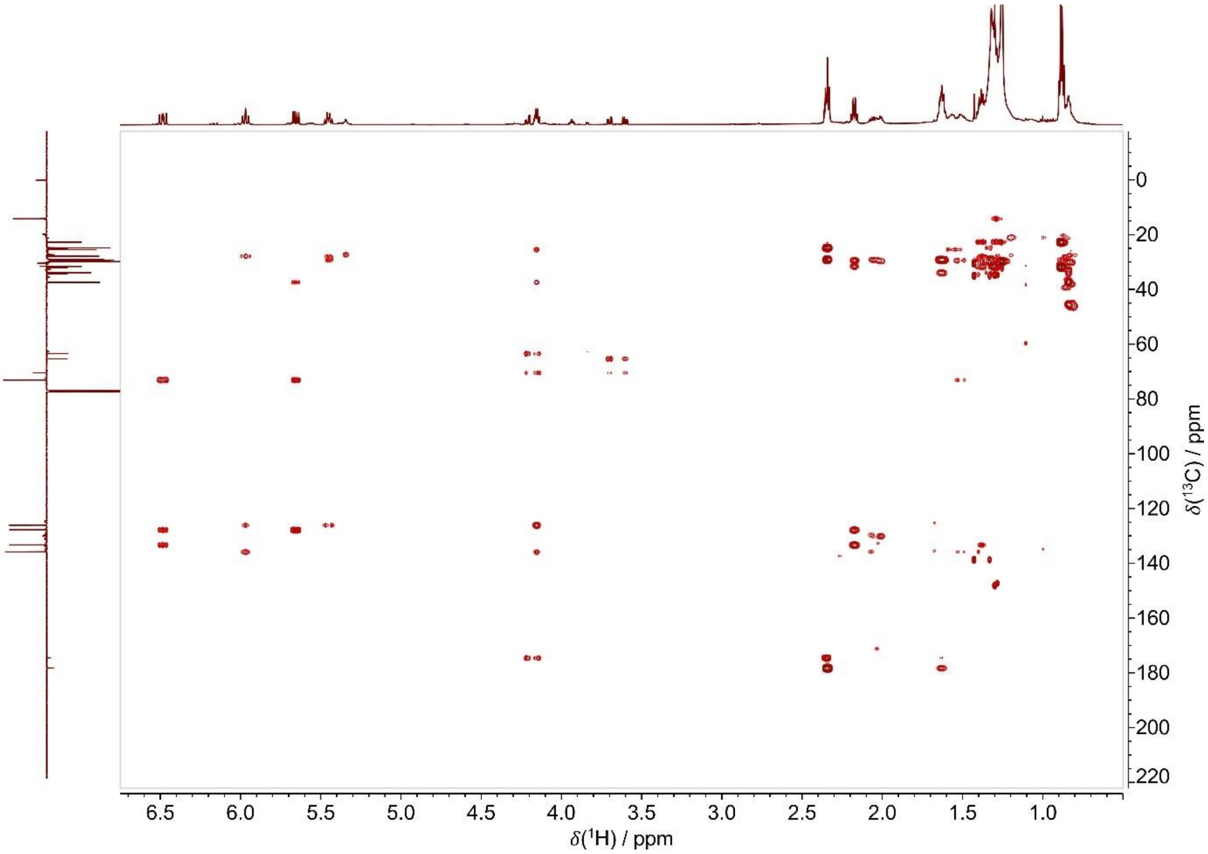

Figure S18. ^{1}H–^{13}C HMBC NMR spectrum of (10*E*,12*Z*)-9-hydroxy-10,12-octadecadienoic acid (**A2**) ($CDCl_3$, 600 and 151 MHz).

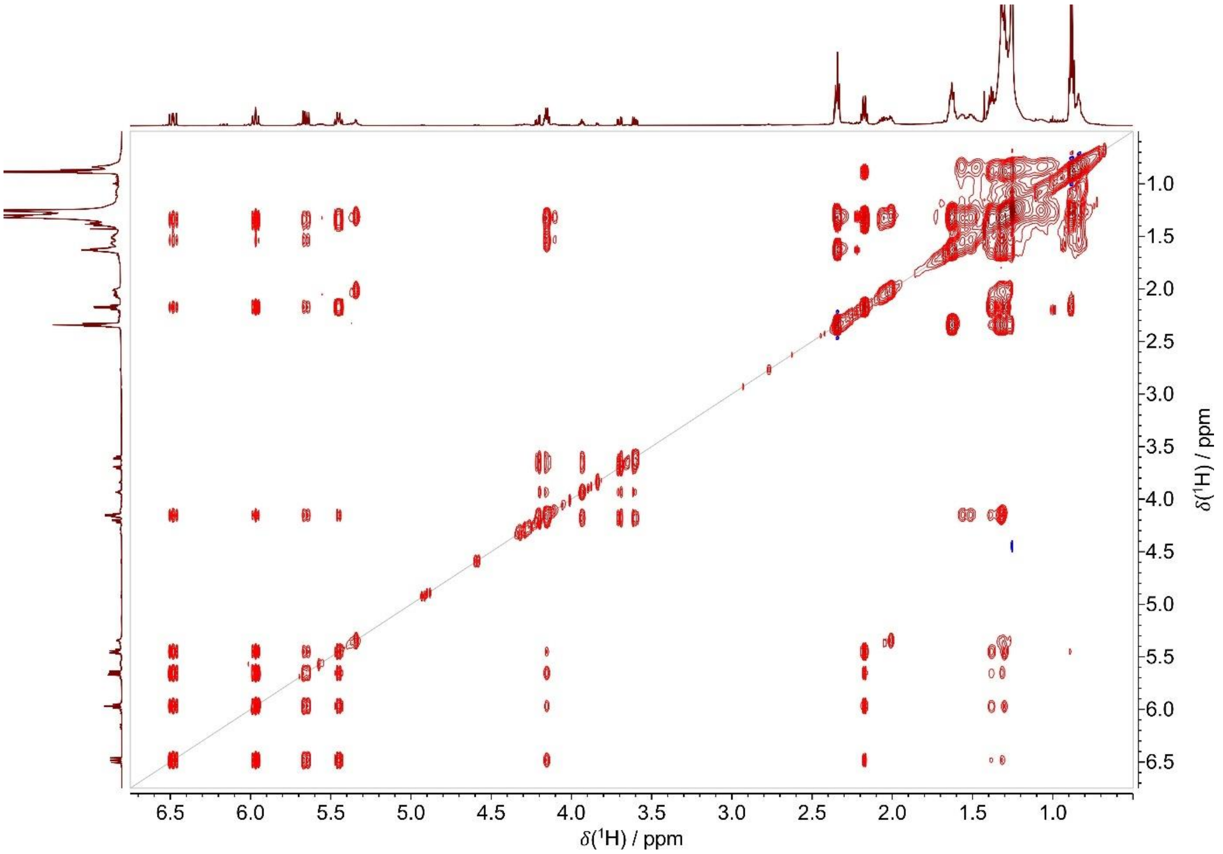

Figure S19. ^{1}H–^{1}H TOCSY NMR spectrum of (10*E*,12*Z*)-9-hydroxy-10,12-octadecadienoic acid (**A2**) ($CDCl_3$, 600 MHz).

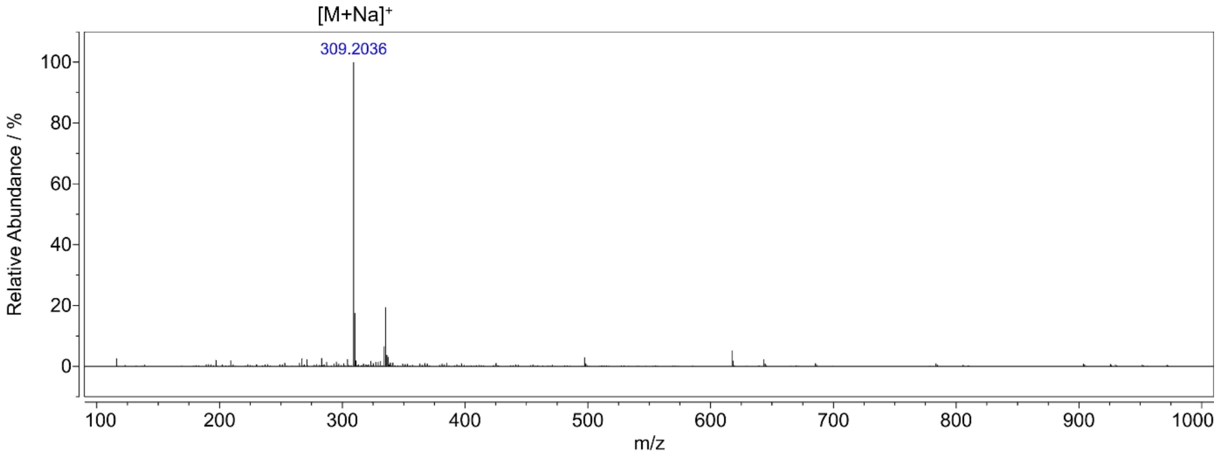

Figure S20. HR-ESI$^+$-MS spectrum of hexadecanedioic acid (**A3**), *m*/*z* 309.2036 [M+Na]$^+$ (calculated for $C_{16}H_{30}O_4Na^+$, *m*/*z* 309.2036 [M+Na]$^+$, error: –0.1 ppm).

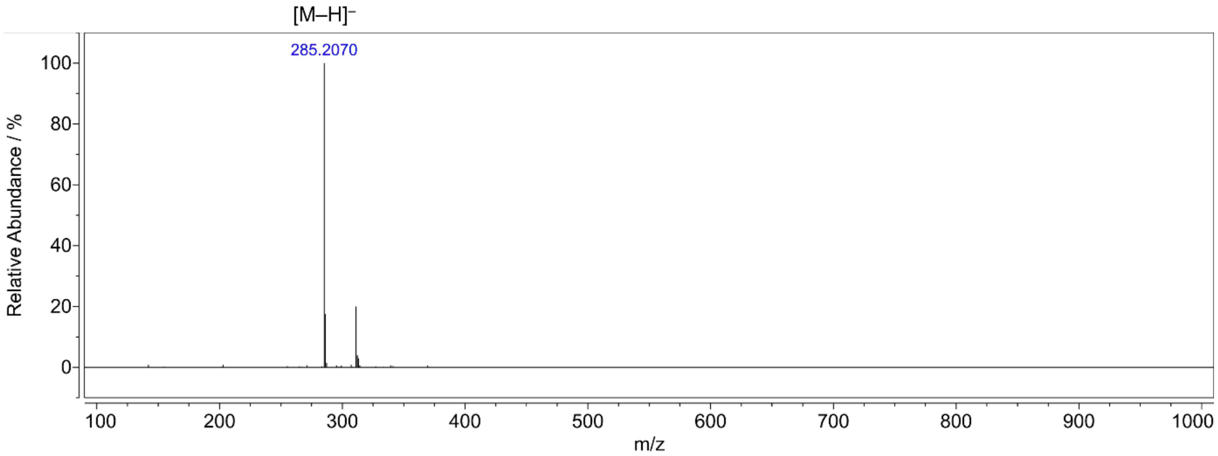

Figure S21. HR-ESI⁻-MS spectrum of hexadecanedioic acid (**A3**), *m*/*z* 285.2070 [M–H]⁻ (calculated for $C_{16}H_{29}O_4^-$, *m*/*z* 285.2071 [M–H]⁻, error: –0.5 ppm).

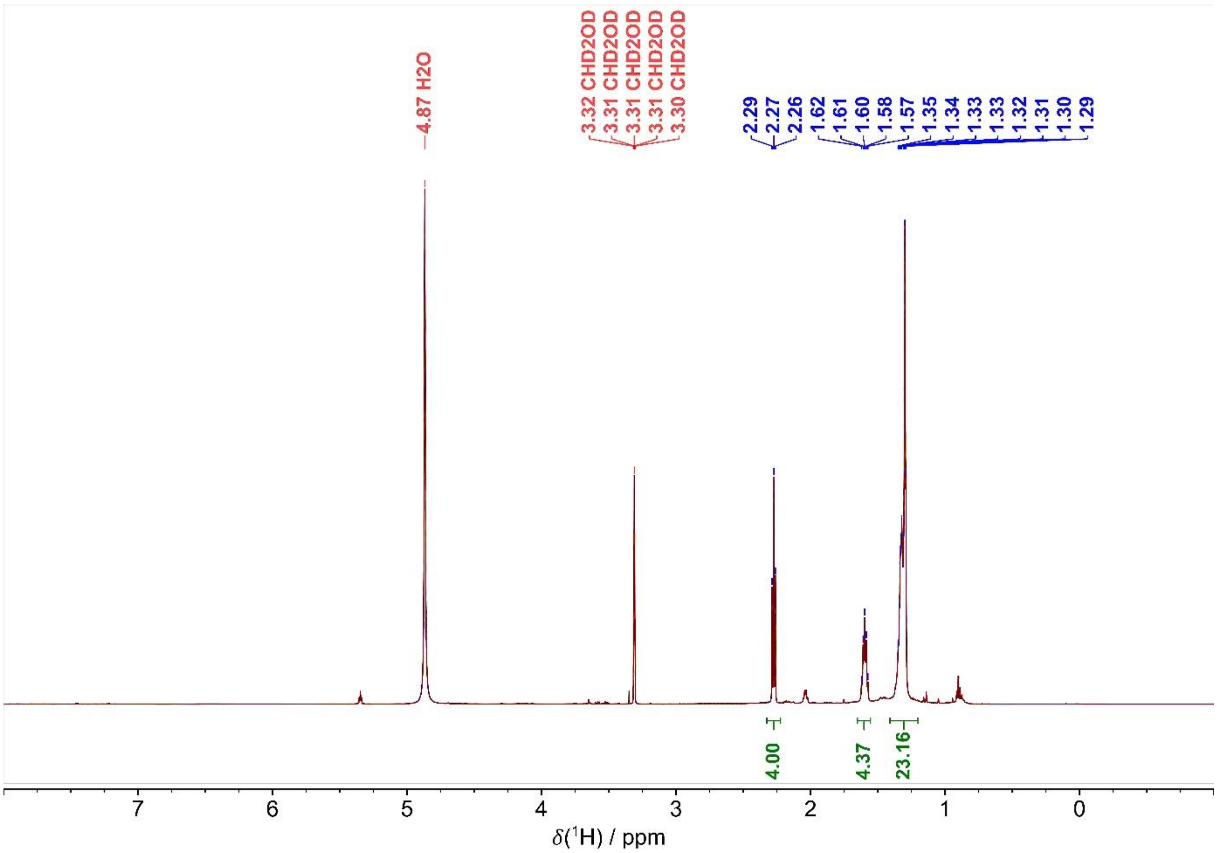

Figure S22. ^{1}H NMR spectrum of hexadecanedioic acid (**A3**) (CD_3OD, 600 MHz).

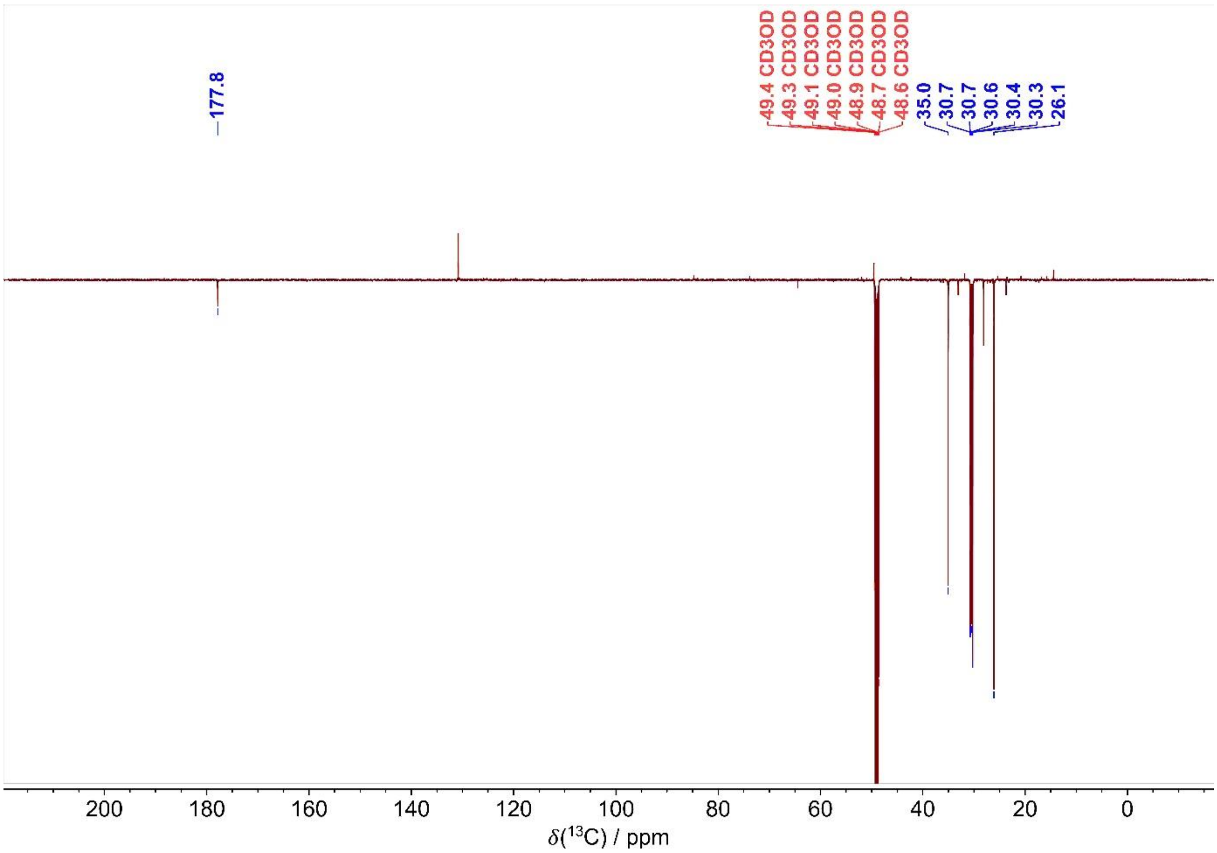

Figure S23. ^{13}C DEPTQ NMR spectrum of hexadecanedioic acid (**A3**) (CD_3OD, 151 MHz).

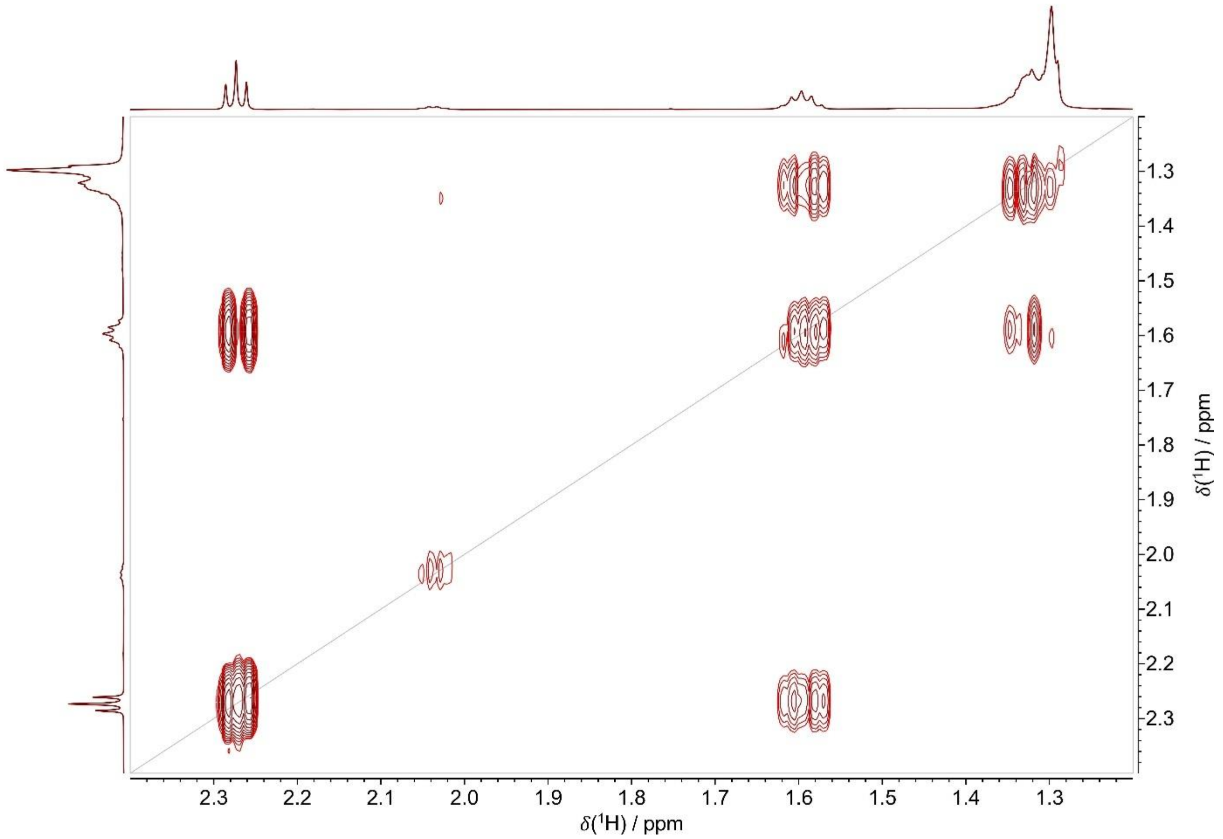

Figure S24. ^1H–^1H COSY NMR spectrum of hexadecanedioic acid (**A3**) (CD_3OD, 600 MHz).

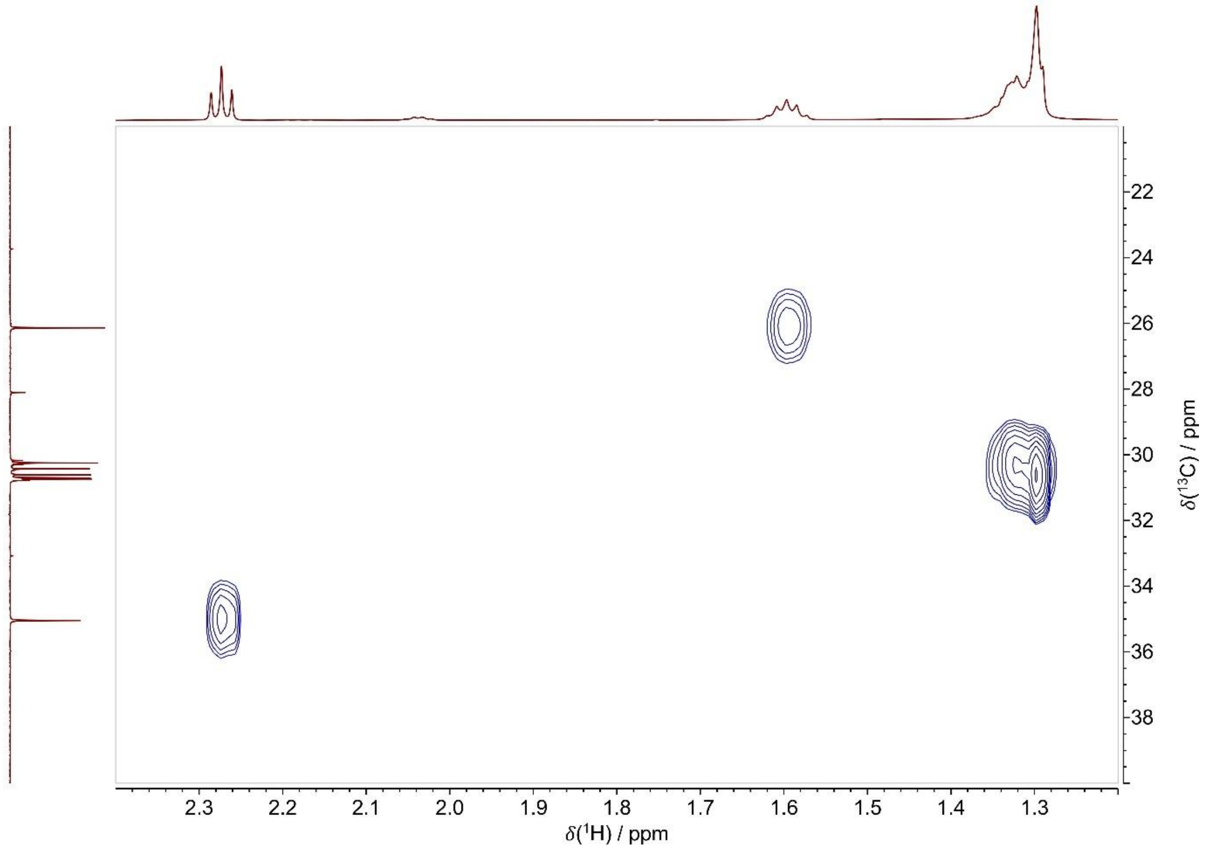

Figure S25. ^{1}H–^{13}C edHSQC NMR spectrum of hexadecanedioic acid (**A3**) (CD_3OD, 600 and 151 MHz).

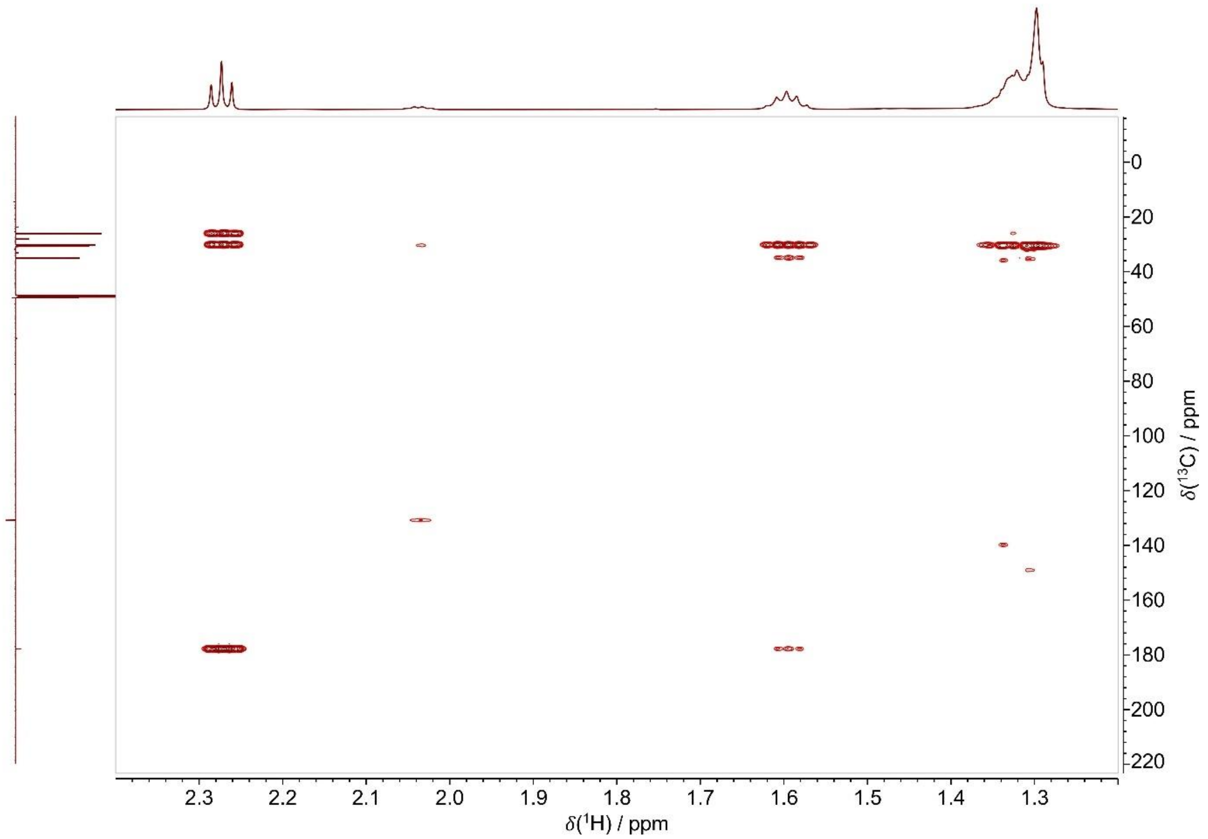

Figure S26. ^{1}H–^{13}C HMBC NMR spectrum of hexadecanedioic acid (**A3**) (CD_3OD, 600 and 151 MHz).

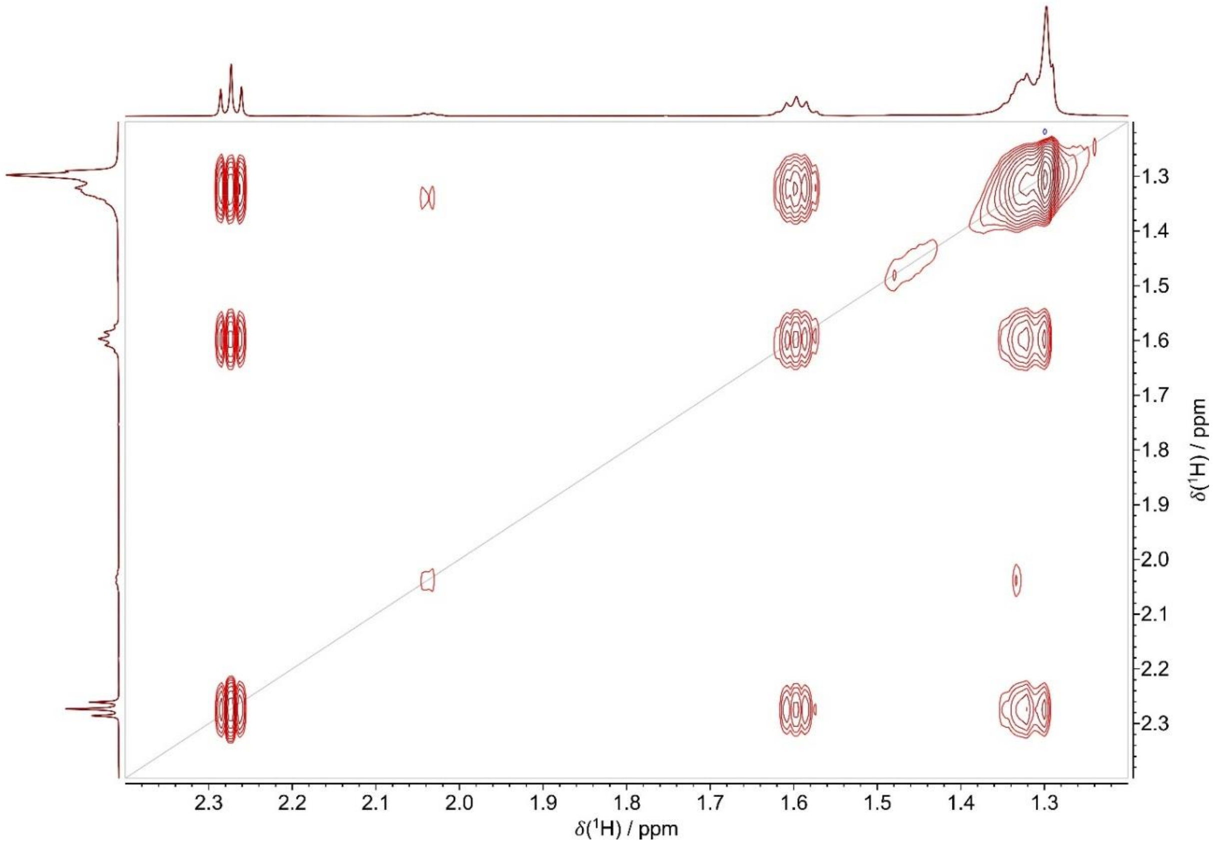

Figure S27. ^{1}H–^{1}H TOCSY NMR spectrum of hexadecanedioic acid (**A3**) (CD_3OD, 600 MHz).

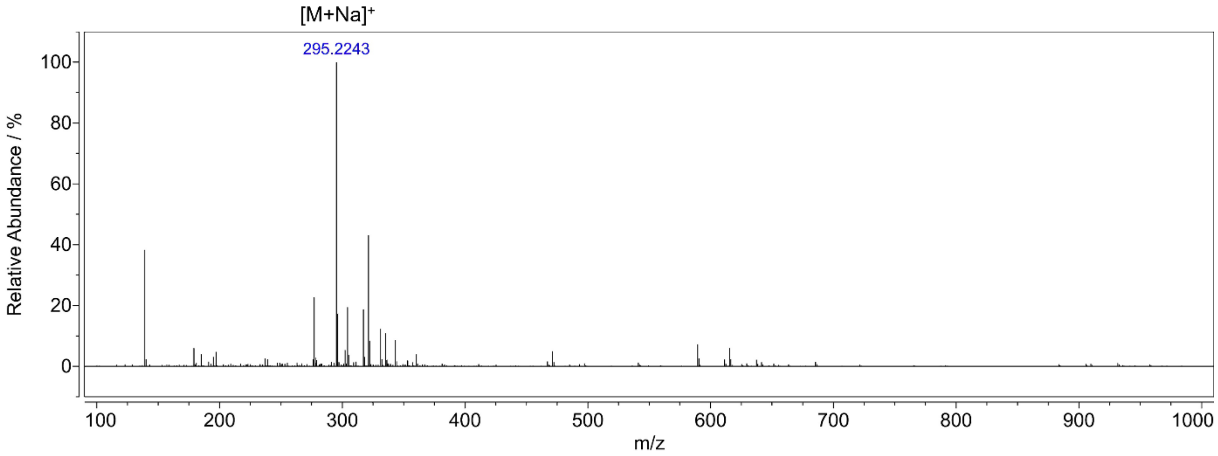

Figure S28. HR-ESI$^+$-MS spectrum of 16-hydroxyhexadecanoic acid (**A4**), *m*/*z* 295.2243 [M+Na]$^+$ (calculated for $C_{16}H_{32}O_3Na^+$, *m*/*z* 295.2244 [M+Na]$^+$, error: –0.2 ppm).

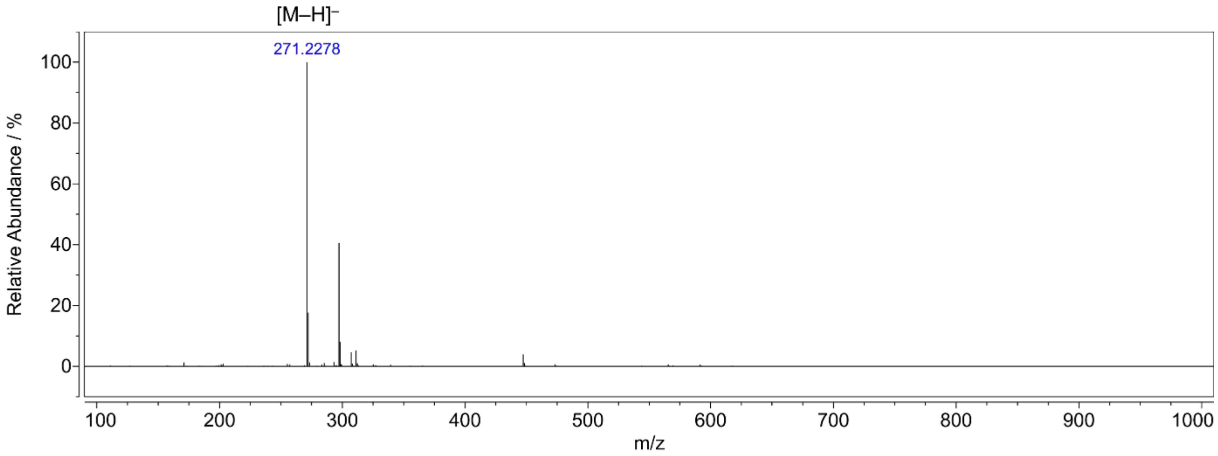

Figure S29. HR-ESI$^-$-MS spectrum of 16-hydroxyhexadecanoic acid (**A4**), *m*/*z* 271.2278 [M–H]$^-$ (calculated for $C_{16}H_{31}O_3^-$, *m*/*z* 271.2279 [M–H]$^-$, error: –0.3 ppm).

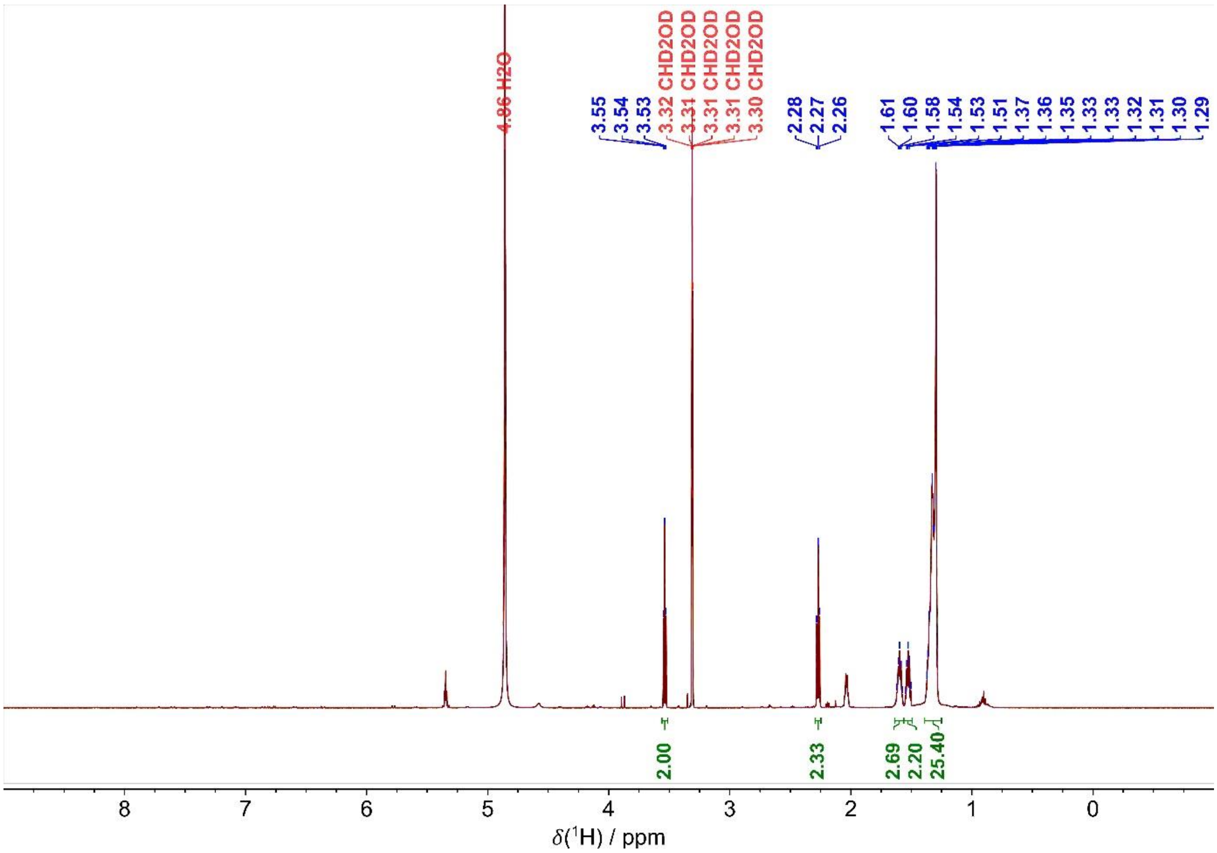

Figure S30. ^{1}H NMR spectrum of 16-hydroxyhexadecanoic acid (**A4**) (CD_3OD, 600 MHz).

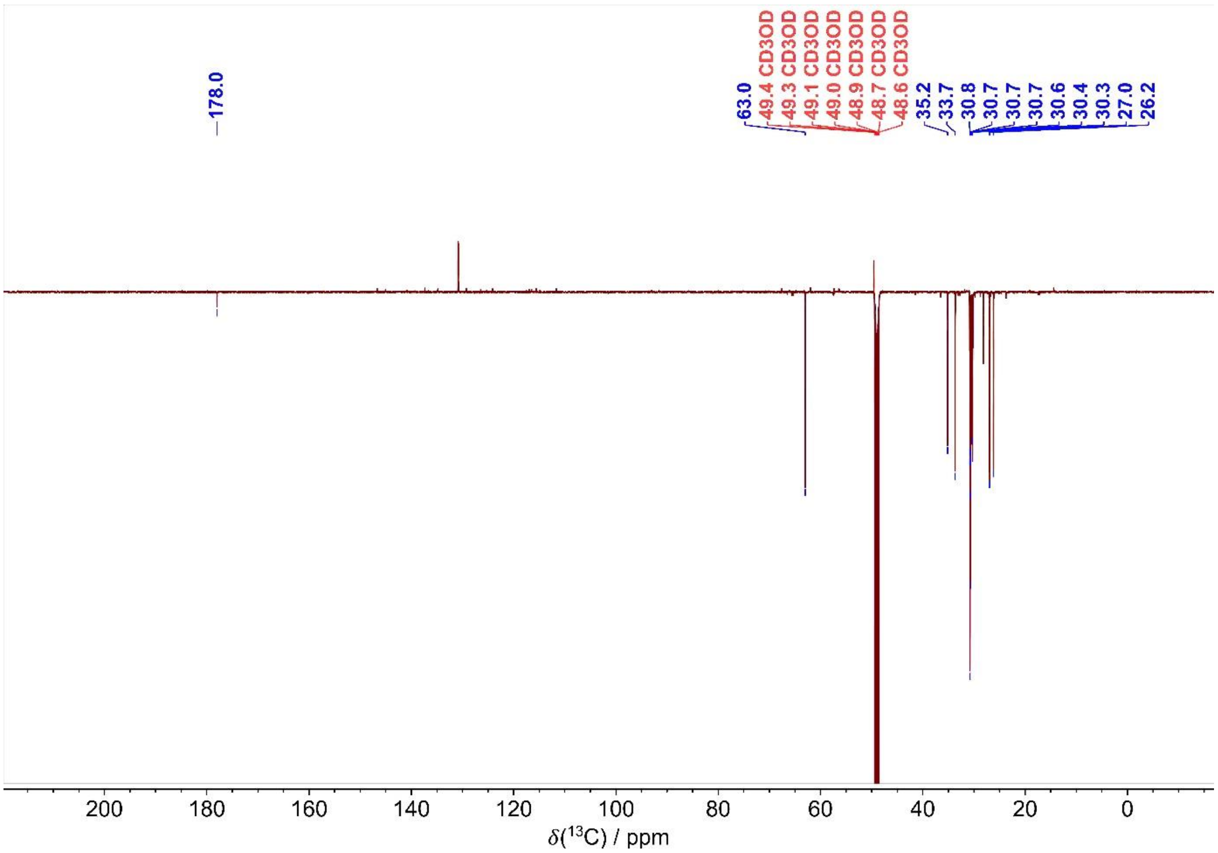

Figure S31. ^{13}C DEPTQ NMR spectrum of 16-hydroxyhexadecanoic acid (**A4**) (CD_3OD, 151 MHz).

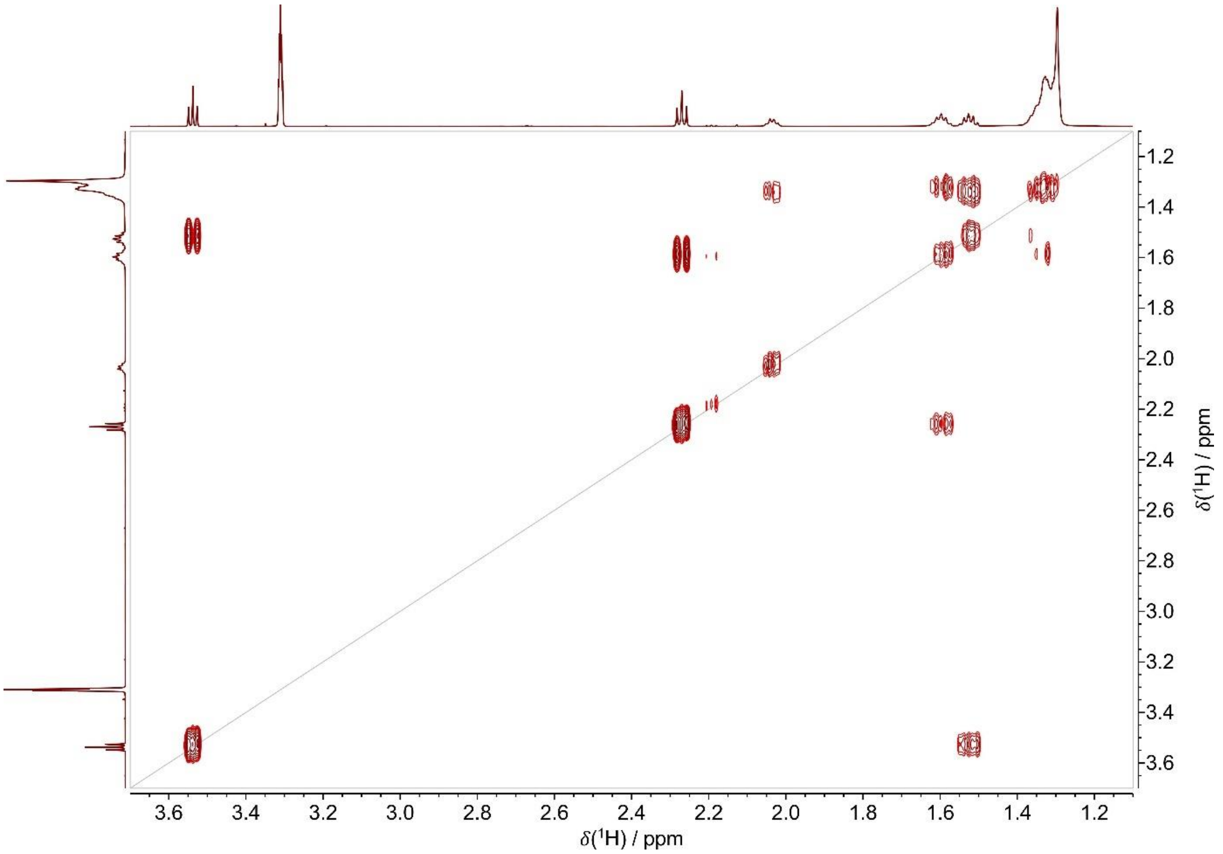

Figure S32. ^{1}H–^{1}H COSY NMR spectrum of 16-hydroxyhexadecanoic acid (**A4**) (CD_3OD, 600 MHz).

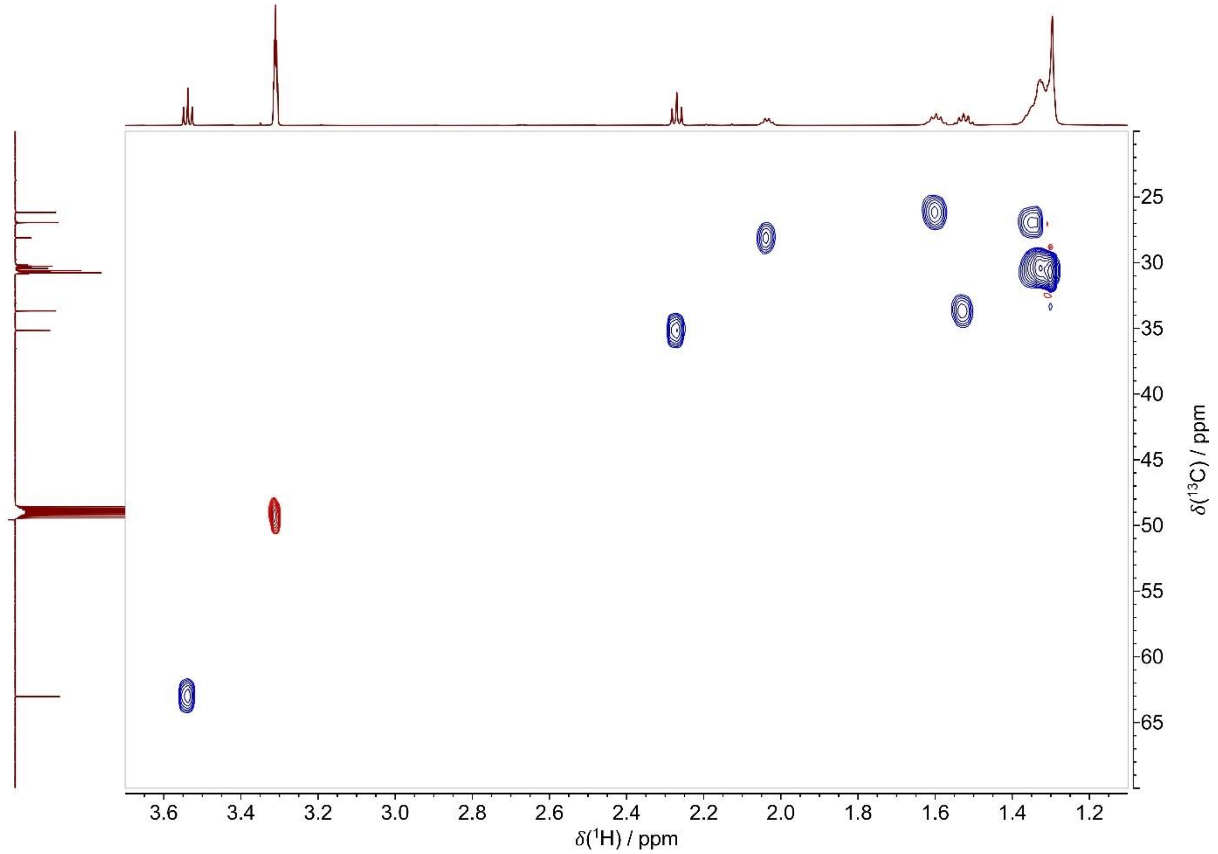

Figure S33. ^{1}H–^{13}C edHSQC NMR spectrum of 16-hydroxyhexadecanoic acid (**A4**) (CD_3OD, 600 and 151 MHz).

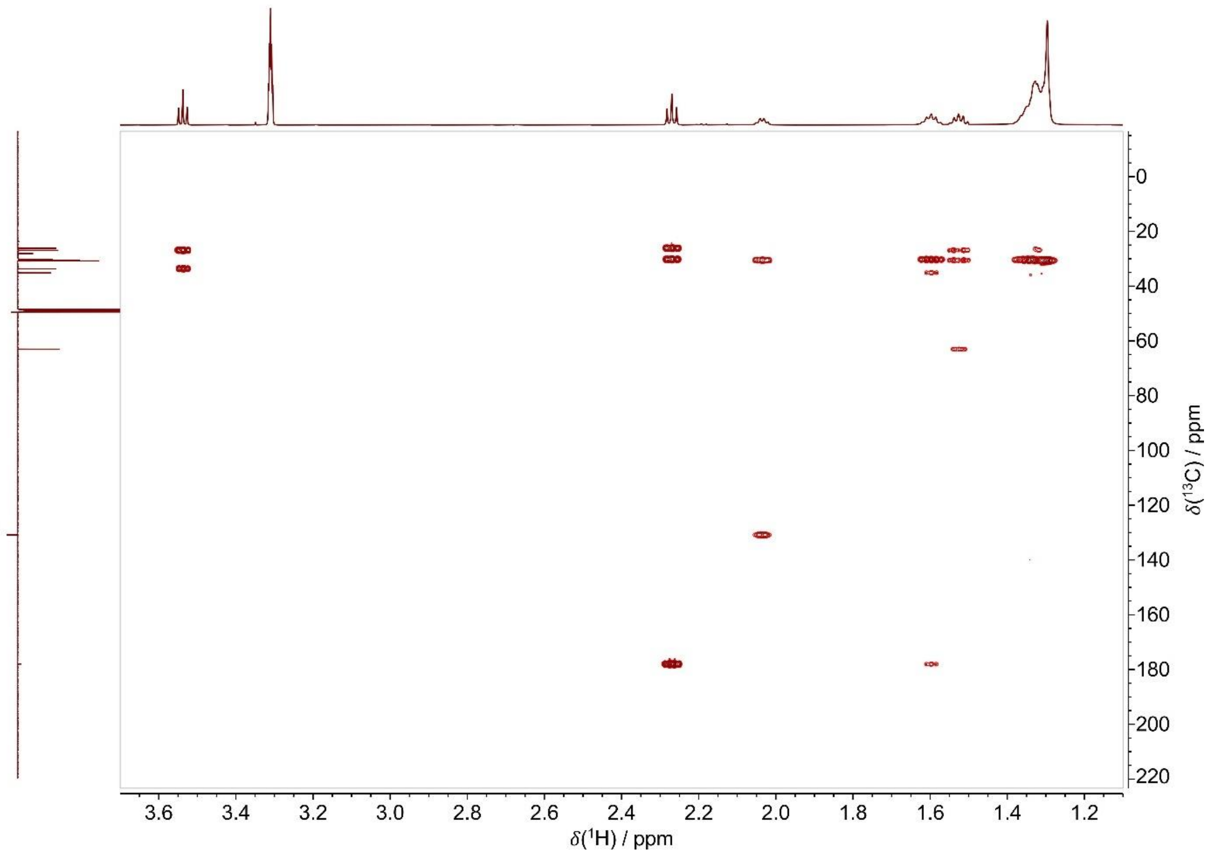

Figure S34. ^{1}H–^{13}C HMBC NMR spectrum of 16-hydroxyhexadecanoic acid (**A4**) (CD_3OD, 600 and 151 MHz).

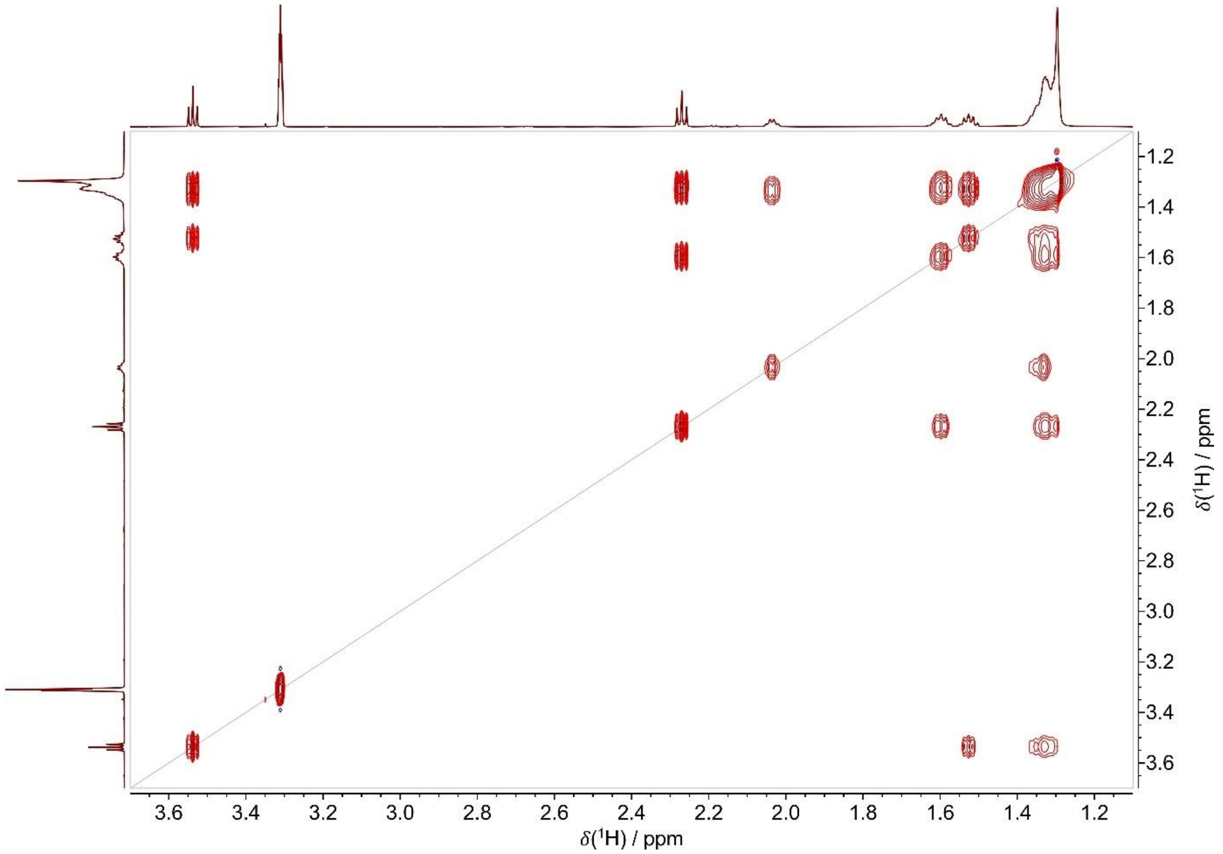

Figure S35. ^{1}H–^{1}H TOCSY NMR spectrum of 16-hydroxyhexadecanoic acid (**A4**) (CD_3OD, 600 MHz).

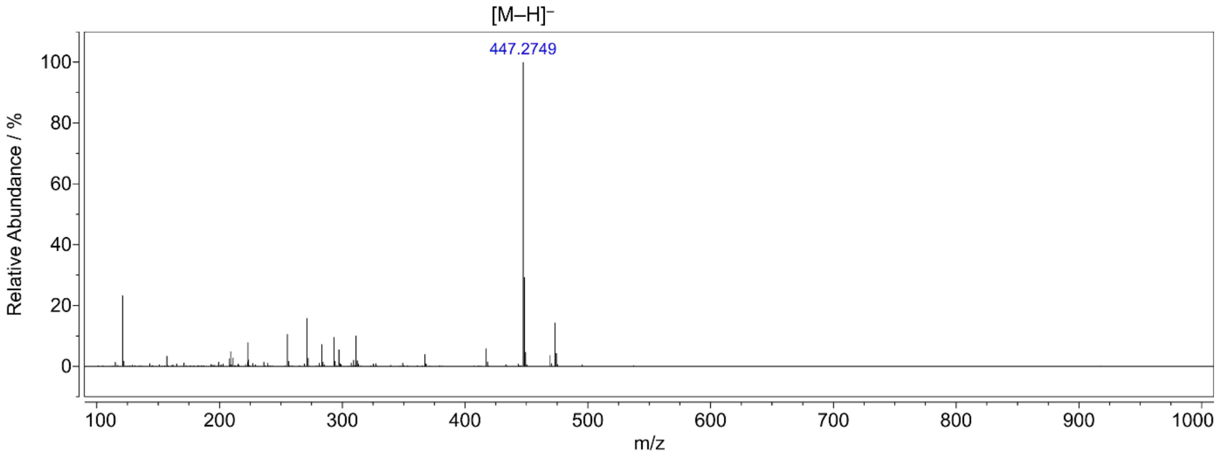

Figure S36. HR-ESI⁻-MS spectrum of alpinagalanate (**A5**), *m*/*z* 447.2749 [M–H]⁻ (calculated for $C_{26}H_{39}O_6^-$, *m*/*z* 447.2752 [M–H]⁻, error: –0.7 ppm).

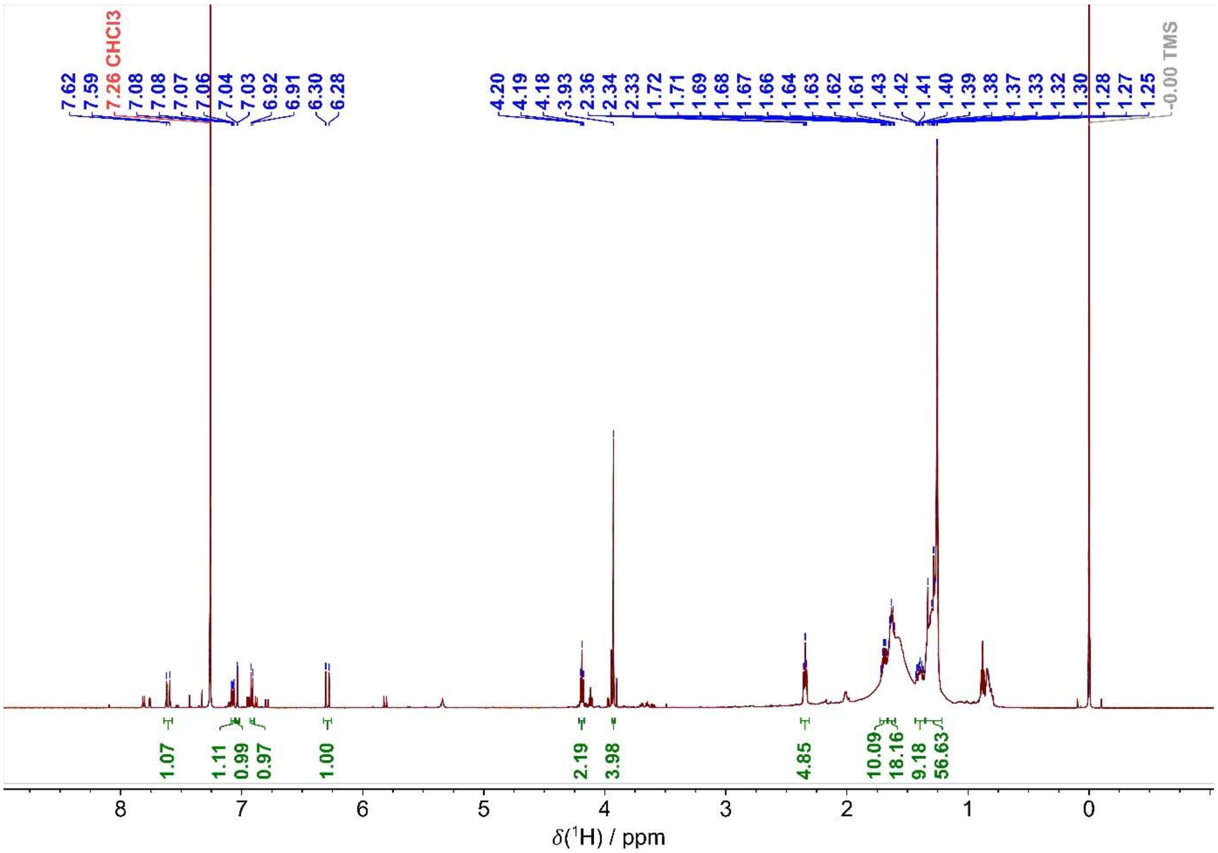

Figure S37. ^{1}H NMR spectrum of alpinagalanate (**A5**) ($CDCl_3$, 600 MHz).

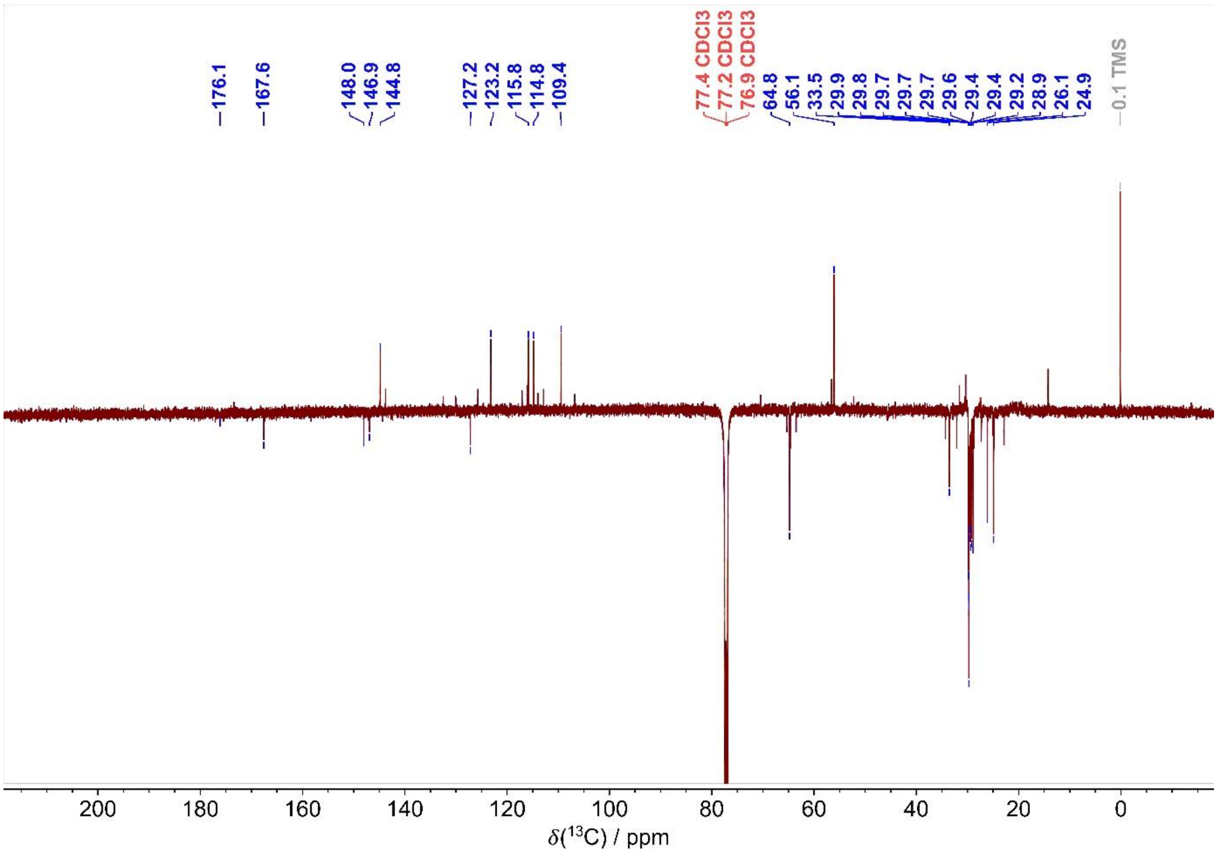

Figure S38. ^{13}C DEPTQ NMR spectrum of alpinagalanate (**A5**) ($CDCl_3$, 151 MHz).

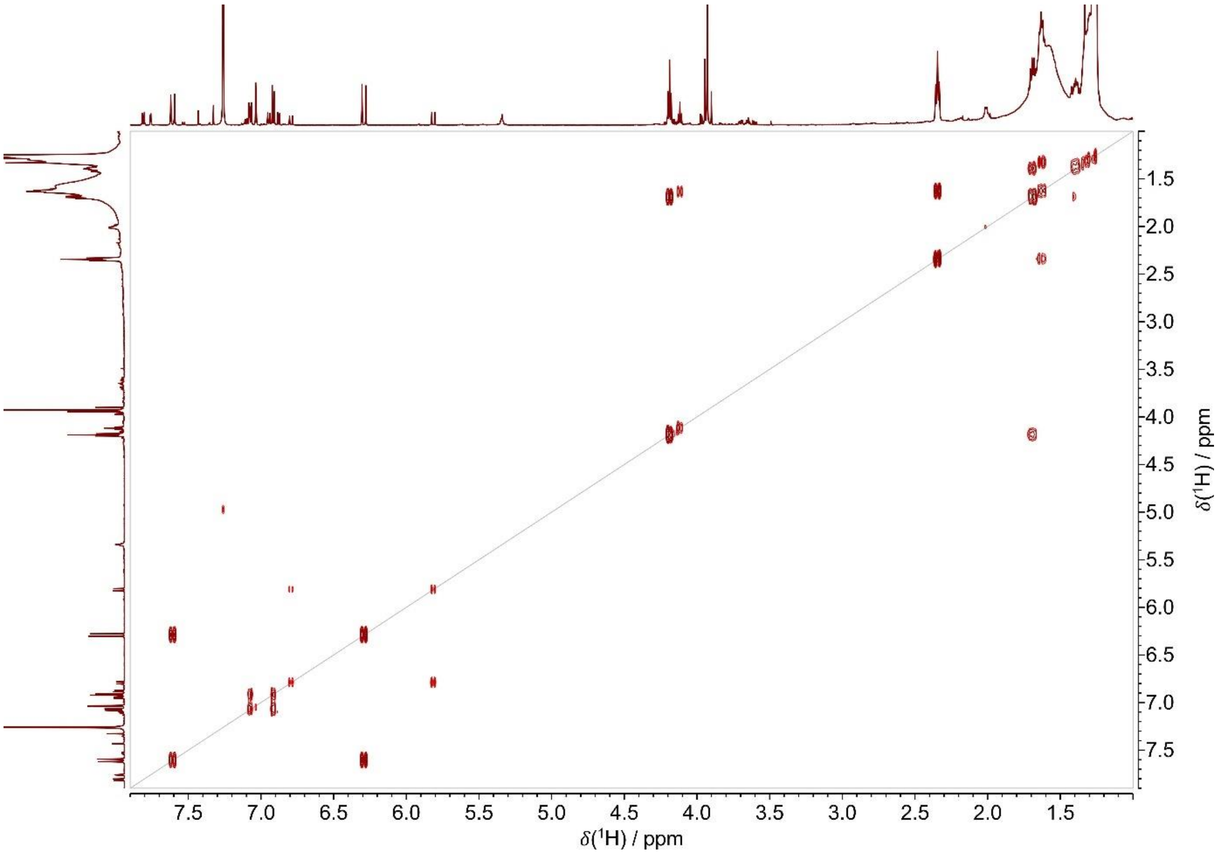

Figure S39. ^1H–^1H COSY NMR spectrum of alpinagalanate (**A5**) ($CDCl_3$, 600 MHz).

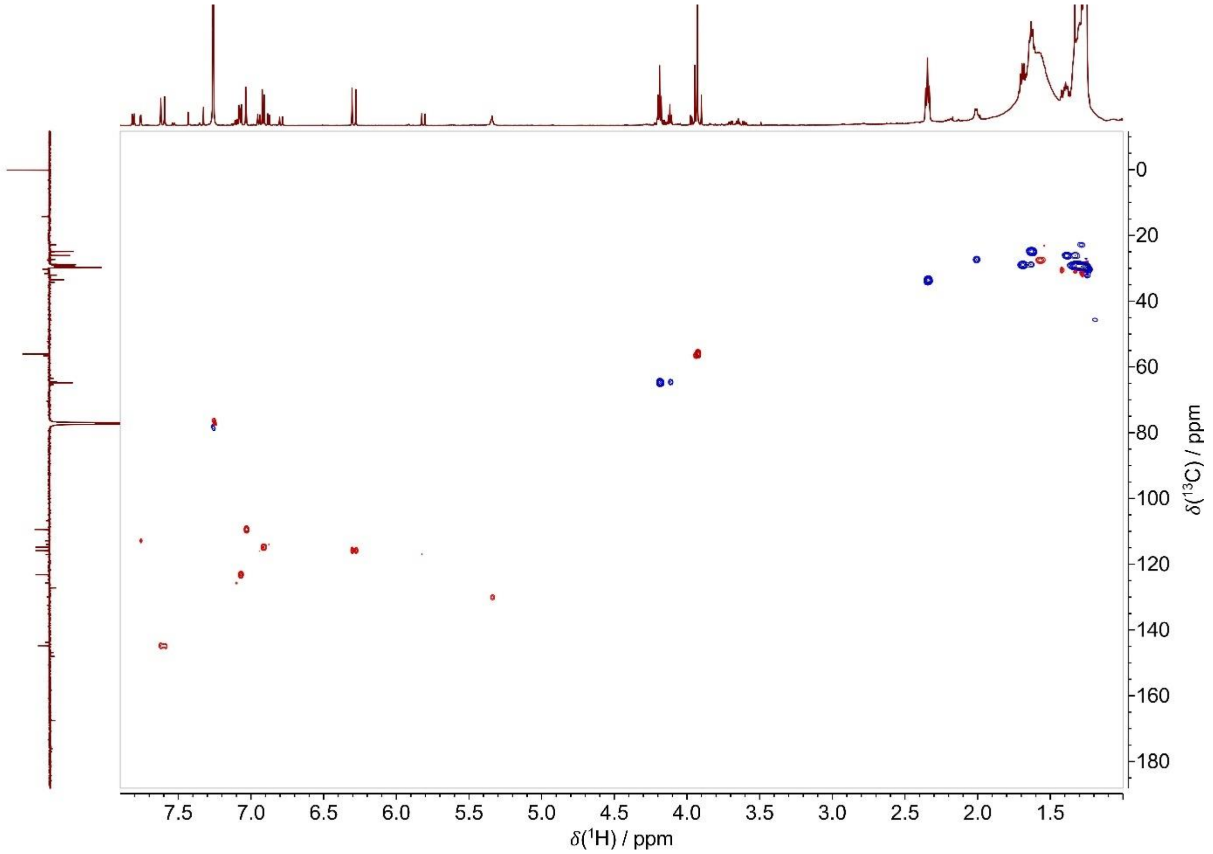

Figure S40. ^{1}H–^{13}C edHSQC NMR spectrum of alpinagalanate (**A5**) ($CDCl_3$, 600 and 151 MHz).

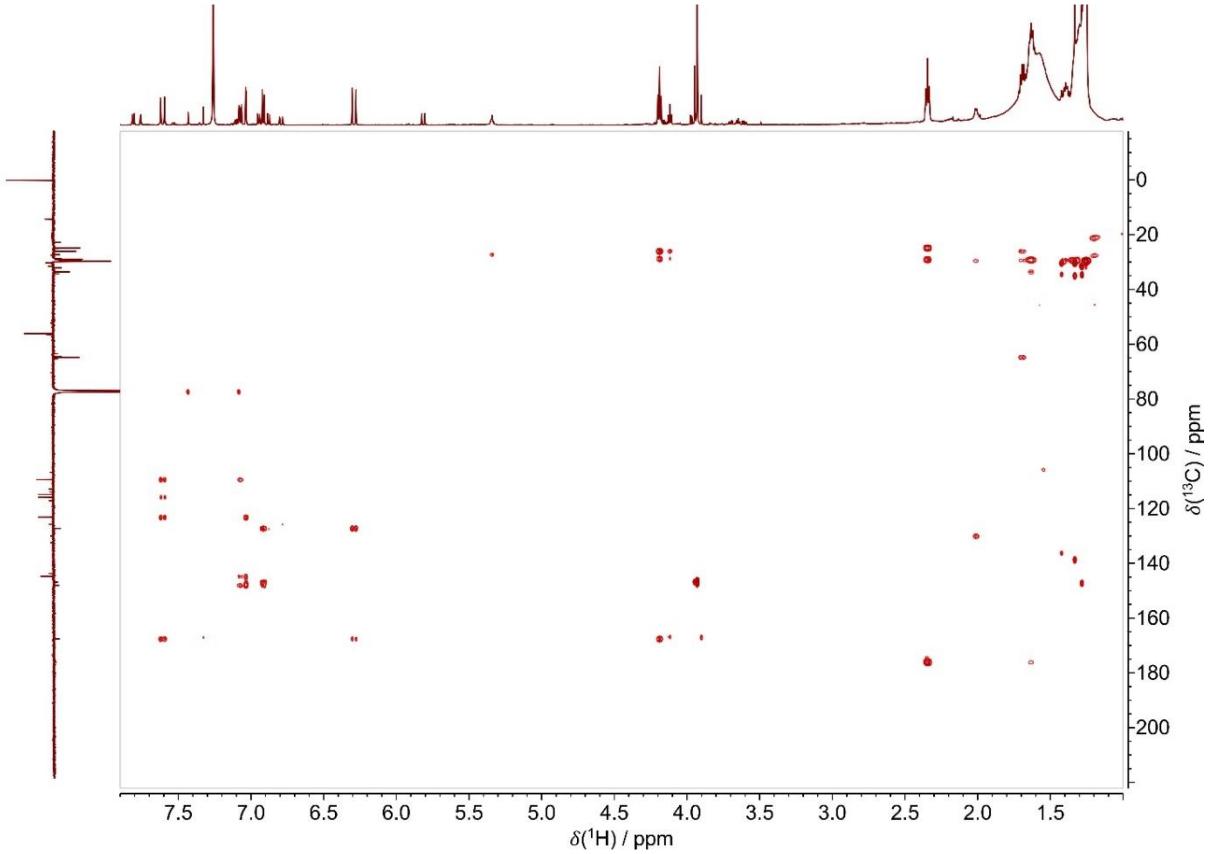

Figure S41. ^{1}H–^{13}C HMBC NMR spectrum of alpinagalanate (**A5**) ($CDCl_3$, 600 and 151 MHz).

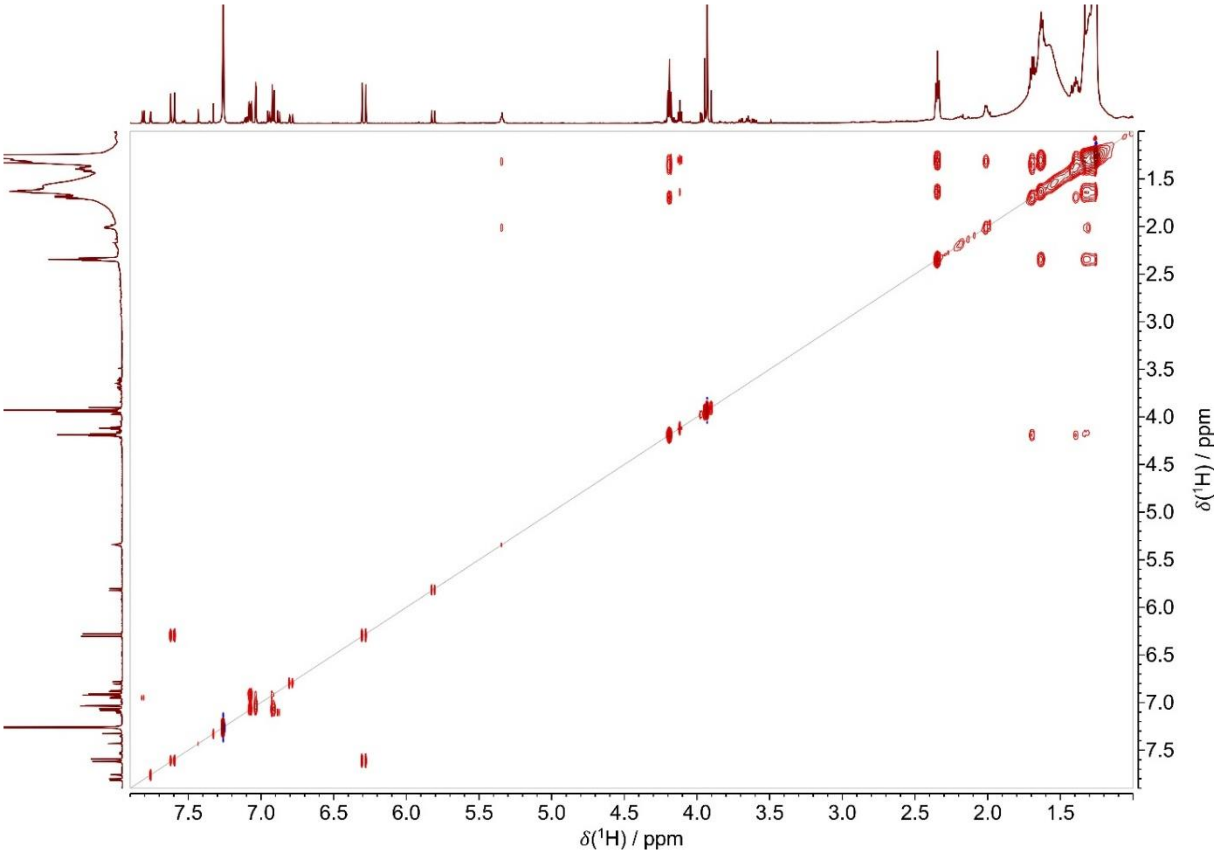

Figure S42. ^{1}H–^{1}H TOCSY NMR spectrum of alpinagalanate (**A5**) ($CDCl_3$, 600 MHz).

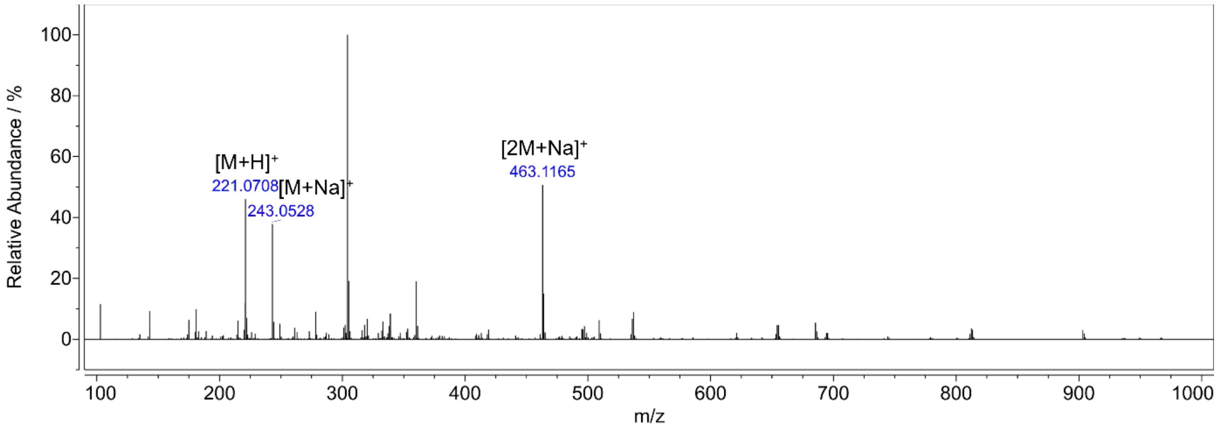

Figure S43. HR-ESI$^+$-MS spectrum of canthin-6-one (**A6**), *m*/*z* 243.0528 [M+Na]$^+$ (calculated for $C_{14}H_8N_2ONa^+$, *m*/*z* 243.0529 [M+Na]$^+$, error: –0.3 ppm); *m*/*z* 221.0708 [M+H]$^+$ (calculated for $C_{14}H_9N_2O^+$, *m*/*z* 221.0709 [M+H]$^+$, error: –0.6 ppm).

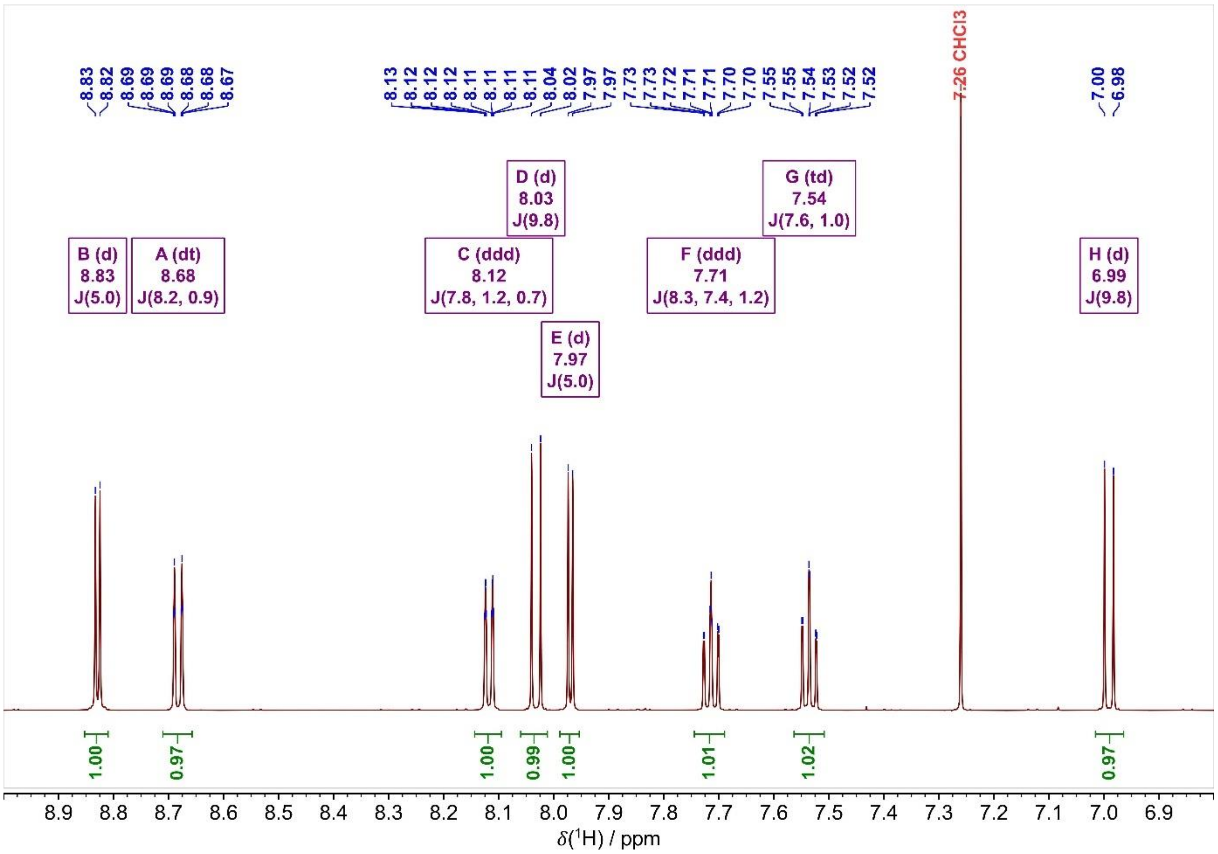

Figure S44. ^{1}H NMR spectrum of canthin-6-one (**A6**) ($CDCl_3$, 600 MHz).

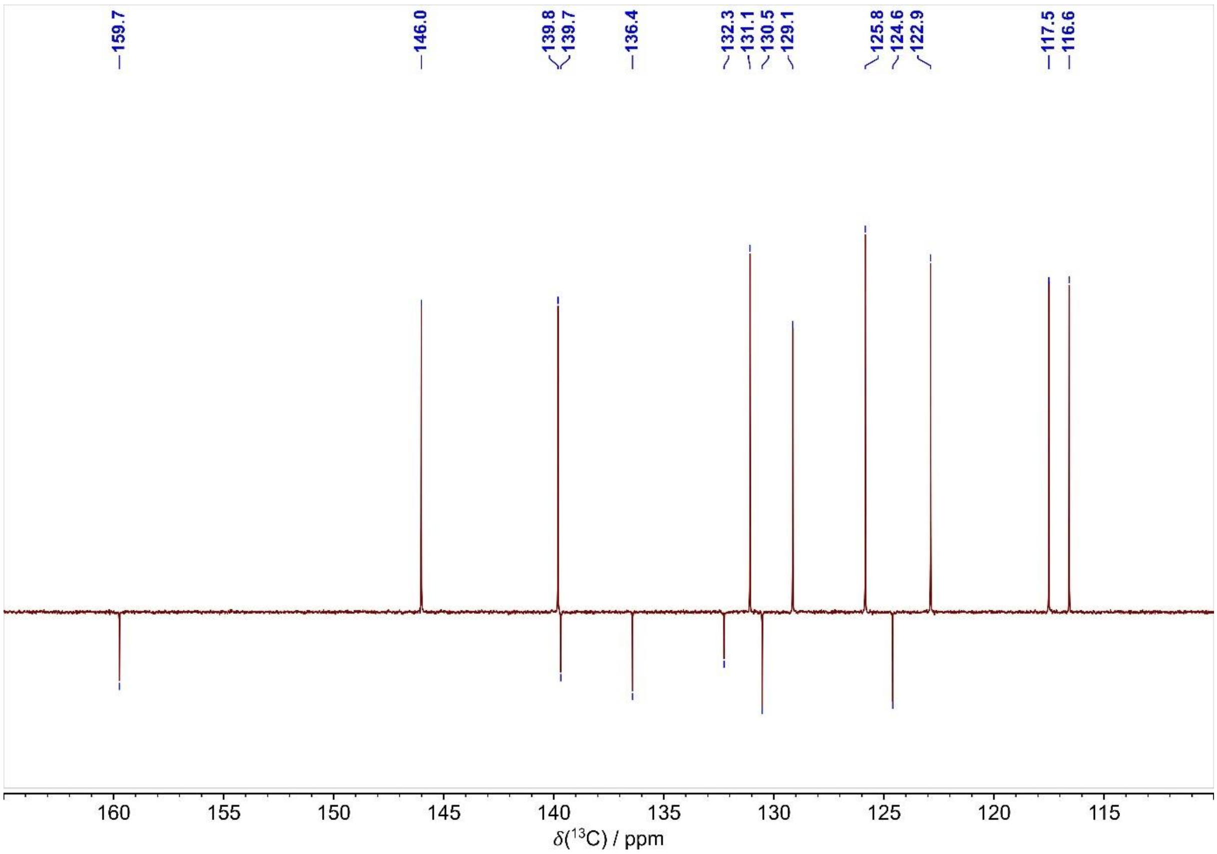

Figure S45. ^{13}C DEPTQ NMR spectrum of canthin-6-one (**A6**) ($CDCl_3$, 151 MHz).

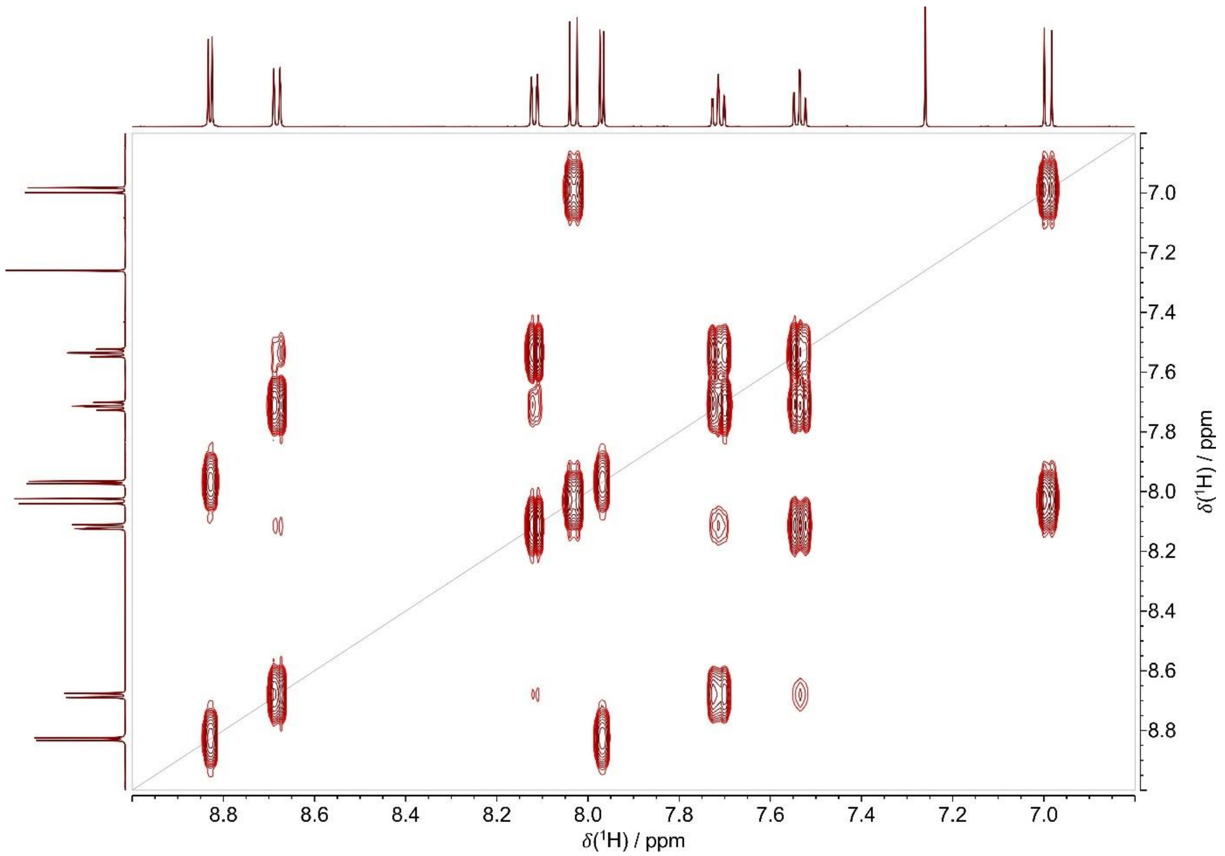

Figure S46. ^{1}H–^{1}H COSY NMR spectrum of canthin-6-one (**A6**) ($CDCl_3$, 600 MHz).

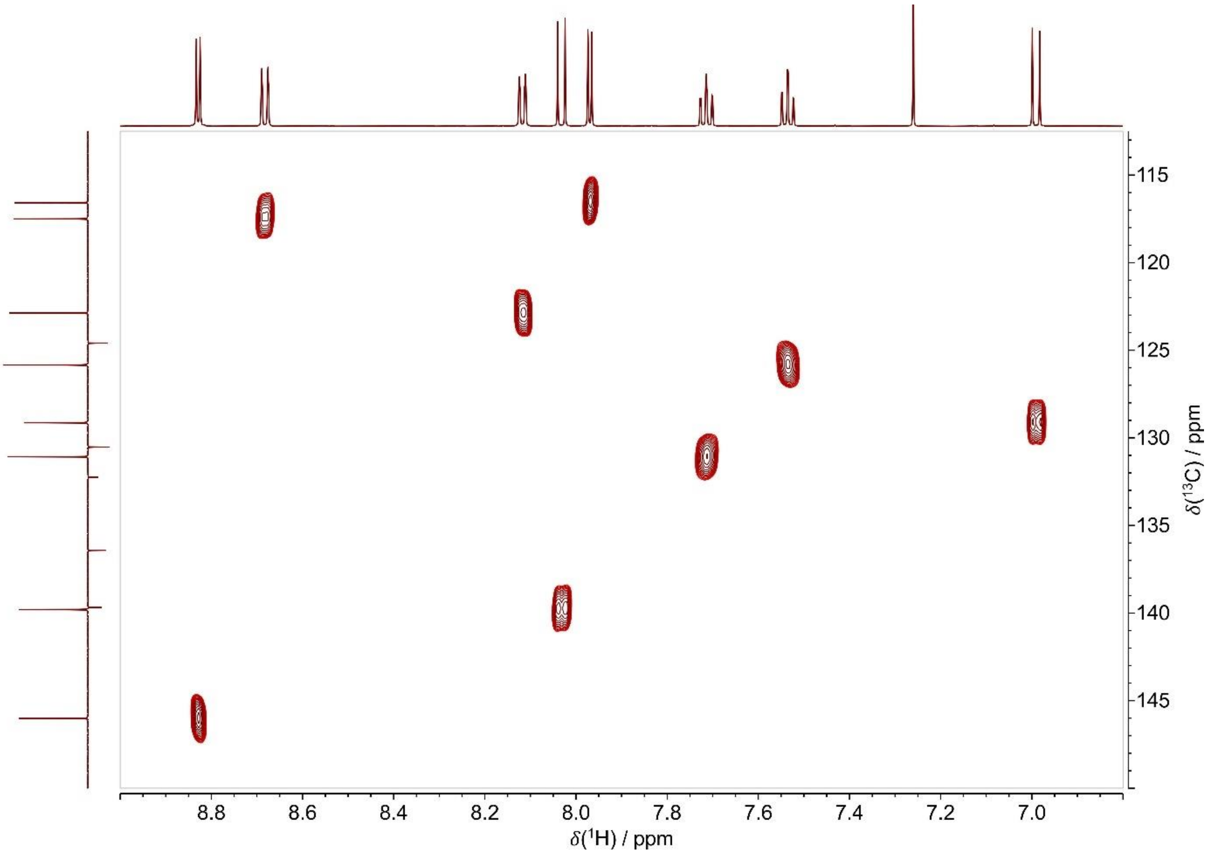

Figure S47. ^1H–^{13}C edHSQC NMR spectrum of canthin-6-one (**A6**) ($CDCl_3$, 600 and 151 MHz).

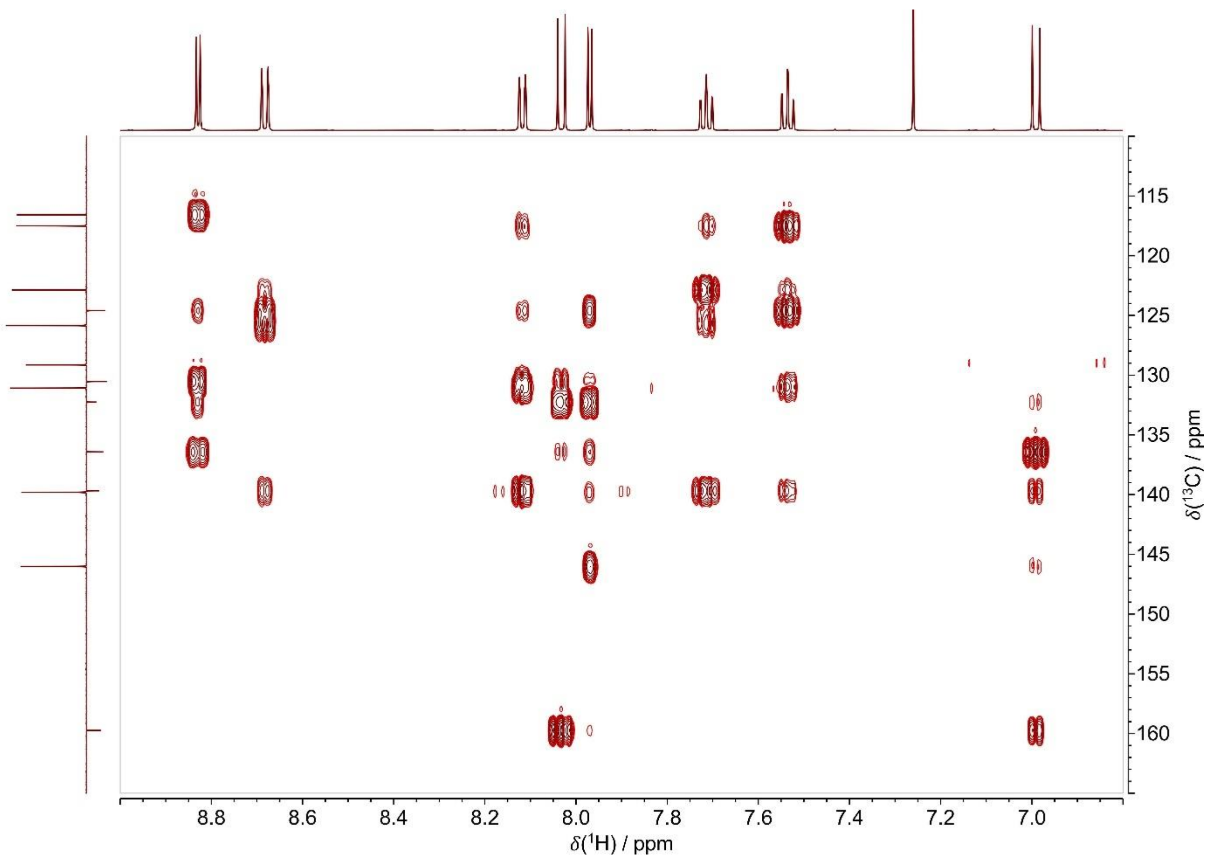

Figure S48. ^{1}H–^{13}C HMBC NMR spectrum of canthin-6-one (**A6**) ($CDCl_3$, 600 and 151 MHz).

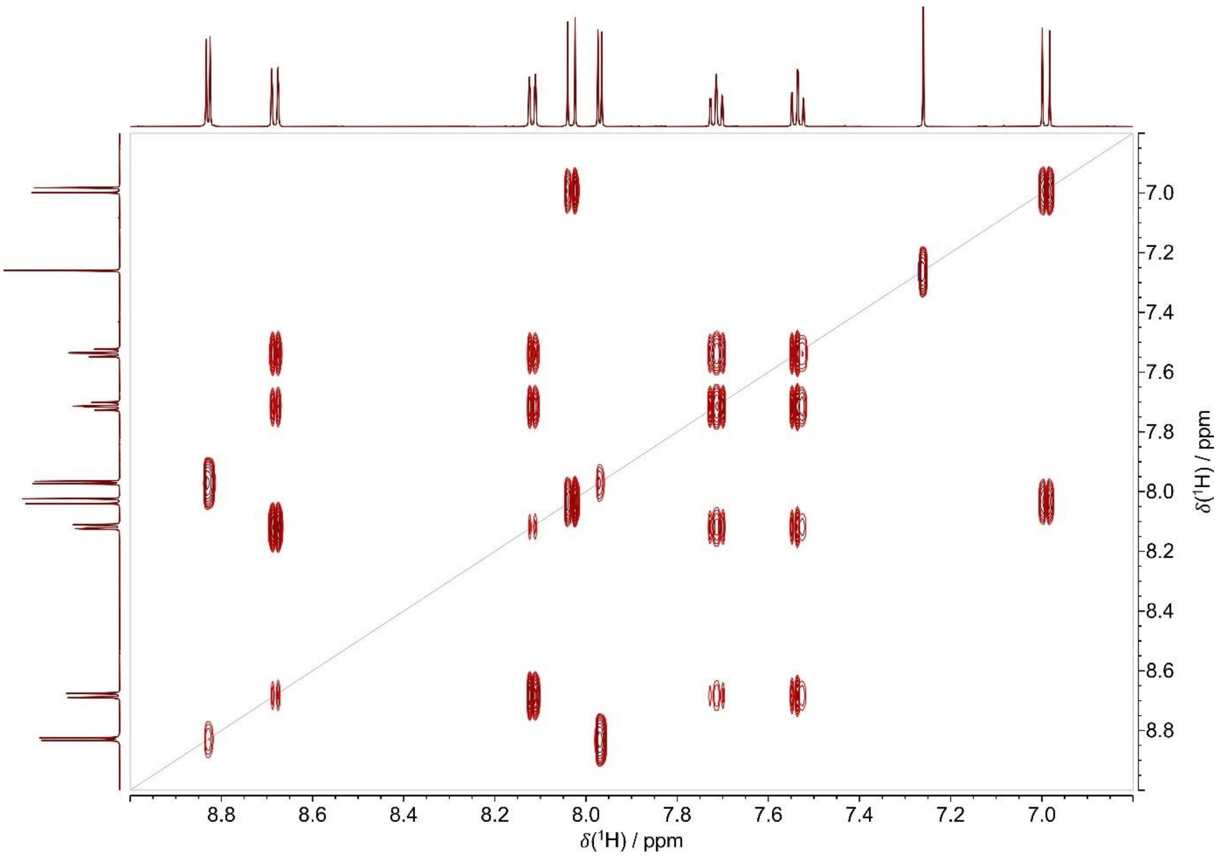

Figure S49. ^{1}H–^{1}H TOCSY NMR spectrum of canthin-6-one (**A6**) ($CDCl_3$, 600 MHz).
